# Genome-wide characterization of the sorghum *JAZ* gene family and their responses to phytohormone treatments and aphid infestation

**DOI:** 10.1038/s41598-022-07181-9

**Published:** 2022-02-25

**Authors:** Kumar Shrestha, Yinghua Huang

**Affiliations:** 1 Department of Plant Biology, Ecology and Evolution, Oklahoma State University, Stillwater, OK 74078 USA; 2 Plant Science Research Laboratory, United States Department of Agriculture-Agricultural Research Service (USDA-ARS), Stillwater, OK 74075 USA

**Keywords:** Genetics, Molecular biology, Plant sciences

## Abstract

Jasmonate ZIM-domain (JAZ) proteins are the key repressors of the jasmonic acid (JA) signal transduction pathway and play a crucial role in stress-related defense, phytohormone crosstalk and modulation of the growth-defense tradeoff. In this study, the sorghum genome was analyzed through genome-wide comparison and domain scan analysis, which led to the identification of 18 sorghum *JAZ* (*SbJAZ*) genes. All SbJAZ proteins possess the conserved TIFY and Jas domains and they formed a phylogenetic tree with five clusters related to the orthologs of other plant species. Similarly, evolutionary analysis indicated the duplication events as a major force of expansion of the *SbJAZ* genes and there was strong neutral and purifying selection going on. In silico analysis of the promoter region of the *SbJAZ* genes indicates that *SbJAZ5, SbJAZ6, SbJAZ13, SbJAZ16 *and* SbJAZ17* are rich in stress-related *cis*-elements. In addition, expression profiling of the *SbJAZ* genes in response to phytohormones treatment (JA, ET, ABA, GA) and sugarcane aphid (SCA) was performed in two recombinant inbred lines (RILs) of sorghum, resistant (RIL 521) and susceptible (RIL 609) to SCA. Taken together, data generated from phytohormone expression and in silico analysis suggests the putative role of *SbJAZ9* in JA-ABA crosstalk and *SbJAZ16* in JA-ABA and JA-GA crosstalk to regulate certain physiological processes. Notably, upregulation of *SbJAZ1, SbJAZ5, SbJAZ13 *and* SbJAZ16* in resistant RIL during JA treatment and SCA infestation suggests putative functions in stress-related defense and to balance the plant defense to promote growth. Overall, this report provides valuable insight into the organization and functional characterization of the sorghum *JAZ* gene family.

## Introduction

**Figure 1:**
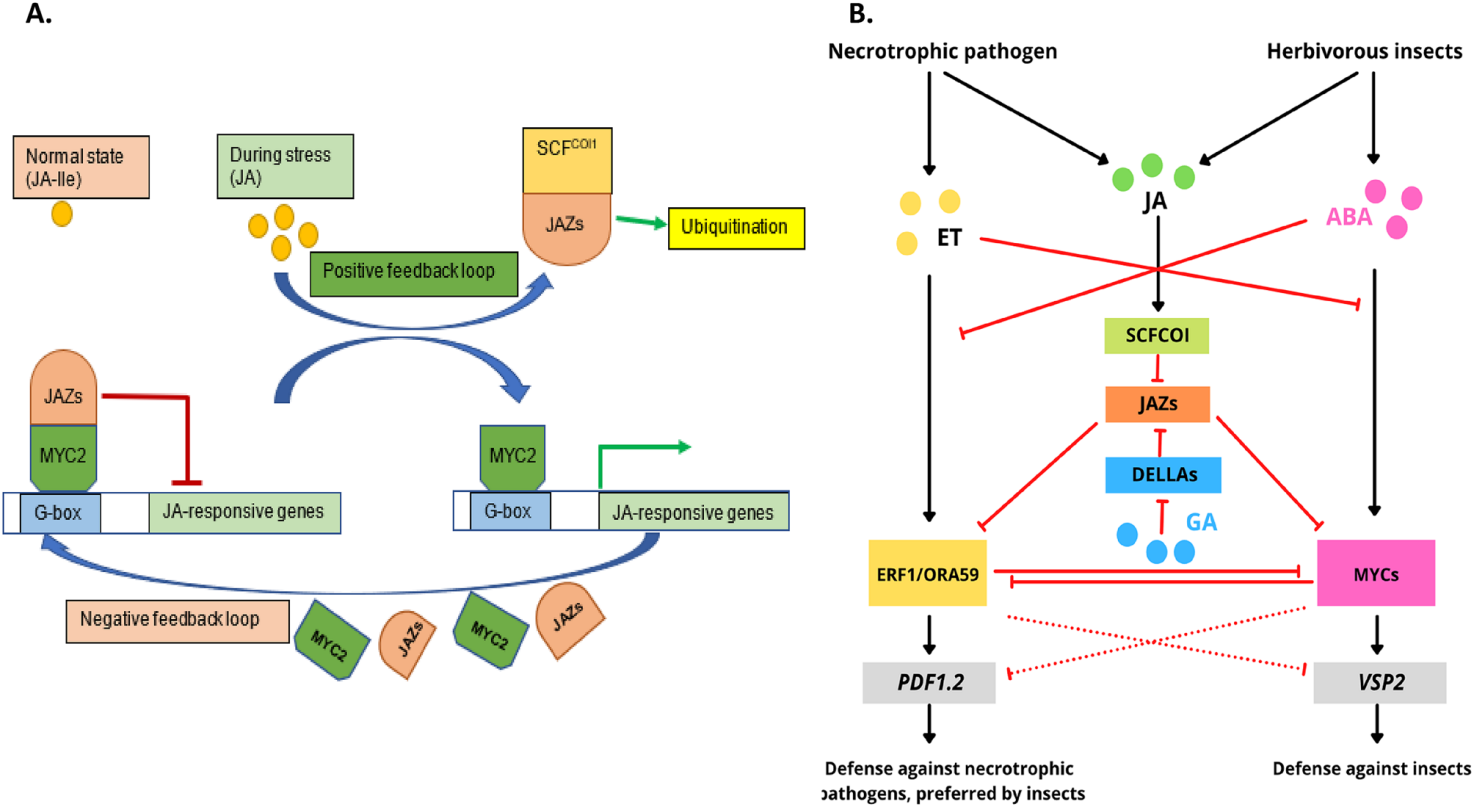
Proposed model for JA perception via COI1-JAZ co-receptor complex (**A**) and crosstalk between phytohormones in the defense signaling pathway (**B**) (adapted from Wasternack and Hause^2^; Pieterse et al.^13^). (**A**) During stress and wounding JAZ are recruited by the SCF^COI1^ and MYC2 are released thus activating JA-defense genes (positive feedback loop). The newly synthesized JAZ genes during defense response binds to the MYC2 thus repressing the JA-defense genes (negative feedback loop). (**B**) Herbivorous insects induce JA- and ABA-dependent signaling pathways while necrotrophic pathogens induce JA- and ET-dependent signaling pathways. Solid lines are established interactions, dashed lines are hypothesized interactions, arrows indicate positive effects, and red inhibition lines represent negative effects. JA, Jasmonic acid; *JAZ*, *JASMONATE ZIM DOMAIN*; SCF^COI1^, SCF-type E3 ubiquitin ligase; *MYC2*, bHLHzip transcription factor MYC2; *ERF1, Ethylene Response Factor 1*; *ORA59, octadecanoid-responsive Arabidopsis AP2/ERF-domain protein 59*; *VSP2, VEGETATIVE STORAGE PROTEIN2*; *PDF1.2, PLANT DEFENSIN1.2.*

Jasmonates (JAs), a group of jasmonic acid and its bioactive derivatives, offer a critical role in plant resilience during biotic and abiotic stresses. JAs play a central role in activating plant responses to herbivory, pathogens invasion, UV radiation and ozone stress^1–4^. In addition, JAs also play a role in development processes like flower initiation and plant morphogenesis^5–7^. In general, JAs response promotes defense and reproduction by reducing growth processes like photosynthesis and cell division^1,8^. Such contrasting functions suggest a broader role of JAs in balancing defense and growth to optimize plant fitness^9^. JA signaling pathway is a complex process and consists of three distinct processes: biosynthesis, JA signal transduction and downstream gene activation. During JA signal transduction, SCF-type E3 ubiquitin ligase (SCF^COI1^) receptor complex*, Jasmonate-ZIM-domain* (*JAZ*) and bHLH (basic helix-loop-helix) transcription factor MYC2 interact with each other to repress or derepress JA-responsive gene expression^9–11^ (Fig. 1A). Besides that, JAZ also plays a central role in the crosstalk between phytohormones signaling cascades of JA, abscisic acid (ABA), ethylene (ET) and gibberellins (GA) in response to stress^12,13^ (Fig. 1B). During herbivory, JA and ABA act synergistically on the expression of the MYC branch which results in the activation of defense-responsive genes^14,15^. In summary, JAZ plays a crucial role in the JA signaling pathway and are central mediators of the signal transduction pathways to activate defense genes.

The JAZ protein family belongs to the TIFY superfamily, which also includes three other subfamilies: ZIM-like (ZML), TIFY and PEAPOD (PPD) families^16,17^. All of the sub-families contain TIFY domains, with one or two different additional domains. The JAZ protein family consists of two highly conserved functional domains, TIFY (also called ZIM) and Jas (also called CCT_2)^18^. The TIFY domain (PF06200) usually consists of 28 amino acids with a consensus sequence “TIF[F/Y]XG”^16^, at the N-terminal of the JAZ protein sequences. Similarly, Jas domain (PF09425) consists of “SLX_2_FX_2_KRX_2_RX_5_PY” at the C-terminal of the JAZ protein sequence^18^. Both highly conserved domains of JAZ play irreplaceable roles in the JA signaling pathway. The TIFY domain mediates the interaction between JAZ proteins and the NINJA-TPL complex and both jointly inhibit the JA signaling^19^. The Jas domains mediate the interaction of *JAZ* with other genes, such as *COI1 *and* MYC,* resulting in the inhibition of transcriptional activities^20–22^. In addition, a previous study indicated that a lack of Jas domain in JAZ reduced the sensitivity of plant resistance to herbivory^9^.

JAZ proteins were first reported in 2007^18,23^ and several studies afterward have well-characterized their functions in *Arabidopsis thaliana*. In *A. thaliana* there are 12 *JAZ* genes^9^. During mechanical wounding and *Spodoptera exigua* herbivory, 11 of the 12 Arabidopsis *JAZ* (*AtJAZ*) were induced significantly^9,24,25^. Among them *AtJAZ1, AtJAZ2, AtJAZ5, AtJAZ6, AtJAZ9, AtJAZ10 *and *AtJAZ12* showed a relatively higher expression in *S. exigua* damaged leaves^9^. This event shows the negative feedback loop in which newly synthesized JAZ can repress the activity of MYC2 thus repressing the defense genes^18,23^ (Fig. 1A). Guo et al.^26^ reported that an Arabidopsis mutant (defective in ten *AtJAZ* genes) exhibited robust resistance to insect herbivores and fungal pathogens at expense of slow vegetative growth and poor reproductive performance. This observation suggests the positive feedback loop since a decrease in JAZ amplifies the plant capacity to release the MYC2 and thus expressing the defense genes^9^ (Fig. 1A). The increase of JAZ in the negative feedback loop is to trade-off growth and defense because overactivation of JA defense response leads to elevated carbon starvation, near loss of seed production and lethality in extreme conditions^26^. Chung et al.^9^ reported that wound-induced synthesis of JAs triggers the degradation of JAZ and subsequent expression of *JAZ* gene response in both positive and negative feedback loops. Taken together, JAZ proteins play an important role during biotic stress responses in plants by regulating defense to balance growth.

Sorghum [*Sorghum bicolor* (L.) Moench] is an important cereal crop in the world for the food, feed and biofuel industry. It ranks fifth in terms of both production and area planted among cereal crops in the world. Sorghum production was declining in years of 2015/2016 and 2016/2017 by 21.6% and 19.5%, respectively^27^. The loss in production is attributed to abiotic and biotic factors, among them, sugarcane aphid (SCA) (*Melanaphis sacchari*) is one of them. SCA outbreak in sorghum occurred in Texas in 2013 and after that it has seriously threatened the sorghum production in more than 20 US states^28^. SCA, a phloem-feeding insect, can attack sorghum at all developmental stages resulting in a significant yield loss and reduced quality of grain^29^. Further, SCA produces honeydew that ultimately reduces photosynthetic area, reduces the seed set and hinders the harvesting process^30,31^. All these have significantly increased the management cost for sorghum growers, yet genetic and molecular mechanisms behind sorghum plant responses to SCA are still unclear. For instance, to develop a resistant variety against SCA, identification of defense-genes and -pathways are crucial.

*JAZ* is one of the important defense gene families and it has been identified and functionally characterized in various plant species, including *A. thalina*^9^, *Malus domesticus*^32^, *Camellia sinensis*^33^, *Brassica oleracea*^34^*, Petunia*^35^ and *Triticum aestivum*^36^. Yet, identification and functional characterization of *JAZ* has not been reported in sorghum to date. In our study, we performed comparative genomics, domain-scan and phylogenetic analysis to identify and see the evolutionary relationship of the sorghum *JAZ* gene family. A detailed in silico analysis of promoter regions was conducted to identify the stress-related cis-acting elements and transcription factors (TFs). In addition, two Recombinant Inbred lines (RILs), resistant (RIL 521) and susceptible (RIL 609) to SCA, were used for expression profiling. The expression profiling of the sorghum *JAZ* gene in response to SCA infestation and four phytohormone treatments (JA, GA, ET, ABA) were investigated in both RILs. Our analysis provides insight into the biological function of sorghum *JAZ* genes and elucidates its possible role in phytohormone crosstalk and restraining a defense to balance the growth.

## Materials and methods

### Sequence acquisition of *JAZ* genes

To identify *JAZ* genes in sorghum, a single two-step approach was used. First, all the Arabidopsis JAZ proteins were used to perform a BLAST search among the whole genome sequences of sorghum. The protein sequences identified from the searches with E-value < 10^−5^ were pooled, and redundant sequences (identical sequences from multiple matching) were removed. Secondly, the acquired sorghum proteins were checked for the presence of TIFY and Jas domains using the InterProScan program (http://www.ebi.ac.uk/Tools/InterProScan/) and the Pfam database (http://pfam.xfam.org/) was used to confirm their presence^37^. The identified sorghum *JAZ* genomic sequence and coding sequence (CDS) were retrieved from Phytozome (https://phytozome-next.jgi.doe.gov/). These sequences were analyzed for exon/intron organization using the gene structure display (GSDS) tool^38^. The physical and chemical parameters of each sorghum JAZ (SbJAZ) protein were predicted using the ExPasy program (https://web.expasy.org/protparam/) and subcellular localization were predicted through WoLF PSORT online program (https://wolfpsort.hgc.jp/). The chromosomal position of *SbJAZ* genes obtained from the Phytozome were used to create the genetic map using chromoMap package in R^39^.

### Phylogenetic analysis of plant JAZ

To establish the evolutionary relationship of SbJAZ proteins, a total of 106 JAZ proteins from seven representative plants, including a bryophyte (*Physcomitrella patens*), lycopodiophyte (*Selaginella moellendorffii*), monocots (*O. sativa* and *S. bicolor*) and eudicots (*A. thaliana*, *Brassica oleracea* and *Camelia sinensis*), were used for the phylogenetic analysis. The protein sequences of these seven plants were downloaded from NCBI and Phytozome^33^. A phylogenetic tree was constructed using the maximum likelihood method with Poisson correction model using 1000 bootstrap values^40,41^ in the MEGAX program^42^. To better understand the evolutionary relations, sorghum JAZ genes were compared to those homologs in rice for their chromosomal location and genomic structure.

### Sequence analysis and identification of conserved motifs

Structural motif annotation for all 18 JAZ proteins were performed using the MEME program (http://meme-suite.org/tools/meme). We also manually checked the alignments of all SbJAZ proteins to identify the conserved sequences and pivotal amino acids. The online tool weblogo^43^ was used to generate the sequence logos of conserved regions present in SbJAZ proteins. Furthermore, the two conserved domains, TIFY and Jas domains, of sorghum were aligned with the domain of *O. sativa* and *A. thaliana* to observe the cross-species conservation of these domains.

### Gene duplication and calculation of dN/dS ratio

The two types of gene duplication, segmental and tandem duplication are well known. Segmental gene pairs consist of 90% sequence similarity, while tandem consists of five or fewer genes within the 100 kb region^44,45^. After the identification of duplicated gene pairs, CDS sequences of each JAZ genes were used to calculate the synonymous (dS) and nonsynonymous (dN) rate of substitution using MEGA X according to Han and Luthe^46^. First, the coding sequences were aligned by Clustal W, then dN and dS was estimated using Nei-Gojbori substitution model^47^. Next, to calculate the p-value of the selection, codon-based *Z*-test was performed on each pair of sequences. With the p-value (< 0.05 are considered significant at the 5% level) of each pair of sequences, neutral evolution (dN = dS), positive selection (dN > dS) or purifying selection (dN < dS) were tested on each pair of sequences.

### Prediction of cis‑regulatory elements and transcription factor networks

About 2000 bp promoter sequences upstream to ATG for all *SbJAZ* genes obtained from the Phytozome were analyzed using the PlantCARE promoter analysis tool^48^. Then, the identified various cis-elements were used to construct the heatmap through the gplots package in R software^49^. Further, transcription factor network prediction was performed as described by Wang et al.^50^ with minor modifications. The promoter sequence used earlier was submitted to the Plant Transcriptional Regulatory Map (PTRM) (http://planttfdb.gao-lab.org/prediction.php) to predict the transcription factors. These transcription factors were subjected to Cytoscape 3.8.2 to visualize the transcription factor regulatory network^51^. Furthermore, KEGG and GO analyses were conducted using the PTRM and DAVID Bioinformatics resources 6.8 (https://david.ncifcrf.gov/).

### Sorghum plant growth and treatments

Sorghum seeds from parental lines (Tx2783 and BTx623) and the recombinant inbred lines (RILs), RIL 521 and RIL 609 developed from the parents, were grown in greenhouse at constant temperature (28 ± 2 °C) and 60% relative humidity under constant photoperiod of 14 h-light/10 h-dark. The seeds of the two parental lines (Tx2783 and BTx623) and Tx7000 were available to public use, which were originally obtained from the Germplasm Resources Information Network (GRIN, https://www.ars-grin.gov/) in the U.S. and the recombinant inbred lines (RIL 521 and RIL 609) were produced in our lab. All plant materials used in this study comply with local and national guidelines. Sugarcane aphid colonies were cultured on susceptible sorghum line (Tx7000). Sorghum seedlings of the four lines of 10–12 days old (2–3 leaf stage) were infested with 20 adults of apterous sugarcane aphid to the adaxial surface of the first true leaf. Each infested plant and the control plants (not infested with aphids) were covered, respectively, with a transparent cylindrical cage with nylon mesh on the top. To evaluate the differential responses to aphid in the four lines (Tx2783, BTx623, RIL 521 and RIL 609), the aphids on each plant were counted and recorded at 1, 3, 6 and 9 days post infestation (dpi) from ten independent plants of each infested lines. For phytohormones treatment, two RILs were sprayed separately with MeJA (100 μM), ABA (100 μM), ET (100 μM), SA (100 μM) and sterile distilled water (control) until runoff. Samples, first two true leaves and stem of the seedlings, were harvested from each treatment (MeJA, ABA, ET, GA and control) at 6 h after spraying. For SCA infestation analysis, samples (first two true leaves and stem below the second leaf) were collected from the two RILs infested with sugarcane aphids and without (control) at 0, 6-, 24- and 48-h post infestation (hpi). Each sample harvested had three biological replicates for each time point and were frozen immediately in liquid nitrogen and stored at − 80 °C. The control samples were collected at each time point to eliminate the circadian rhythm effect on gene expression.

### RNA extraction and quantitative real-time PCR analysis

Quantitative RT-PCR (qRT-PCR) was used to estimate the relative expression of sorghum *JAZ* in response to aphid infestation and phytohormone treatment. A Trizol reagent (Invitrogen, Carlsbad, CA) was used to extract the total RNA from each sample and then it was treated with DNase (Turbo DNA-free kit, Thermo Fisher Scientific, Waltham, MA). A total of 2.5 μg of RNA was reverse-transcribed using the GoScript reverse transcriptase kit (Promega, Madison, WI) and the resulted cDNA was diluted four-fold before using for the qRT-PCR reaction. Primers were designed using the IDT DNA program (https://www.idtdna.com/PrimerQuest/Home/Index), which are listed in Table S1. A sorghum *α-Tubulin* gene (Sobic.001G107200) was used as the internal control as described previously, and this gene showed a stable expression throughout various treatments in sorghum^52^. qRT-PCR was performed on a Bio-Rad iCycler thermal cycler (Bio-Rad Laboratories, Inc., Hercules, CA, USA) using the iTaq™ universal SYBR^®^ green supermix (Bio-Rad Laboratories, Inc.). The qRT-PCR reaction was performed in a volume of 10 μl, containing 1 μl of cDNA, 0.4 μl (10 μM) each of the reverse and forward primers, 5 μl of SYBR green master mix and 3.2 μl of ddH2O under the following conditions: one cycle at 95 °C for 3 m, 40 cycles at 95 °C for 10 s and 55 °C for 30 s, followed by one cycle each of one min at 95 °C and 55 °C. The final melting curve was 81 cycles at 55 °C for 30 s.

### Statistical analysis

The relative expression level of each gene was calculated using the 2^−ΔΔCt^ method^53^ and the data presented are the averages of three biological and two technical replicates. For the aphid count data, ANOVA and Tukey test was used to estimate the significant difference. For expression analysis during phytohormones and SCA infestation, Student’s *t*-test was used to estimate the significant difference (*P < 0.05, **P < 0.01 and ***P < 0.001).

## Results

### Identification of the *JAZ* gene family in sorghum

To identify the *SbJAZ* gene family a single two-step approach was used, homology search and proteome scan for the presence of “TIFY” and “Jas” domains. From the first approach, 26 *SbJAZ* genes were identified. The second approach narrowed the *SbJAZ* to eighteen (Table 1). The decrease in the *JAZ* genes is due to the missing of one of the domains, or the addition of extra domains (VEFS and GATA) (Table S2). The true JAZ sequence should contain both TIFY and Jas domains^34,35^.

**Table 1:** Structural features of the *JAZ* genes in sorghum.

Name	Gene ID	Chromosome	Exon_count	Orientation	Location coordinates	Genomic (bp)	CDS (bp)	ORF (aa)	PI	MW (kDa)	GRAVY
SbJAZ1	Sobic.001G259300	1	3	Forward	31035454..31036798	1345	681	226	9.06	24.29	−0.538
SbJAZ2	Sobic.001G259400	1	1	Forward	31205564..31206743	1180	507	168	9.92	17.34	−0.089
SbJAZ3	Sobic.001G259600	1	1	Forward	31327155..31328316	1162	528	175	9.29	17.97	0.006
SbJAZ4	Sobic.001G259700	1	1	Forward	31345064..31346119	1056	666	221	9.81	22.72	−0.316
SbJAZ5	Sobic.001G259900	1	1	Forward	31385111..31385784	674	561	186	8.42	19.58	−0.152
SbJAZ6	Sobic.001G276900	1	2	Forward	54019381..54020155	775	675	224	8.32	24.09	−0.462
SbJAZ7	Sobic.001G343900	1	5	Forward	63205787..63208276	2490	687	228	8.98	24.05	−0.445
SbJAZ8	Sobic.001G482600	1	1	Reverse	75415876..75416893	1018	585	194	8.78	19.94	−0.153
SbJAZ9	Sobic.001G482700	1	3	Reverse	75423136..75425280	2145	606	201	7.70	21.14	−0.372
SbJAZ10	Sobic.001G482800	1	1	Forward	75429377..75432763	3387	543	180	9.33	18.87	−0.274
SbJAZ11	Sobic.002G036100	2	2	Reverse	3392335..3395495	3161	729	242	9.33	24.82	−0.517
SbJAZ12	Sobic.002G196200	2	7	Reverse	58508296..58511272	2977	1227	408	9.83	42.80	−0.338
SbJAZ13	Sobic.002G214800	2	5	Reverse	60694460..60696521	2062	666	221	9.30	23.38	−0.476
SbJAZ14	Sobic.002G374100	2	5	Forward	73193557..73195985	2429	708	235	9.25	24.92	−0.37
SbJAZ15	Sobic.003G410300	3	4	Forward	71777501..71782156	4656	303	100	9.16	10.60	−0.559
SbJAZ16	Sobic.006G056400	6	5	Forward	40020080..40027257	7178	537	178	9.81	18.84	−0.341
SbJAZ17	Sobic.006G244800	6	5	Forward	58463788..58467690	3903	660	219	7.71	23.16	−0.47
SbJAZ18	Sobic.007G132700	7	7	Reverse	55342328..55345325	2998	1287	428	9.55	45.30	−0.34

JAZ genes having only TIFY or CCT domain are listed in Supplementary Table S2.

*CDS* coding DNA sequence, *PI* isoelectric point, *MW* molecular weight.

Table 1 summarizes the characteristics of the *SbJAZ* gene family, including Gene IDs and gene features. In this study, gene name was assigned from *JAZ1* to *JAZ18* according to their positions in the chromosome. At the genomic level *SbJAZ* sequences ranges from 674 to 7178 bp and coding DNA sequences (CDS) range from 303 to 1287 bp. The length of SbJAZ proteins varied between 100 and 428 aa and the predicted molecular weights range from 10.60 to 45.30 kDa. The PI ranges from 7.7 to 9.92, indicating that all the sorghum JAZ proteins were basic. The grand average of hydropathicity (GRAVY) values of all SbJAZ proteins, except SbJAZ3, is less than 0, implying that they all are hydrophilic proteins. Further detailed information about the instability index, aliphatic index, and subcellular localization of all the SbJAZ proteins are listed in Table S3.

### Phylogenetic and structural analysis of sorghum *JAZ* genes

**Figure 2:**
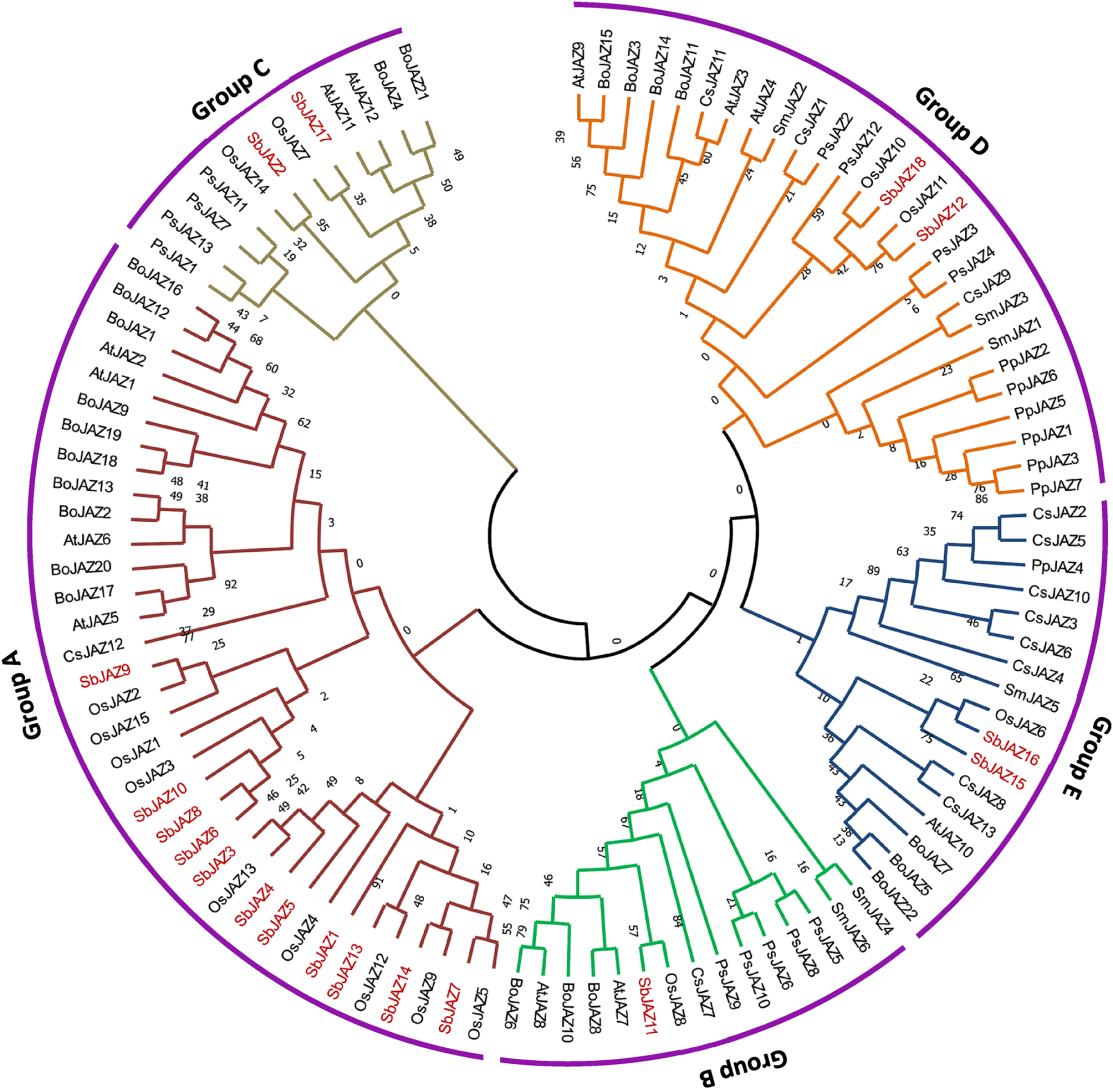
Phylogenetic analysis of the JAZ gene family. The circular tree represents the evolutionary relationship between the sorghum JAZ protein family with those in bryophyte, lycopodiophyte, gymnosperms, monocots and eudicots. The tree was generated by MEGAX using the Maximum Likelihood method based on the Poisson correction model using 1000 bootstrap values^40,41^. The analysis involved 106 amino acid sequences and they are divided into five classes. Species abbreviations used for phylogeny are as follows. At: *Arabidopsis thaliana*, Bo: *Brassicae oleracea*; Cs: *Camellia sinensis*; Os: *Oryza sativa*; Pp: *Physcomitrella patens*; Ps*: Picea sitchensis;* Sb: *Sorghum bicolor* and Sm: *Selaginella moellendorffii.*

To determine the evolutionary relationship of sorghum JAZ, a total of 106 JAZ proteins from eight representative plant species, including a bryophyte (*Physcomitrella patens,* 7), lycopodiophyte (*Selaginella moellendorffii,* 6), gymnosperms (*Picea sitchensis,* 13), monocots (*O. sativa* (15) and *S. bicolor*) and eudicots (*A. thaliana* (12), *B. oleracea* (22) and *C. sinensis* (13) were used for the phylogenetic analysis^33^ (Table S4). The JAZ proteins were divided into five groups (Groups A–E), which is in line with previous classifications (Fig. 2)^35^. Amongst five groups, Group A was the largest one, with three sub-groups followed by Group D and Group E with two sub-groups in each. The sorghum JAZ protein family were distributed in all the groups: 11 in group A (SbJAZ1, SbJAZ3-10, SbJAZ13-14), two in each of the group C (SbJAZ2 and SbJAZ17), D (SabJAZ12 and SbJAZ18) and E (SbJAZ15-16), and the remaining one in group B (SbJAZ11).

**Figure 3:**
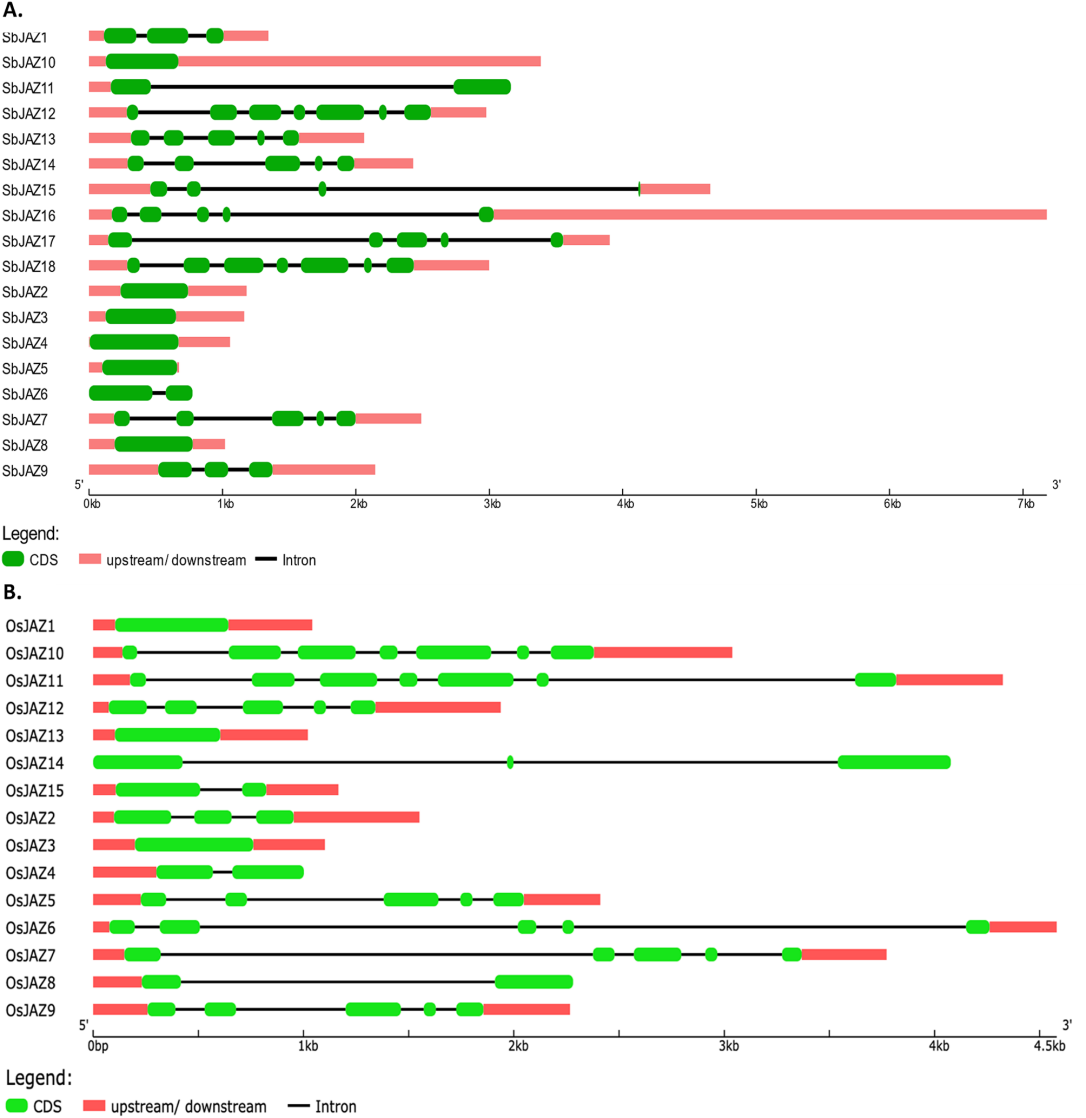
The schematic genomic structure for each *SbJAZ* gene (**A**) and *OsJAZ* (**B**) was generated using gene structure display server (GSDS). The size of coding DNA sequence (CDS), introns and untranslated regions are proportional to their sequence lengths.

**Figure 4:**
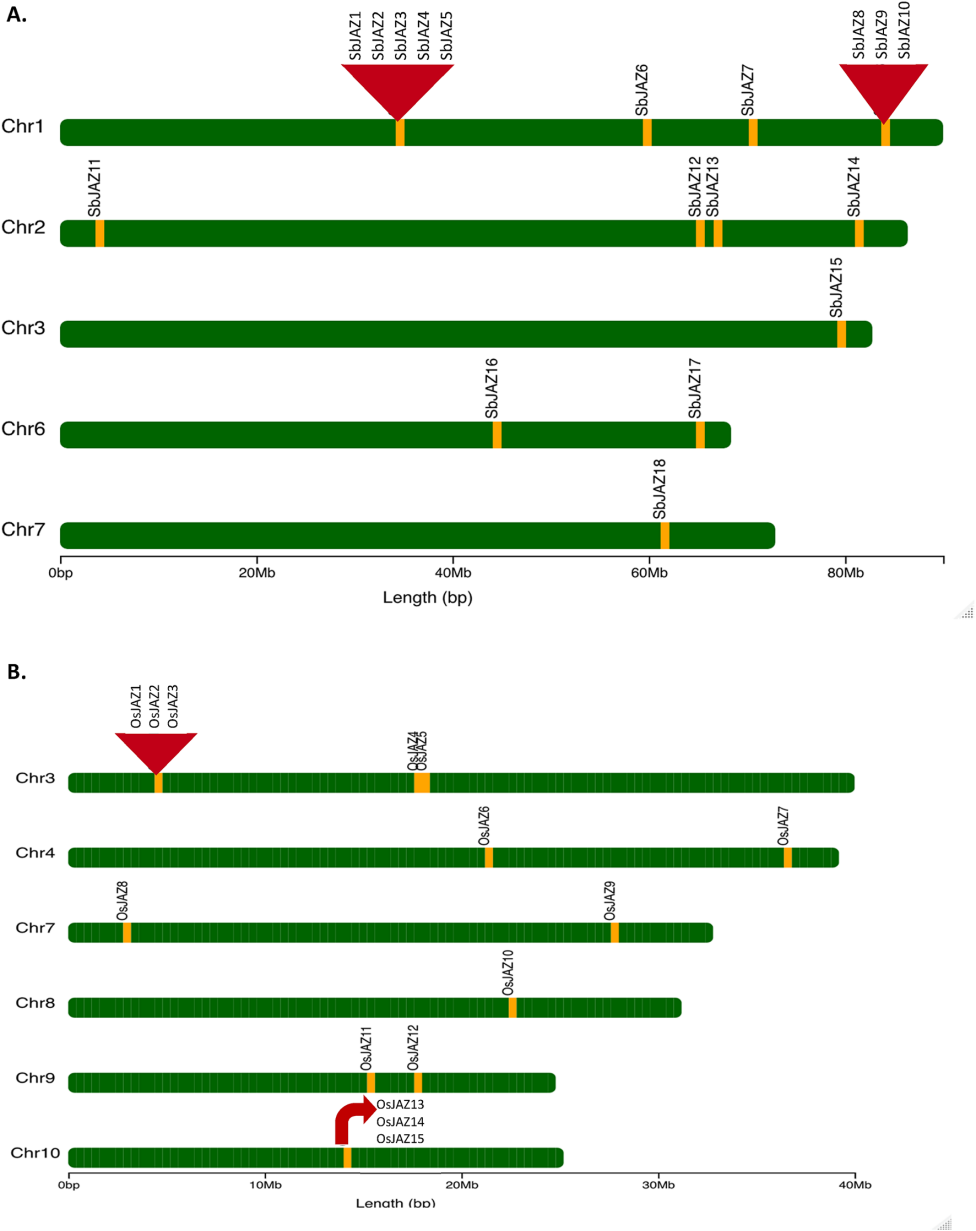
Genome mapping of the 18 *SbJAZ* genes on *S. bicolor* chromosomes (**A**) and *15 OsJAZ* genes on *O. sativa* chromosomes (**B**). Gene locations and chromosome size can be measured using the scale on the bottom of the figure.

To study the structural diversity of the *SbJAZ* genes, intron/exon organization of coding sequence of each gene were constructed (Fig. 3). The 18 *SbJAZ* genes are mapped to five chromosomes (Chr 1, 2, 3, 6 and 7) out of the ten sorghum chromosomes (Fig. 4A), with ten residing on Chr 1, four on Chr 2, two on Chr 6 and one on each of Chr 3 and 7. All sorghum *JAZs* are consisted of 5′ and 3′ untranslated regions (UTRs) except *SbJAZ6* and *SbJAZ11*. Similarly, all sorghum *JAZ* have exon number ranging from 1–7 and intron numbers ranging from 0–6 (Fig. 3). The divergence in gene structure could also support the clustering of SbJAZ proteins in the phylogenetic tree (Fig. 2)^54^. The cross-species comparison with the 15 *OsJAZs* showed that *O. sativa* has only two genes with single exons in comparison to six in *SbJAZs* (Fig. 3). In terms of *JAZs* gene distribution, *O. sativa* has more uniform distribution of JAZs in chromosomes with highest in Chr3 (five *JAZ* genes), compared to ten *SbJAZ* genes on Chr 1 of sorghum (Fig. 4).

### Conserved motif analysis in sorghum JAZ proteins

**Figure 5:**
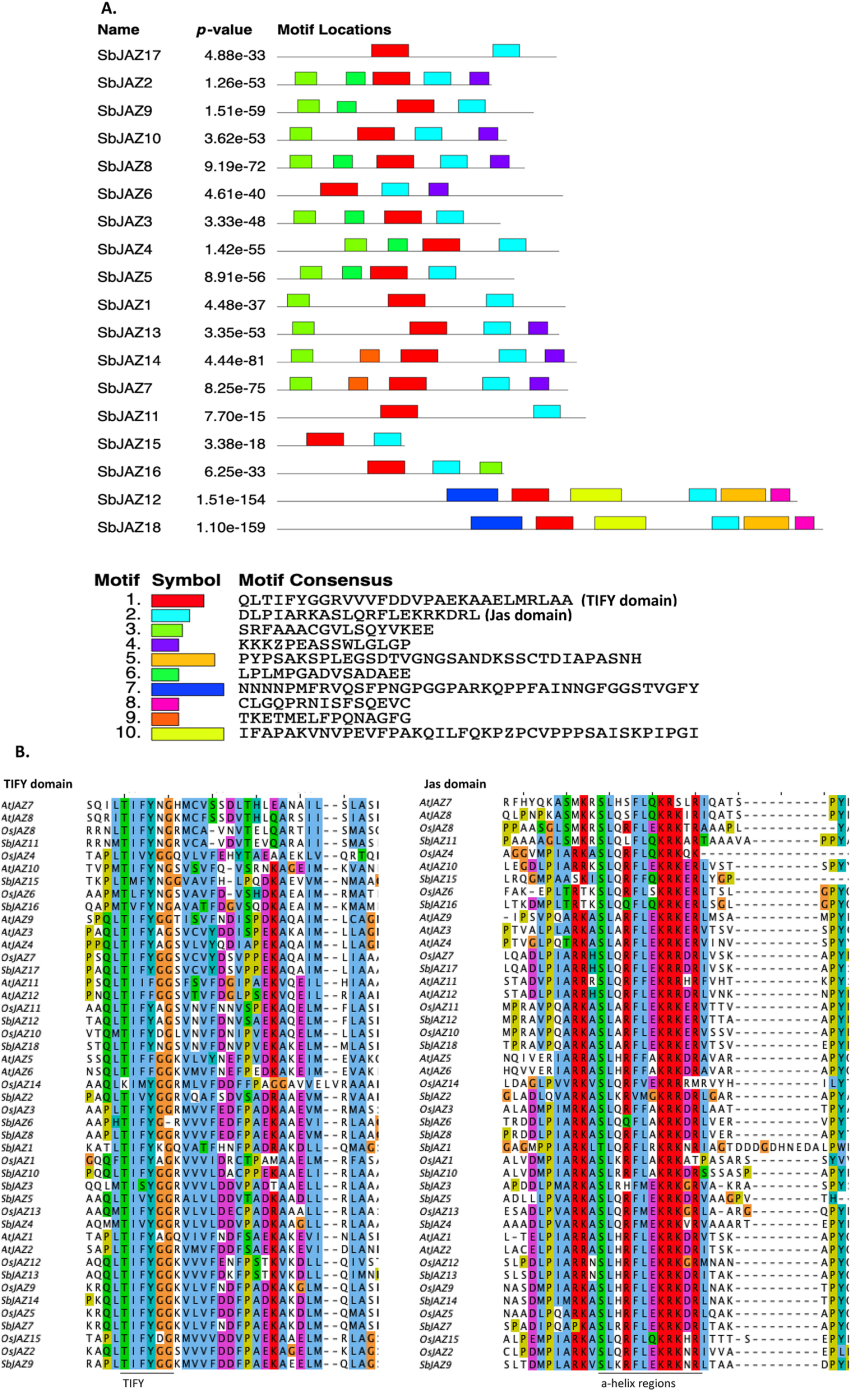
(**A**) Schematic representation of the conserved motifs in sorghum JAZ proteins. Conserved motifs and their alignment among JAZ proteins was elucidated by using MEME tool. Most of the proteins possess similar domains revealing their conserveness. Each colored box represents a motif in the protein, with the consensus motif sequence below in the index. (**B**) Cross-species comparison of TIFY and Jas domains revealed the sequence conservation of these domains.

A total of ten distinct motifs in the SbJAZ protein family were identified through MEME suite (Fig. 5A). Among them two motifs, TIFY (red) and Jas domain (sky blue), were conserved in all the SbJAZ proteins (Fig. 5A). These two motifs and their locations vary among the sub-groups, but are present in all members of the JAZ family. To identify the conserved residues in those two domains, all 18 SbJAZ protein sequences were subjected to weblogo. Here, the TIFY domain has core “TI[F/V]YXG” motif and Jas domain has “SLX_2_FX_2_KRX_2_RX_7_PY”. Both of these domains are the defining trait of JAZ proteins, and are well conserved in sorghum JAZ. The sequence logo for TIFY and Jas domains generated from SbJAZ members are similar to the HMM profile generated from Pfam database (Fig. S1) which suggests these residues are conserved throughout the species. Further cross-species alignment of TIFY and Jas domain showed that these domains are well conserved in the monocots and dicots with some minor changes in few genes (Fig. 5B). The Jas domain motif of some of the proteins (*OsJAZ4, SbJAZ15, SbJAZ1, SbJAZ5, OsJAZ2, OsJAZ14, OsJAZ8*) lack the PY motif at the end. Previous studies have showed that the PY motif is not required for the ligand-dependent COI1-JAZ interaction, but the a-helix region of Jas domain is the one to bind the COI1 and JA hormone, which is conserved throughout the species^55^. Similarly, TIFY domain is highly conserved in all of the genes which acts as a repressor for JA signaling^19^.

### Gene duplication and estimation of dN/dS values

In general, there are two major events in gene duplication, tandem or segmental, leading to an increase in number of genes in a family. The results of phylogenetic analysis showed that none of the *SbJAZ* gene pairs has 90% similarity between each other to be defined as segmental repeats^56^. Based on chromosomal location, six gene pairs were identified in Chr 1 as tandem duplication (Table 2). These pairs might have originated from two separate self-duplication events in Chr 1, first among *SbJAZ3, SbJAZ4 *and* SbJAZ5* and second among *SbJAZ8, SbJAZ9* and *SbJAZ10*. To further explore the driving force of *SbJAZ* gene evolution, dN/dS ratio and *Z*-test were calculated. The ratio of 1 indicates neutral selection, ratio > 1 indicates positive selection and ratio < 1 indicates purifying selection. From the Table 2, The dN/dS ratios of all six gene pairs were below 1, but the p-value showed significant purifying selection for *SbJAZ8-9* gene pairs only, so other gene pairs occurred as the neutral selection.

**Table 2:** Analysis of tandem duplication events of sorghum JAZ gene pairs.

Gene pairs	dN	dS	dN/dS	p-value
SbJAZ3-SbJAZ4	0.434	0.465	0.934	0.282
SbJAZ3-SbJAZ5	0.381	0.397	0.960	0.383
SbJAZ4-SbJAZ5	0.344	0.414	0.830	0.082
SbJAZ8-SbJAZ9	0.377	0.474	0.795	0.027*
SbJAZ8-SbJAZ10	0.341	0.354	0.961	0.392
SbJAZ9-SbJAZ10	0.413	0.485	0.852	0.088

*dN* nonsynonymous substitutions rate, *dS* synonymous substitutions rate. To be considered purifying selection dN/ < 1 and p-value for the *Z*-test should be below 0.05 (*P < 0.05).

### Identification of *cis*-elements within the *SbJAZ* genes

**Figure 6:**
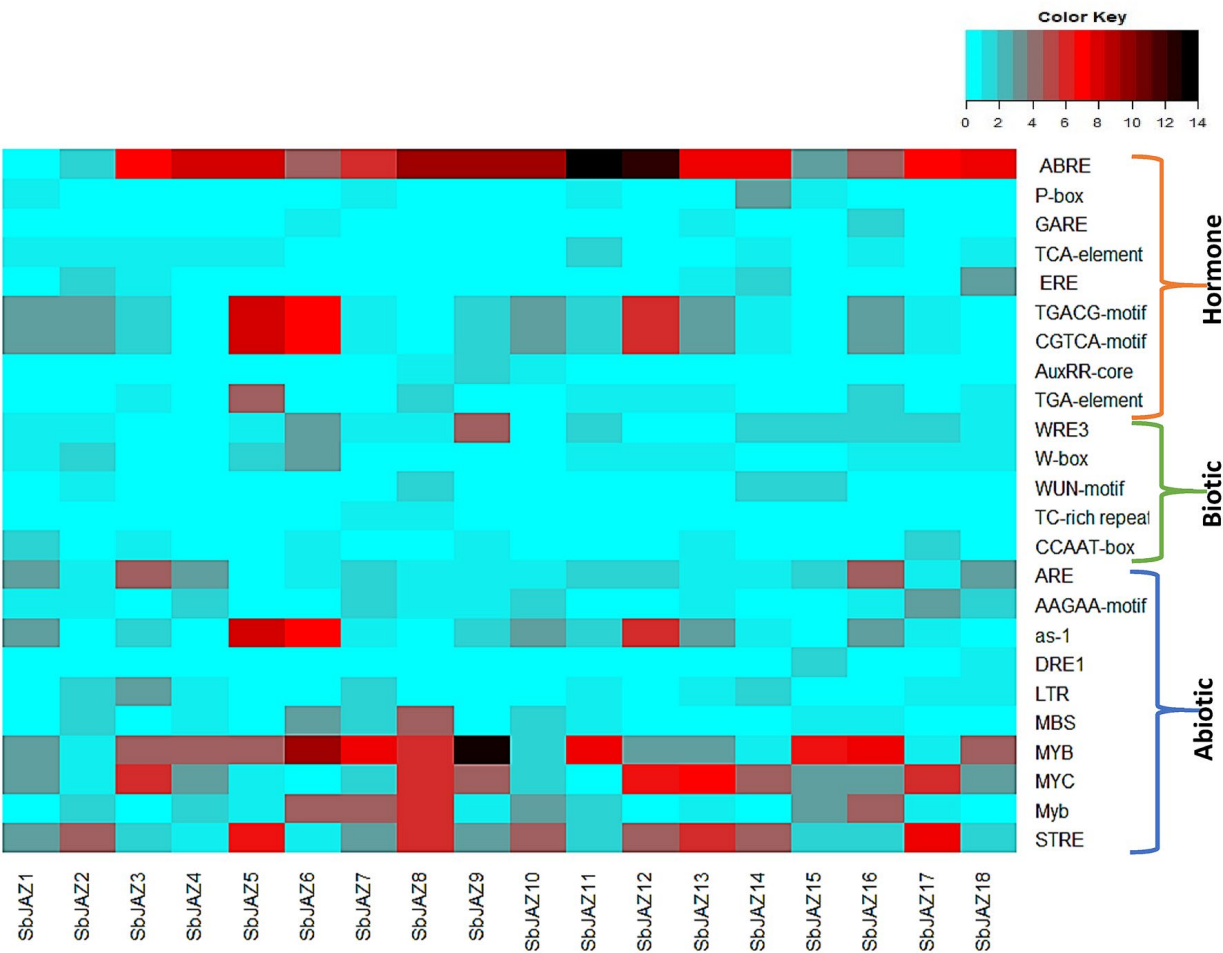
Heatmap representing the distribution of *cis*-elements in the promoter regions (2000 bp upstream) of sorghum *JAZ* genes. The scale (color key) represents the number of cis-elements in each *SbJAZ* genes (1: sky blue, 7: red, 14: black).

The *cis*-elements present upstream of a gene play a critical role in regulatory function involved in plant stress response and growth^57^. An in silico analysis of the 2000 bp upstream of the *SbJAZ* genes were conducted through the Plant Care database. The summary of the *cis*-elements was plotted in heatmap (Fig. 6) and according to their functions, the *cis*-elements were grouped into categories such as abiotic, biotic and hormone responsive. Almost all the *SbJAZ* genes possess plant hormone-responsive elements, ABA (ABRE), GA (P-BOX and GARE), SA (TCA-element), AUX (TGA-element, AuxRR-core) and JA (TGACG-motif and CGTXA-motif) (Table S5). Among them, ABA and JA responsive motifs were present abundantly in the *SbJAZ* genes. These results suggest that the *SbJAZ* family may be associated with the complex hormone regulatory network. Similarly, abiotic stress-responsive elements, such as STRE, Myb, MYC, MYB, MBS and as-1 were also present abundantly. Among them, the two *cis*-elements MBS and Myb were the drought-inducible motif. Meanwhile, some biotic-responsive elements, like WRE3, W-box, WUN and CCAAT-box, were present in a small number in some *SbJAZ* genes (Fig. 6 and Table S5). These motifs WUN and CCAAT-box are stress and defense inducible elements. Notably among 18 sorghum *JAZ* genes promoters, *SbJAZ5, SbJAZ6, SbJAZ8, SbJAZ13, SbJAZ16* and *SbJAZ17* are rich in *cis*-elements.

### Identification of transcription factor regulatory network of the *SbJAZ* genes

**Figure 7:**
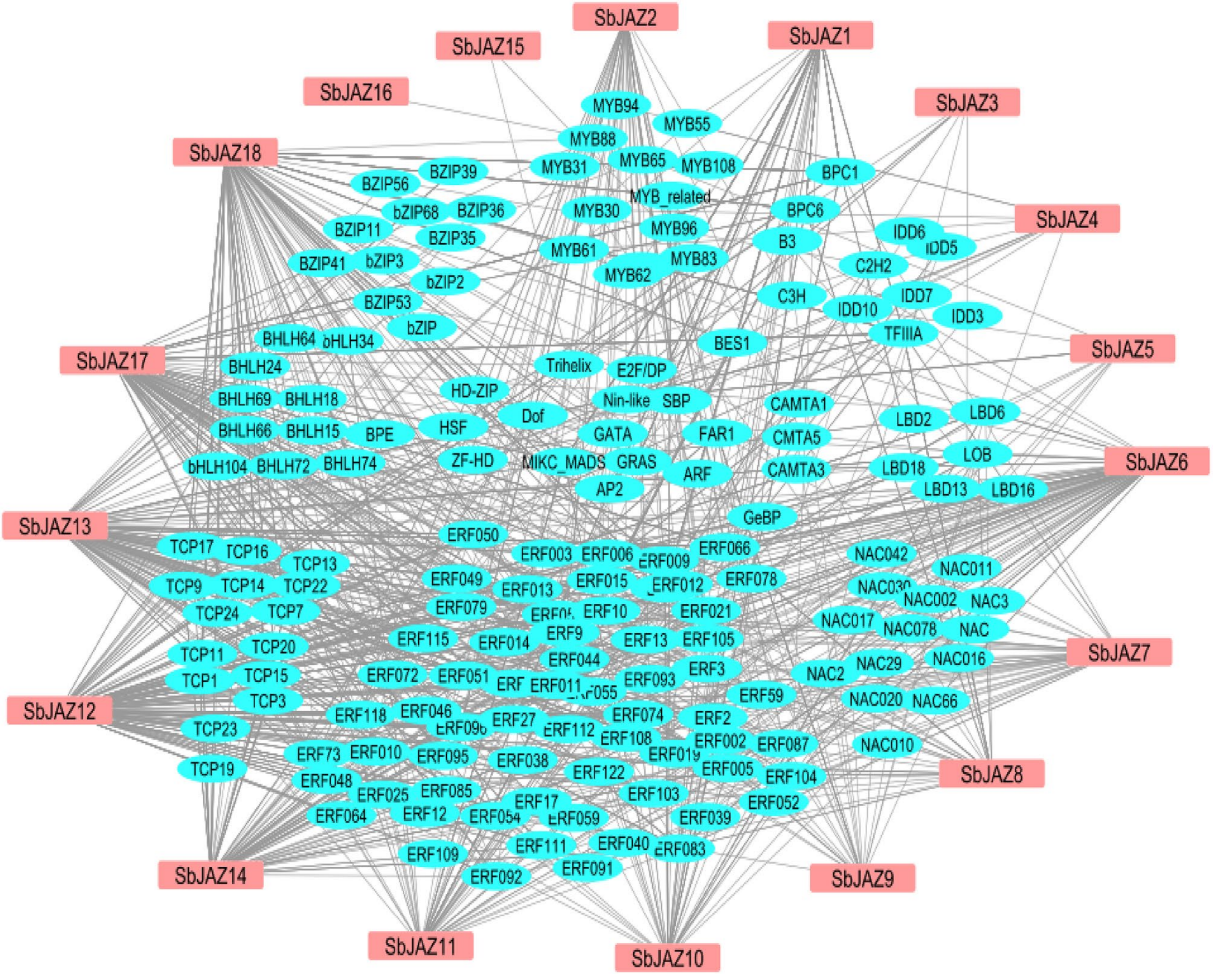
The putative transcription factor regulatory network of the *SbJAZ* genes. The network was constructed using the PTRM tool and Cystoscope.

To explore the potential transcriptional network of *SbJAZ genes*, 2000 bp upstream sequences from the 18 *SbJAZ* genes were analyzed through the PTRM website. The results showed 211 transcription factors (TFs) participated in the regulation of sorghum *JAZ* genes (Table S6). These TFs were used to construct a transcription factor regulatory network (Fig. 7). The TFs network shows that the *SbJAZ* family were possibly regulated by the following seven transcription factor families, ERF, TCP, bHLH, MYB, C2H2, LBD and NAC. Among them, members of the ERF family were the most abundant, followed by TCP, NAC and bHLH.

Furthermore, to understand the biological functions of the 18 *SbJAZ* genes, GO and KEGG enrichment analyses were conducted. The results (Table 3) showed almost all the *SbJAZ* genes except *SbJAZ8* were related to the plant responses to defense (GO:0031347), wounding (GO:0009611) and the jasmonic acid signaling pathway (GO:2000022) (Table 3). Moreover, these genes were also enriched with transcription corepressor activity (GO:0003714) and negative regulation of nucleic acid transcription (GO:1903507). Interestingly, the KEGG pathway analysis indicated that all the *SbJAZ* genes were regulated only in the JA signal transduction pathway (map04075) (Fig. S2).

**Table 3:** Gene ontology of the sorghum *JAZ* genes.

Gene ontology	Process	Functions associated with GO	Present in all SbJAZ genes except
GO:1903507	BP	Negative regulation of nucleic acid-templated transcription	*SbJAZ8*
GO:2000022	BP	Regulation of jasmonic acid mediated signaling pathway	*SbJAZ8*
GO:0031347	BP	Regulation of defense response	*SbJAZ8*
GO:0009611	BP	Response to wounding	*SbJAZ8*
GO:0005634	CC	Nucleus	*SbJAZ8*
GO:0003714	MF	Transcription corepressor activity	*SbJAZ8*

*BP* biological process, *CC* cellular component, *MF* molecular function.

### Differential response between resistant and susceptible lines

The two parental lines (Tx2783 and BTx623) and two RILs (RIL 521, SCA-resistant and RIL 609 SCA-susceptible) infested with SCA showed differential responses to aphid infestation. The Tx2783 is a resistant and BTx623 a susceptible genotype against SCA infestation^58,59^. Following aphid infestation, Tx2783 and RIL 521 lines showed adverse effect on aphid development and fecundity in comparison to the susceptible genotype. The number of aphids per plant was counted and based on that the rate of aphid regeneration was significantly reduced on Tx2783 and RIL 521 lines from early dpi (1- and 3-dpi) to late (6- and 9-dpi) (Fig. S3 and Table S7). Similarly, less SCA damage was noted on Tx2783 and RIL 521 lines in compared to the susceptible genotype.

### Expression patterns of *SbJAZ* genes after exogenous phytohormone treatments

**Figure 8:**
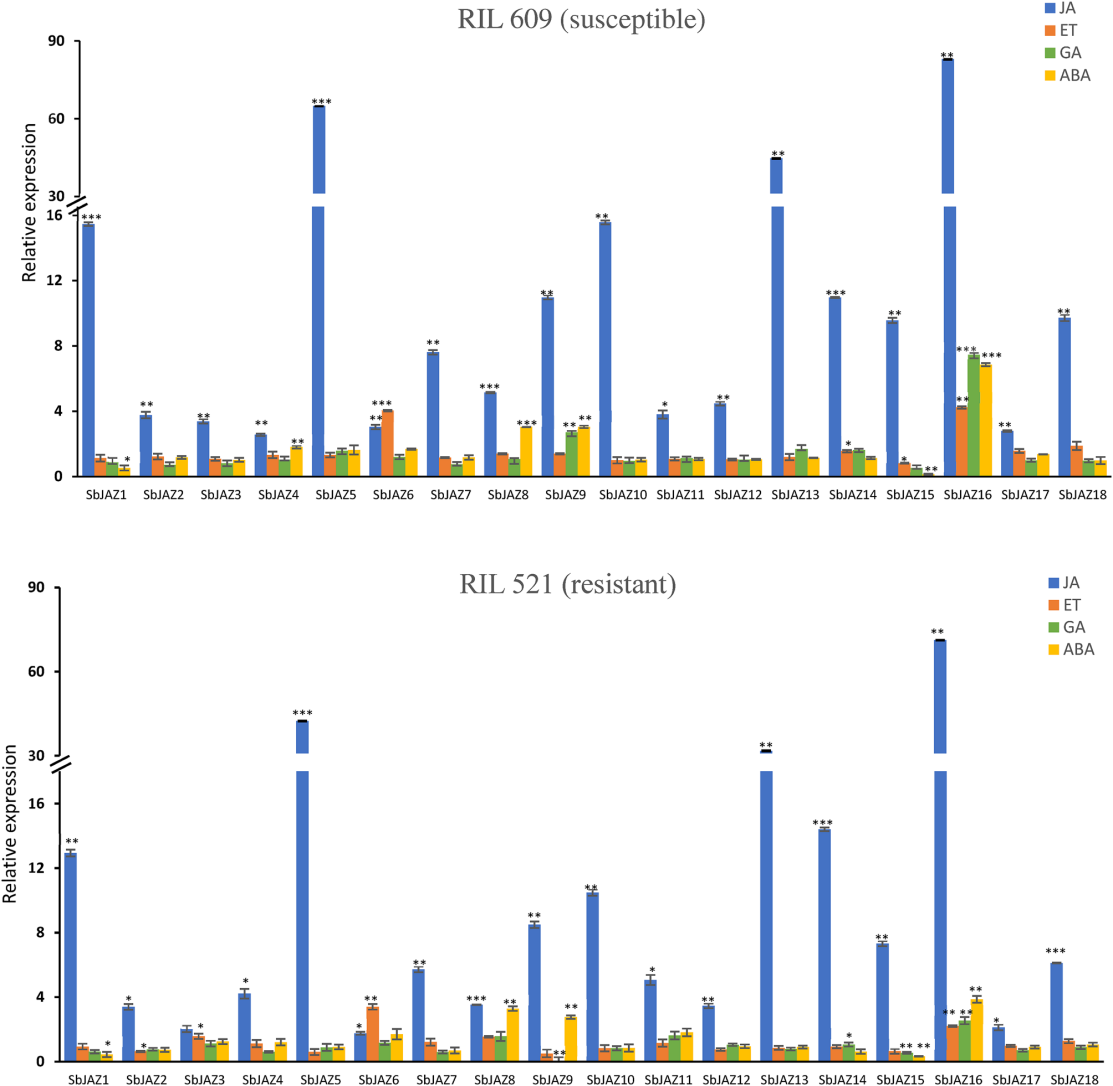
Expression pattern of 18 *SbJAZ* genes in response to the treatments with phytohormones (JA, ET, GA, and ABA) in resistant (RIL 521) and susceptible (RIL 609) RILs to SCA. qRT-PCR was used to determine the relative expression of each *SbJAZ* gene, and the relative expression was estimated using the 2^−ΔΔCt^ method. Error bars in each bar represent the ± standard error (n = 3) and asterisks indicate significant differences between the control and phytohormone treated samples, *P < 0.05, **P < 0.01, ***P < 0.001. The bars without asterisk, are non-significant (P > 0.05).

Here, qRT-PCR was conducted to evaluate the response of sorghum *JAZ* genes in both resistant (RIL 521) and susceptible (RIL 609) RILs after phytohormones (JA, ET, GA and ABA) treatments. Following treatment with MeJA, almost all the *JAZ* genes in both RILs were upregulated (Fig. 8). Among 18 *SbJAZ* genes, three of them were upregulated more than *30-fold, SbJAZ5, SbJAZ13 *and* SbJAZ16*, four of them were upregulated more than eightfold, *SbJAZ1, SbJAZ9, SBJAZ10 *and* SbJAZ14* while the other eleven *SbJAZ* genes were upregulated between two to eight-fold (Fig. 8). In contrast, other three phytohormones, treatments with ET, GA and ABA didn’t show consistent upregulation in expression of sorghum JAZ genes as that induced by MeJA. After ABA treatments, *SbJAZ8*, *SbJAZ9 *and* SbJAZ16* showed upregulation of more than three-fold while *SbJAZ15* showed downregulation. Similarly, after ET treatment, *SbJAZ6* and *SbJAZ16* showed more than four-fold upregulation in both RILs. Among all *SbJAZ* genes, SbJAZ16 showed concurrent upregulation in all four phytohormone treatments.

### Expression patterns of *SbJAZ* genes after SCA infestation

**Figure 9:**
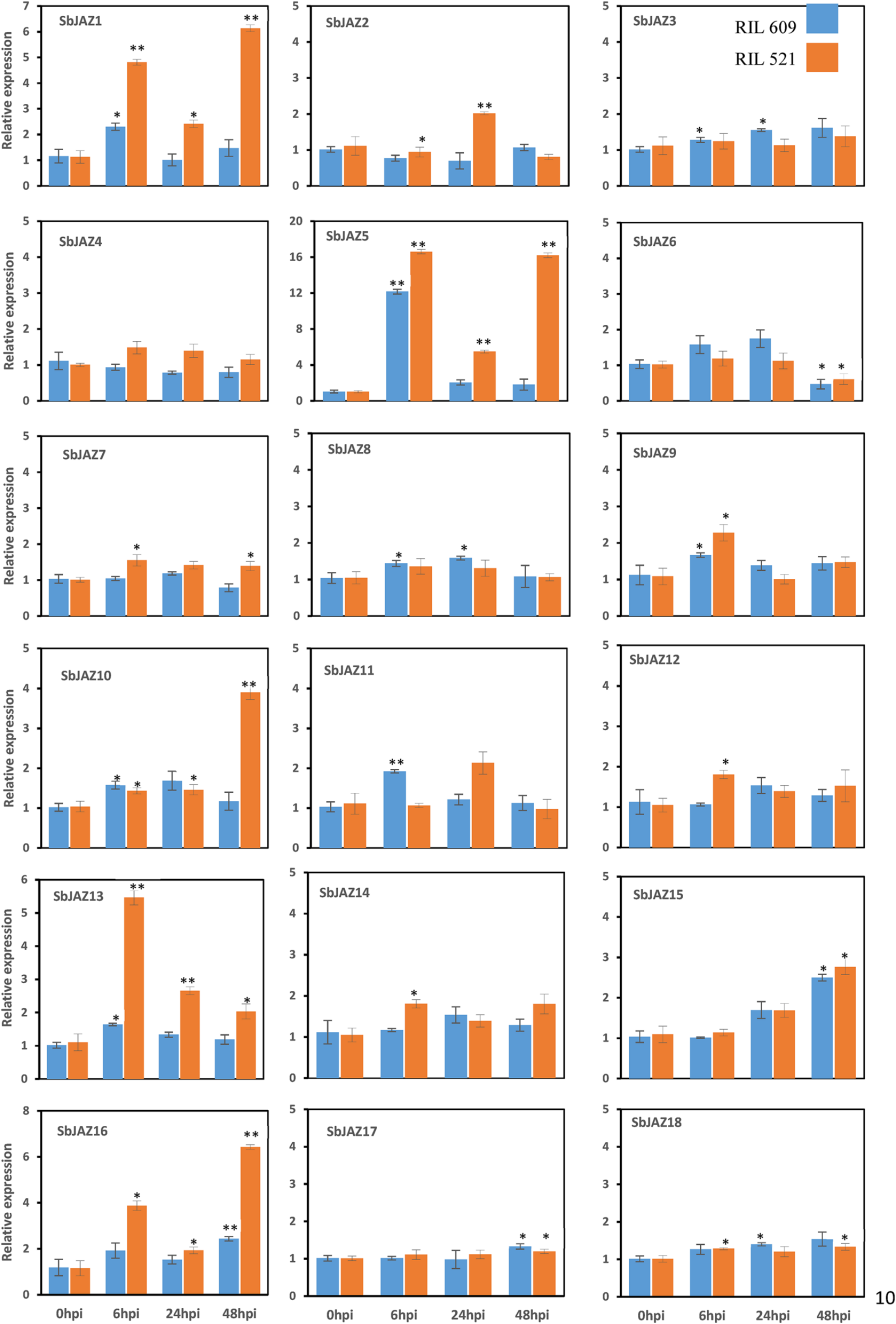
Expression pattern of 18 *SbJAZ* genes in response to sugarcane aphid infestation in in resistant (RIL 521) and susceptible (RIL 609) RILs to SCA. qRT-PCR was used to determine the relative expression of each *SbJAZ* gene, and the relative expression was estimated using the 2^−ΔΔCt^ method. Error bars in each bar represent the ± standard error (n = 3) and asterisks indicate significant differences between the control and phytohormone treated samples, *P < 0.05, **P < 0.01. The bars without asterisk, are non-significant (P > 0.05).

The qRT-PCR was conducted to observe the expression patterns of sorghum *JAZ* genes in both RILs after SCA infestation. The expression pattern was observed in all four-time points, 0, 6, 24 and 48 hpi. Most of the sorghum *JAZ* genes, except *SbJAZ3, SbJAZ6, SbJAZ8, SbJAZ17* and *SbJAZ18*, at different time points (except 0 hpi) showed significantly higher expression in the RIL 521 in comparison to RIL 609 (Fig. 9). Among 18 *SbJAZ* genes, *SbJAZ1, SbJAZ5, SbJAZ13* and *SbJAZ16* showed more than five-fold upregulation in one or more of the time points in RIL 521. *SbJAZ5* showed the highest upregulation among these four *SbJAZ*, around 16-fold in both 6 hpi and 48 hpi following SCA infestation and only five-fold in 24 hpi. Similar pattern of up and downregulation was observed in *SbJAZ1* and *SbJAZ16*. These up and downregulation of *JAZ* genes at different time points suggest there are continuous interactions going on between the plant and aphid to dominate each other. Three other genes, *SbJAZ9, SbJAZ10, SbJAZ15,* showed 2 to fivefold upregulation in resistant lines at different time points. Interestingly, *SbJAZ6* showed downregulation in both the RILs during SCA infestation at 2 dpi.

## Discussion

JAZ proteins participate in stress response and play a central mediator role in the phytohormone signaling pathways to activate defense genes. Besides that, JAZ proteins balance growth and defense response by avoiding the detrimental effects of hyper-immunity caused by JA defense^26^. To date, 12 *JAZ* genes have been reported and studied in *A. thaliana*, 18 in *M. domesticus*^32^, 13 in *C. sinensis*^36^ and 14 in *T. aestivum*^36^. However, the study of the sorghum *JAZ* gene family and its functional role has not been reported. In this study, we carried out genome-wide analysis and expression profiling of the *SbJAZ* gene family and identified its functional role in plant development and defense.

### Structural characteristics of sorghum *JAZ* genes

All *JAZ* genes contain of TIFY domain at N-terminal and Jas domains at C-terminal, which are characteristic features of *JAZ*. The TIFY domains mediate the interactions with NINJA, whereas Jas domain mediates the interactions with COI and MYC. The sorghum genome possesses a total of 18 *JAZ* genes that contain both domains (Table 1). The sorghum *JAZ* number is similar to *Hevea brasiliensis* (18)^60^, *M. domestica* (18), *O. sativa* (15) and *B. oleracea* (22), but higher than in* P. patens* (7) and *S. moellendorfii* (6). The higher number of *JAZ* family members indicates the expansion event in seed plants^33^. Interestingly, all 18 JAZ were basic in nature with PI value ranging from 7.70–9.92, suggesting that individual SbJAZ proteins may require different ionic strength for their optimal activity^44^. The sorghum *JAZ* genes consist of introns and exons ranging from 0–6 and 1–7, respectively. Similar number of introns (0–7) were noted in *JAZ* gene family of other plants such as rice and wheat^61^. Most of the dicots have at least one intron in *JAZ* genes, but the introns were lacking in monocots: rice, wheat, maize and sorghum^36,62,63^. The reason behind lacking introns in monocots might reflect the evolutionary difference from dicots^35^. The study suggests that fewer introns within a gene family will make plants more responsive to environmental stresses^64,65^. Therefore, sorghum *JAZ*, *SbJAZ1-6* and *SbJAZ8-10* may respond quickly to environmental stresses, for which further verification is needed.

### Evolutionary relationship of the *JAZ* protein family

In this study, a total of 106 JAZ proteins from eight representative plants including a bryophyte lycopodiophyte, gymnosperms, dicots and monocots were used for phylogenetic analysis. These eight plant species belong to four plant terrestrial groups, which include the earliest land plants (bryophyte) to recently evolved C_4_ plants (sorghum). These JAZ family members are supposed to be originated from terrestrial plants because they were not found in algal genomes^33^. All the 106 JAZ proteins were clustered into 5 distinct groups (A–E) (Fig. 2). Within these groups, we found some lineage-specific JAZ sub-families. In group D, there was a separate clade only for bryophyte, and a clade for gymnosperms in Group B and C. Similarly, Group A showed exclusive clusters of monocots and dicots, which suggest that these genes might have evolved after the separation of monocots and dicots^66^. The grass family has accumulated many JAZ genes though duplication and transposon insertion^46^. The phylogeny and cross-species comparison with *O. sativa* (15 *JAZs*) revealed that a higher number in sorghum (18 *JAZs*) may be related to this extra duplication event in *SbJAZ1-5*. The phylogenetic tree (Fig. 2) forms a clade of *SbJAZ2* with *OsJAZ14* and *SbJAZ3-5* with *OsJAZ13,* which suggests they are orthologs. Phylogenetic tree also reveals that the extra duplication events are *SbJAZ4-5*, which is further supported by their genomic structure as three *SbJAZ3-5* had one exon which is similar to one exon of *OsJAZ13* (Fig. 3).

Gene duplication event undergoes through either neutral, purifying and positive selection. In the *SbJAZ* gene family, evidence of neutral and purifying selection was obtained based on dN/dS and codon-based Z test (Table 2). Among six gene-pairs, five of them showed a neutral selection, indicating that the duplicated genes in these groups were not in strong selection pressure and many mutations evolved and remain in the population. These gene members could have developed more precise or new functions during the evolution^46^. One reason for most of the sorghum gene pairs showing neutral selection probably is that most of these pairs belong to tandem duplications, which tends to have a larger dN/dS ratio^67^. Interestingly, one of the gene pair (*SbJAZ8-9*) showed a purifying selection, indicating that they are evolving slowly at the protein level and suggests the stability of SbJAZ protein family during evolutionary process. Similar results of both purifying and neutral selection were reported in maize JAZs^46^.

### Putative functions of the sorghum *JAZ* genes

The TFs are central regulators of the gene expression as it modulates the gene expression by binding to local and distal *cis*-acting elements of neighboring gene under different stresses^68^. The TFs regulatory network shows the sorghum *JAZ* genes are rich in ERF, TCP, NAC bHLH and MYB families (Fig. 7). All five TFs are induced during both biotic and abiotic stresses. Among them, NAC is one of the largest plant-specific TFs and acts via an ABA-dependent as well as independent pathway and play a vital role in both abiotic and biotic stress^69^. A previous study reported that NAC TFs was induced in sorghum during greenbug infestation^70^. Similarly, MYB TFs of sorghum and maize were also induced during fungal pathogen^71^ and leaf blight pathogen ingress^72^. The ERFs TFs play a vital role in ABA-independent pathways and are involved in both biotic and abiotic stress (drought and salt stress)^68^. The *VaERF* in grapes showed higher expression in response to *Botrytis cinerea* infection^73^. The other TFs, bHLH are induced during drought, osmotic stress and salt stress^74^. The sorghum *JAZ* genes rich with these TFs suggest the potential role of these genes being activated under biotic and abiotic stresses.

### Expression profiling of sorghum *JAZ* during phytohormone treatment

Phytohormone signaling pathways are involved during plant-pest/pathogen interaction and several studies showed the repressor role of JAZ in defense signaling and in crosstalk between JA and other hormones^12^. For the expression analysis, RILs were used because one can phenotype multiple individuals from each RIL while reducing the individual, environmental and measurement variation^75^. The phytohormone treatment results (Fig. 8) showed that most of the sorghum *JAZ* genes were significantly upregulated in both the RIL 521 and RIL 609. During MeJA treatment, most of the *SbJAZ* genes were significantly upregulated, and in ABA treatments, *SbJAZ8, SbJAZ9* and *SbJAZ16* showed significant upregulation. Similarly, *SbJAZ6* and *SbJAZ16* were induced by the ET treatment and *SbJAZ16* also showed significant upregulation during GA treatment. Among them, *SbJAZ16* was upregulated during all the four phytohormone treatments. A similar kind of upregulation was reported in *B. oleracea*^34^ and* C. sinensis*^76^ during different phytohormone treatments. *B. oleracea* treated with MeJA showed significantly higher upregulation in eight *BoJAZ* genes out of 18, while when treated with ET, only two of them showed significant upregulation^34^. By combining the *cis*-elements results of this upregulated genes (Fig. 6), we found *SbJAZ8* and *SbJAZ9* were rich in ABA-responsive elements and *SbJAZ16* were rich in the JA-, ABA- and GA-responsive elements. In contrast, both the *SbJAZ6* and *SbJAZ16* lacked the EA-responsive elements. Interestingly, all *SbJAZ* promoter regions have JA-responsive elements except *SbJAZ4, SbJAZ8, SbJAZ15* and *SbJAZ18*. By combining the results of *cis*-elements and expression analysis we speculated that two *SbJAZ* genes (*SbJAZ9* and *SbJAZ16*) were involved in JA-ABA crosstalk and *SbJAZ16* in JA-GA crosstalk. Similar research conducted by Wang et al.^36^ in *JAZ* genes of *T. aestivum* indicated the crosstalk between JA-GA and JA-ABA with expression profiling of phytohormones treatment. They also reported the upregulation in a *JAZ* gene although they lack the respective *cis*-elements. In contrast, some of the *SbJAZ* genes contain abundant ABA (*SbJAZ11-12*) and MeJA (*SbJAZ6* and *SbJAZ12*) responsive cis elements but showed low gene expression during their respective hormone treatment^33^. Similar low expression pattern was reported for *CsJAZ* genes during ABA and MeJA treatment though they were rich in the respective cis elements. One possible reason is that gene expression is not only determined by the presence of relevant cis elements but also by other physiological processes^76^. Similarly, besides promoter regions there are also several non-coding sequences in the front of the gene, which can either induce or suppress transcription of the gene. The JA-GA crosstalk is to promote plant growth and defense. Similarly, JA-ABA crosstalk acts synergistically to activate the defense-responsive genes against herbivory (Fig. 1B). Besides that, JA-ABA crosstalk also provides tolerance against salinity and drought and low temperature^36^. These results indicate that the *SbJAZ* genes can respond to JA, ABA and GA signaling, suggesting their role in the crosstalk between these phytohormones to promote defense gene activation^34^.

In both *A. thaliana* and *T. aestivum,* eight *JAZ* genes out of 12 and 21, respectively, were responsive to MeJA^9,24,36^. Similarly, in *C. sinensis* eight *JAZ* genes were significantly upregulated when treated with MeJA, among them four increased more than 30-fold^76^. The upregulation of *JAZ* genes induced by MeJA is controlled by short transcriptional cascades^77^. The upregulation of *SbJAZ* in both susceptible and resistant RILs during MeJA treatment implies the existence of a negative feedback loop to minimize the detrimental effects during hyper-immunity. In a negative feedback (Fig. 1A) loop, the newly synthesized *JAZ* repressor dampens the JA defense response by inhibiting the activity of MYC2 TFs^9^. Plant immune responses mediated by JA-Ile are metabolically costly and often linked to stunted growth^26^. Similarly, suppressed JAZ proteins further exacerbate the growth, nearly abolish the seed production and cause tissue death under extreme conditions^26^. The upregulation of *SbJAZ* genes in both RILs suggests its functions in minimizing the effects of hyper-immunity, promoting the growth and maintaining reproductive success.

### Expression and functions of *SbJAZ* genes in response to SCA infestation

*JAZ* are key repressors in the JA signal transduction pathway and play a crucial role in stress-related defense and restraining a defense to balance growth. The SCA infestation expression analysis (Fig. 9) indicated that out of 18 *SbJAZ* genes, four of them (*SbJAZ1, SbJAZ5, SbJAZ13 *and* SbJAZ16*) were significantly upregulated in RIL 521 (resistant) in comparison to un-infested and RIL 609 (susceptible). This significantly higher expression of *SbJAZ* genes in resistant lines further supports the involvement of the JA pathway in host plant defense against SCA. Previous research showed significant upregulation of the lipoxygenase (*LOX*) genes in sorghum resistant lines during SCA infestation as the *LOX* gene is a marker gene for JA biosynthesis pathway^78^. All three *SbJAZ* genes (*SbJAZ1, SbJAZ5, SbJAZ13*) are in Group A clustered with four orthologous genes in *A. thaliana* (*JAZ1, JAZ2, JAZ5, JAZ6*) and *B. oleracea* (*JAZ12, JAZ18, JAZ19, JAZ20*), respectively, in a phylogenetic tree (Fig. 2). The remaining *SbJAZ16* is clustered with *AtJAZ10* and *BoJAZ7* in Group E. All the five Arabidopsis *JAZ* were induced to high levels following mechanical wounding and *Spodoptera exigua* herbivory^8,9,25^. Similarly, all the five *BoJAZ* genes were significantly induced in resistant lines when infested with *Plasmodiophora brassicae*^34^. The *BoJAZ7* clustered with *SbJAZ16* is also significantly induced by *Xanthomonas campestris* and *Fusarium oxysporum* inoculation^34^. Four *SbJAZ* genes were also significantly increased during MeJA treatment in resistant lines (Fig. 8). Wound-induced expression of *JAZ* genes has been reported in *Populus*^79^, *Solanum lycopersicom*^80^ and Arabidopsis^9^, indicating that this phenomenon is conserved in the plant kingdom. The increased expression of JAZ proteins in resistant sorghum suggests a negative feedback loop (Fig. 1A) where the newly synthesized JAZ repressor dampens the JA response by inhibiting the activity of MYC2 TFs^9^. Overactivation of JA defense leads to carbon starvation, near loss of seed production and plant lethality (under extreme conditions)^26^. Interestingly, *SbJAZ6* showed downregulation in the resistant line during SCA infestation. A similar result was reported in *BoJAZ17* which is close to *SbJAZ6* during *F. oxysporum* inoculation^34^. The inhibition of *SbJAZ6* indicates the positive feedback loop (Fig. 1A) where the decrease in *JAZ* amplifies the plant capacity to release the MYC2 and thus expressing the defense genes^9^. Similar up and downregulation of the *JAZ* genes were noted in *B. juncea* during *P. brassicae* inoculation^81^. Previous research has also suggested the role of JAZ proteins in regulating plant processes that may confer resistance to insect herbivores through production of glucosinolate-based defenses^9,82^. In short, expression of *JAZ* genes helps in the regulation of plant defense, promotes growth and ensures reproductive success by restraining the immune response.

As shown in Fig. 6, the promoter regions of the five sorghum genes (*SbJAZ1, SbJAZ5, SbJAZ6, SbJAZ13 *and* SbJAZ16*) are rich in phytohormone motifs (ABA- and JA-responsive elements) and abiotic stress motifs (MYB, STRE). Among the five genes, *SbJAZ5* consists of more motifs related to phytohormones and abiotic stresses, likely supported by its upregulation during MeJA treatment and SCA infestation in the resistant line. These results imply that these *cis*-elements present upstream of the gene regulates its expression under stresses. In the future, RNAi and CRISPR/Cas9 technology can be used on the potential *SbJAZ* genes from this study to develop a resistant cultivar against SCA. Similarly, yeast two-hybrid assay can be used to verify the functions of the potential *SbJAZ* genes by looking its interaction with MYC2 TFs. The role of *SbJAZ* during SCA infestation can also be validated by measuring the endogenous JA content and secondary metabolites during SCA infestation.

## Conclusion

For the first time, this study has identified and characterized 18 *JAZ* genes from the sorghum genome through bioinformatic analysis and expression profiling. An in silico protein analysis showed the conserved TIFY and Jas domains in all *SbJAZ* genes, implying both the critical structural features and the conserved functions. Simultaneously, in silico analysis of the promoter region of *SbJAZ* genes revealed that the six sorghum *JAZ* genes were rich in *cis*-elements and TFs. These cis-elements and TFs are responsive to a variety of stresses and are verified in other plant species. The *JAZ* genes play an important role in phytohormone crosstalk to activate defense genes and are irreplaceable during wound and stress response. The expression profiling of phytohormone treatments in resistant (RIL 521) and susceptible (RIL 609) RILs to SCA has shown the possible role of *SbJAZ9* in JA-ABA crosstalk and *SbJAZ16* in both JA-ABA and JA-GA crosstalk to regulate certain physiological processes in plants. Notably, during JA treatment and SCA infestation, four *SbJAZ* genes (*SbJAZ1, SbJAZ5, SbJAZ13 *and* SbJAZ16*) showed strong expression in resistant RIL, implying their potential roles in stress response and regulating plant defense to balance the growth. Overall, these findings provide an insight into the important functions of the *JAZ* genes in host plant defense and genetic resources for genetic engineering in sorghum.

## Supplementary Information

Supplementary Information.
