# Supplementary material for: Genome-wide characterization of the sorghum JAZ gene family and their responses to phytohormone treatments and aphid infestation
Source: Sci Rep. 2022 Feb 25;12:3238. doi: 10.1038/s41598-022-07181-9 (PMC8881510; doi:10.1038/s41598-022-07181-9)
Supplement: Supplementary file 1 — Supplementary Information. [file 41598_2022_7181_MOESM1_ESM.pdf]

Genome-wide characterization of the sorghum *JAZ* gene family and their responses to phytohormone treatments and aphid infestation

Kumar Shrestha[1], Yinghua Huang[1,2]✉

[1] Department of Plant Biology, Ecology and Evolution, Oklahoma State University, Stillwater, OK 74078, USA.

[2] United States Department of Agriculture - Agricultural Research Service (USDA-ARS), Plant Science Research Laboratory, Stillwater, OK 74075, USA.

✉ Corresponding author: yinghua.huang@usda.gov

Supplementary tables and figures legends

Figure S1. HMM profile of the TIFY (PF06200) and the Jas (PF09425) domains generated from the Pfam database.

Figure S2. The KEGG pathway of the *SbJAZ* genes. All the *SbJAZ* genes were regulated in the JAZ signal transduction pathway (map04075).

Figure S3. Line graph showing mean numbers of aphids on the four lines at 1, 3 6 and 9 dpi. Error bars represent ± standard errors (n=10). Significant differences were determined by the ANOVA and Tukey test.

Table S1. Primers for the 18 sorghum *JAZ* genes

Table S2. List of genes not considered as true JAZ as they are lack of TIFY, CCT or have extra domains (VEFS or GATA zinc finger)

Table S3. Summary of biochemical properties and subcellular localization of the *SbJAZ* genes

Table S4. The information of *JAZ* genes in different plant species

Table S5. Distribution of cis elements in the promoter regions (2000bp upstream) of the *SbJAZ* genes

Table S6. Transcription factors of the *SbJAZ* genes

Table S7. Aphid count data for four sorghum lines after co-cultivation with SCA

A.

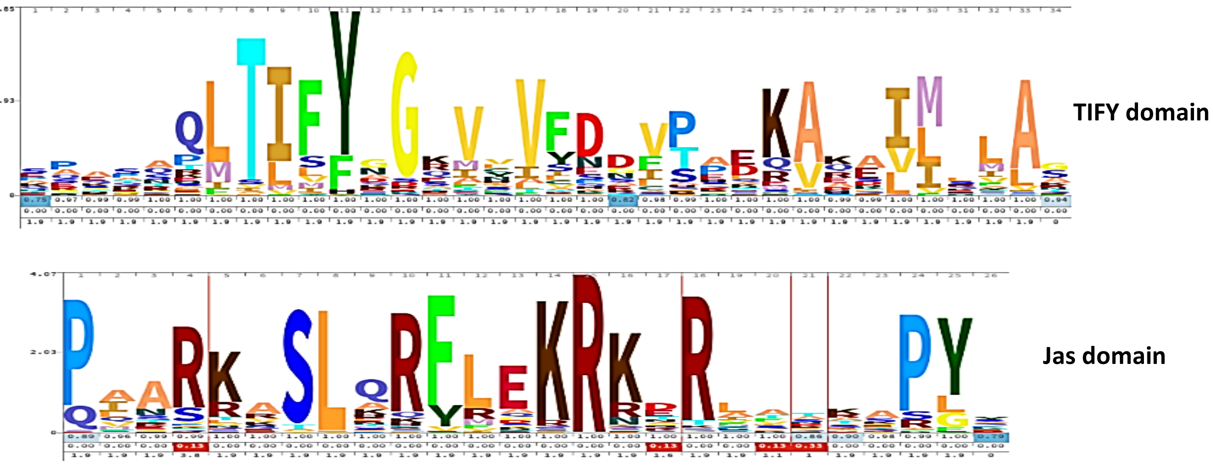

B.

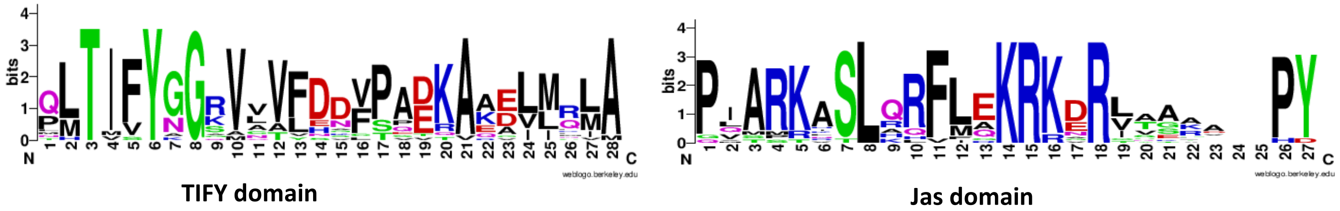

Figure S1. HMM profile of the TIFY (PF06200) and the Jas (PF09425) domains generated from the Pfam database (A) and 18 SbJAZ proteins using Weblogo (B). In each stack y-axis represents the sequence conservation at that position. Each residue height represents the relative distribution frequency of this residue in all the 18 sorghum JAZ motifs.

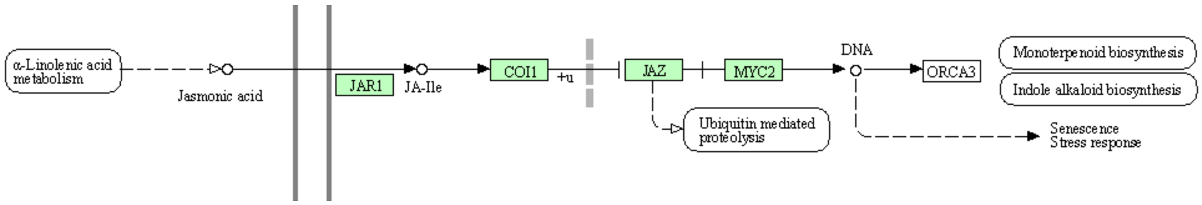

Figure S2. The KEGG pathway of the *SbJAZ* genes. All the *SbJAZ* genes were regulated in the JAZ signal transduction pathway (map04075).

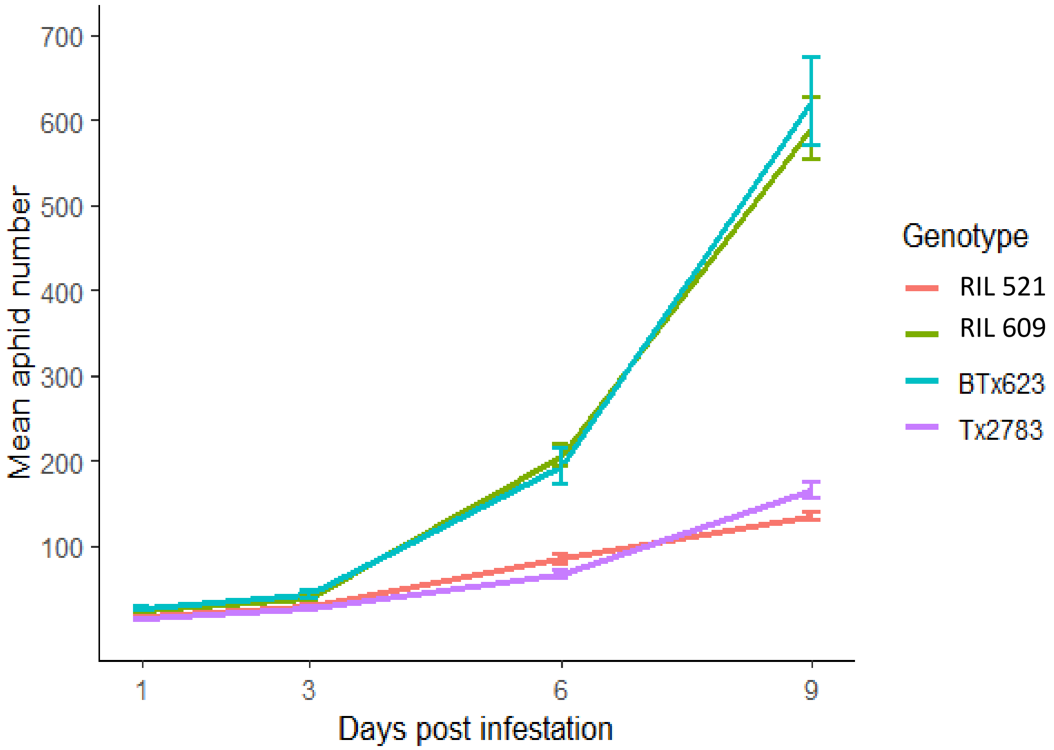

Figure S3. Line graph showing mean numbers of aphids on the four lines at 1, 3 6 and 9 dpi, respectively. Error bars represent ± standard errors (n=10). Significant differences were determined by the ANOVA and Tukey test.

Table S1. Primers for the 18 sorghum *JAZ* genes

Name	Gene ID	Forward	Reverse
SbJAZ1	Sobic.001G259300	CCACCCTGACCATCTTCTAC	GAATCTGCCATCTGCAAGAAC
SbJAZ2	Sobic.001G259400	TCGTTCCGTGGCACAAG	CGCCGTACACGATGGTTAG
SbJAZ3	Sobic.001G259600	CAGCAGCTGATGACCATCTC	CGCCATGGGCAGATCAT
SbJAZ4	Sobic.001G259700	GATGATGACCATCGTGTACGG	CTCTGTACCTGCACCTTCTTG
SbJAZ5	Sobic.001G259900	GTTCATAGAGAAGCGCAAGGA	GCACAGCATCCTCTAGCTAAC
SbJAZ6	Sobic.001G276900	GATCGATTAGCACGGACCTG	CGCCGTCACCATCAGTAG
SbJAZ7	Sobic.001G343900	CGCCAGCTGACTATCTTCTATG	GTTCTGAGCTACAGACGAACC
SbJAZ8	Sobic.001G482600	TGCTGAGCCAGTACGTCAA	ATGAGGCTCAGCGTCGTG
SbJAZ9	Sobic.001G482700	TCAAGAGGTTCCTCGAGAAGAG	TCCTTAACAGCAGGAGGCT
SbJAZ10	Sobic.001G482800	CAGCAGCTGACCATCTTCTAC	GATGGGCATGTCCACCAG
SbJAZ11	Sobic.002G036100	AGTGCAGCTACTCCTCCT	CCATGGATATGATCGCCCTT
SbJAZ12	Sobic.002G196200	AGCCATCTCCAAACCAATCC	CTTACGGGAGGAACAGCTAATG
SbJAZ13	Sobic.002G214800	GTCTCTCTGATCTGCCGATTG	AGAGCTGTTGACTTGGTATGG
SbJAZ14	Sobic.002G374100	GGTCATCAGCTTGTCTGCT	GAAGAGACGCCTTCCTCATAAT
SbJAZ15	Sobic.003G410300	TGTTCCATCTCCCACAAGATAAG	TCAACTTGGCGAGCAACT
SbJAZ16	Sobic.006G056400	GCCACTCACAAGAACCAAATC	GTTCCTCCCGATCCCATTATTC
SbJAZ17	Sobic.006G244800	AGGAAGTCGGTGAGAGGAA	TCGTAAGCTGTGCAGGATTAG
SbJAZ18	Sobic.007G132700	GTGACTAGTGTTGAGCCATACC	GGGCGATGTCTGTGAATGAA
α-Tubulin	Sobic.001G107200	GAGGTGACGATGCTTTCAACAC	CACAGGTCAACAATCTCCTTGC

Table S2. List of genes not considered as true JAZ as they are lack of TIFY, CCT or have extra domains (VEFS or GATA zinc finger)

Gene ID	Chr	Orientation	Location coordinates	Genomic (bP)	CDS (bp)	ORF (aa)	TIFY domain	CCT domain	Extra domain
Sobic.002G164300	2	reverse	50988018..51023194	35177	1844	627	N	N	VEFS
Sobic.006G015500	6	forward	2371217..2406434	35218	1821	606	N	N	VEFS
Sobic.004G042501	4	reverse	3484616..3486127	1512	729	242	Y	N	-
Sobic.001G087600	1	reverse	6791907..6803470	11564	1806	601	N	N	VEFS
Sobic.002G036150	2	reverse	3402976..3406359	3384	1029	342	Y	N	-
Sobic.001G135400	1	forward	10671949..10676384	4436	912	303	Y	Y	GATA
Sobic.001G100100	1	reverse	7655454..7660092	4639	831	276	Y	Y	GATA
Sobic.010G249200	10	reverse	58885974..58890374	4401	1059	352	Y	Y	GATA

Table S3. Summary of biochemical properties and subcellular localization of the *SbJAZ* genes

Gene name	Aliphatic index	Instability index	Subcellular localization*
SbJAZ1	62.74	46.65	chlo: 7, mito: 4, nucl: 3
SbJAZ2	78.1	60.68	chlo: 14
SbJAZ3	86.74	42.02	chlo: 12, cyto: 2
SbJAZ4	70.14	41.18	chlo: 6, nucl: 4, mito: 4
SbJAZ5	86.29	50.47	chlo: 13, mito: 1
SbJAZ6	72.99	67.25	nucl: 6, chlo: 5, cyto: 1, mito: 1, plas: 1
SbJAZ7	66.97	45.86	chlo: 10, mito: 3, vacu: 1
SbJAZ8	72.89	66.99	chlo: 10, mito: 3, nucl: 1
SbJAZ9	69.26	64.37	chlo: 10, cyto: 2, nucl: 1, mito: 1
SbJAZ10	67	83.15	chlo: 12, nucl: 2
SbJAZ11	64.79	80.86	nucl: 12, chlo: 1, cyto: 1
SbJAZ12	64.34	66.73	nucl: 12, nucl_plas: 7.5, mito: 1
SbJAZ13	69.5	42.01	chlo: 7, nucl: 2, cyto: 2, mito: 2, plas: 1
SbJAZ14	76.98	43.91	chlo: 12, mito: 1, extr: 1
SbJAZ15	59.6	45.59	nucl: 5, mito: 4, cyto: 2, chlo: 1, plas: 1, extr: 1
SbJAZ16	66.97	44.23	nucl: 7, cyto: 4, chlo: 2, mito: 1
SbJAZ17	71.87	67.44	nucl: 4.5, nucl_plas: 3.5, chlo: 3, mito: 3, cyto: 2, plas: 1.5
SbJAZ18	69.77	58.4	nucl: 6, cyto: 5, chlo: 2, mito: 1

*chlo: chloroplast; cyto: cytoplasm; mito: mitochondrion; nucl: nucleus; plas: plasma membrane; vacu: vacuole.

Table S4. The information of *JAZ* genes in different plant species

Species	Locus name/Accession number	Gene name
Physcomitrella patens	Pp1s15_168V6.1	*PpJAZ1*
	Pp1s88_112V6.1	*PpJAZ2*
	Pp1s88_114V6.1	*PpJAZ3*
	Pp1s103_27V6.1	*PpJAZ4*
	Pp1s103_29V6.2	*PpJAZ5*
	Pp1s442_10V6.1	*PpJAZ6*
	Pp1s442_14V6.1	*PpJAZ7*
Selaginella moellendorffii	439249	*SmJAZ1*
	448406	*SmJAZ2*
	415314	*SmJAZ3*
	418969	*SmJAZ4*
	427019	*SmJAZ5*
	445144	*SmJAZ6*
Picea sitchensis	ABR16076.1	*PsJAZ1*
	ABR16381.1	*PsJAZ2*
	ABR17591.1	*PsJAZ3*
	ABR18272.1	*PsJAZ4*
	ABK22803.1	*PsJAZ5*
	ABK23510.1	*PsJAZ6*
	ABK25156.1	*PsJAZ7*
	ABK26593.1	*PsJAZ8*
	ABK26702.1	*PsJAZ9*
	ABK26895.1	*PsJAZ10*
	ADE76943.1	*PsJAZ11*
	ADE77031.1	*PsJAZ12*
	ADE77685.1	*PsJAZ13*
Brassicae oleracea	Boc02g02049	*BoJAZ1*
	Boc02g02168	*BoJAZ2*
	Boc02g02274	*BoJAZ3*
	Boc02g03465	*BoJAZ4*
	Boc02g03937	*BoJAZ5*
	Boc03g01228	*BoJAZ6*
	Boc03g06386	*BoJAZ7*
	Boc04g01076	*BoJAZ8*
	Boc05g01466	*BoJAZ9*
	Boc05g02247	*BoJAZ10*
	Boc05g03357	*BoJAZ11*
	Boc06g00449	*BoJAZ12*
	Boc06g00630	*BoJAZ13*
	Boc06g00780	*BoJAZ14*
	Boc06g01385	*BoJAZ15*
	Boc06g01385	*BoJAZ15*
	Boc06g01609	*BoJAZ16*
	Boc08g00802	*BoJAZ17*
	Boc08g00884	*BoJAZ18*
	Boc08g02786	*BoJAZ19*
	Boc08g02867	*BoJAZ20*
	Boc09g04265	*BoJAZ21*

Species	Locus name/Accession number	Gene name
Arabidopsis thaliana	AT1G19180.1	*AtJAZ1*
	AT1G74950.1	*AtJAZ2*
	AT3G17860.1	*AtJAZ3*
	AT1G48500.1	*AtJAZ4*
	AT1G17380.1	*AtJAZ5*
	AT1G72450.1	*AtJAZ6*
	AT2G34600.1	*AtJAZ7*
	AT1G30135.1	*AtJAZ8*
	AT1G70700.1	*AtJAZ9*
	AT5G13220.1	*AtJAZ10*
	AT3G43440.1	*AtJAZ11*
	AT5G20900.1	*AtJAZ12*
Camelina sinensis	TEA001681.1	*CsJAZ1*
	TEA002032.1	*CsJAZ2*
	TEA001414.1	*CsJAZ3*
	TEA033836.1	*CsJAZ4*
	TEA033832.1	*CsJAZ5*
	TEA014550.1	*CsJAZ6*
	TEA004474.1	*CsJAZ7*
	TEA032228.1	*CsJAZ8*
	TEA001501.1	*CsJAZ9*
	TEA013465.1	*CsJAZ10*
	TEA001821.1	*CsJAZ11*
	TEA030190.1	*CsJAZ12*
	TEA027049.1	*CsJAZ13*
Oryza sativa	LOC_Os03g08310.1	*OsJAZ1*
	LOC_Os03g08320.1	*OsJAZ2*
	LOC_Os03g08330.1	*OsJAZ3*
	LOC_Os03g27900.1	*OsJAZ4*
	LOC_Os03g28940.1	*OsJAZ5*
	LOC_Os04g32480.1	*OsJAZ6*
	LOC_Os04g55920.2	*OsJAZ7*
	LOC_Os07g05830.1	*OsJAZ8*
	LOC_Os07g42370.1	*OsJAZ9*
	LOC_Os08g33160.1	*OsJAZ10*
	LOC_Os09g23660.1	*OsJAZ11*
	LOC_Os09g26780.1	*OsJAZ12*
	LOC_Os10g25230.1	*OsJAZ13*
	LOC_Os10g25250.1	*OsJAZ14*
	LOC_Os10g25290.1	*OsJAZ15*

Table S5. Distribution of cis elements in the promoter regions (2000bp upstream) of the *SbJAZ* genes.

Motif name	Sorghum JAZ (SbJAZ)																		Functions
	1	2	3	4	5	6	7	8	9	10	11	12	13	14	15	16	17	18	
ABRE	0	2	7	9	9	4	5	10	10	10	14	12	8	8	3	4	7	8	involved in the abscisic acid responsiveness
P-box	1	0	0	0	0	0	1	0	0	0	1	0	0	3	1	0	0	0	gibberellin-responsive element
GARE	0	0	0	0	0	1	0	0	0	0	0	0	1	0	0	2	0	0	gibberellin-responsive element
TCA-element	1	1	1	1	1	0	0	0	0	0	2	0	0	1	0	1	0	1	salicylic acid responsiveness
ERE	0	2	1	0	0	0	0	0	0	0	0	0	1	2	0	0	0	3	ethylene rich motif
TGACG-motif	3	3	2	0	9	7	1	0	2	3	2	5	3	1	0	3	1	0	involved in the MeJA-responsiveness
CGTCA-motif	3	3	2	0	9	7	1	0	2	3	2	5	3	1	0	3	1	0	involved in the MeJA-responsiveness
AuxRR-core	0	0	0	0	0	0	0	1	2	1	0	0	0	0	0	0	0	0	auxin-responsive element
TGA-element	0	0	1	0	4	0	0	2	0	0	1	1	1	0	0	2	0	1	auxin-responsive element
WRE3	1	1	0	0	1	3	1	1	4	0	2	0	0	2	2	2	2	1	-
W-box	1	2	0	0	2	3	0	0	0	0	1	1	1	0	0	1	1	1	-
WUN-motif	0	1	0	0	0	0	0	2	0	0	0	0	0	2	2	0	0	0	Wound related motif
TC-rich repeat	0	0	0	0	0	0	1	1	0	0	0	0	0	0	0	0	0	0	involved in defense and stress
CCAAT-box	2	0	1	0	0	1	0	0	1	0	0	0	1	0	0	0	2	0	stress and defense inducible element
ARE	3	1	4	3	0	1	2	1	1	1	2	2	1	1	2	4	1	3	-
AAGAA-motif	1	1	0	2	0	0	2	1	1	2	0	0	1	0	0	1	3	2	-
as-1	3	0	2	0	9	7	1	0	2	3	2	5	3	1	0	3	1	0	-
DRE1	0	0	0	0	0	0	0	0	0	0	0	0	0	0	2	0	0	1	-
LTR	0	2	3	1	0	0	2	0	0	0	1	0	1	2	0	0	1	1	involved in low-temperature responsiveness
MBS	0	2	0	1	0	3	2	4	0	2	1	0	0	0	1	1	0	0	drought inducible
MYB	3	1	4	4	4	10	8	5	13	2	8	3	3	1	6	8	1	4	-
MYC	3	1	5	3	1	0	2	5	4	2	0	6	7	4	3	3	5	3	-
Myb	0	2	0	2	1	4	4	5	1	3	2	0	1	0	3	4	1	0	drought inducible
STRE	3	4	2	1	6	1	3	5	3	4	2	4	5	4	2	2	8	2	-

Table S6. Transcription factors of the *SbJAZ* genes.

S. No.	Arabdopsis	TF ID	Family	Sorghum ID	start	stop	strand	score	p-value	matched sequence
1.	AT3G20840	AP2	AP2	SbJAZ11	1882	1900	-	8.25E-08	0.00633	CGCGCGCGCCCCGAGGAAG
2.	AT3G20840	AP2	AP2	SbJAZ18	1632	1650	-	6.87E-07	0.0263	GTCACGACTCACGAGGATG
3.	AT5G10510	AP2	AP2	SbJAZ11	1885	1899	+	1.90E-07	0.0139	CCTCGGGGCGCGCGC
4.	AT5G17430	AP2	AP2	SbJAZ17	2107	2126	+	4.40E-08	0.000393	GAGAAGGAGAGAGAGAGAGG
5.	AT5G17430	AP2	AP2	SbJAZ17	2101	2120	+	4.47E-08	0.000393	CGGAAAGAGAAGGAGAGAGA
6.	AT5G17430	AP2	AP2	SbJAZ1	1992	2011	+	4.85E-08	0.000393	GAGAGAGAGAGAGAGAGAGA
7.	AT5G17430	AP2	AP2	SbJAZ1	1994	2013	+	4.85E-08	0.000393	GAGAGAGAGAGAGAGAGAGA
8.	AT5G17430	AP2	AP2	SbJAZ1	1996	2015	+	4.85E-08	0.000393	GAGAGAGAGAGAGAGAGAGA
9.	AT5G17430	AP2	AP2	SbJAZ1	1998	2017	+	4.85E-08	0.000393	GAGAGAGAGAGAGAGAGAGA
10.	AT5G17430	AP2	AP2	SbJAZ1	2000	2019	+	4.85E-08	0.000393	GAGAGAGAGAGAGAGAGAGA
11.	AT5G17430	AP2	AP2	SbJAZ1	2002	2021	+	4.85E-08	0.000393	GAGAGAGAGAGAGAGAGAGA
12.	AT5G17430	AP2	AP2	SbJAZ1	2004	2023	+	4.85E-08	0.000393	GAGAGAGAGAGAGAGAGAGA
13.	AT5G17430	AP2	AP2	SbJAZ1	2006	2025	+	5.48E-08	0.000393	GAGAGAGAGAGAGAGAGAAA
14.	AT5G17430	AP2	AP2	SbJAZ1	2012	2031	+	5.63E-08	0.000393	GAGAGAGAGAGAAAGAGAGG
15.	AT5G17430	AP2	AP2	SbJAZ18	2068	2087	+	1.90E-07	0.00122	TAGAGAGAGAGAGAGAGAAG
16.	AT5G17430	AP2	AP2	SbJAZ1	2010	2029	+	2.08E-07	0.00123	GAGAGAGAGAGAGAAAGAGA
17.	AT5G17430	AP2	AP2	SbJAZ1	1990	2009	+	2.31E-07	0.00125	CAGAGAGAGAGAGAGAGAGA
18.	AT5G17430	AP2	AP2	SbJAZ11	1785	1804	+	2.45E-07	0.00125	AAAAAAAAAAAAAAAAAAAA
19.	AT5G17430	AP2	AP2	SbJAZ17	2103	2122	+	2.67E-07	0.00128	GAAAGAGAAGGAGAGAGAGA
20.	AT5G17430	AP2	AP2	SbJAZ1	2008	2027	+	5.50E-07	0.00249	GAGAGAGAGAGAGAGAAAGA
21.	AT5G17430	AP2	AP2	SbJAZ11	1787	1806	+	7.09E-07	0.00298	AAAAAAAAAAAAAAAAAACG
22.	AT5G17430	AP2	AP2	SbJAZ10	1825	1844	-	7.37E-07	0.00298	CGAGACGAGAGGGAGAGAGG
23.	AT5G17430	AP2	AP2	SbJAZ1	2018	2037	+	8.53E-07	0.00328	GAGAGAAAGAGAGGGAGAGG
24.	AT5G37020	ARF	ARF	SbJAZ13	1265	1273	-	8.24E-07	0.0623	GCTGTCGGC
25.	AT1G49480	B3	B3	SbJAZ11	1785	1799	+	2.02E-07	0.00246	AAAAAAAAAAAAAAA
26.	AT1G49480	B3	B3	SbJAZ11	1786	1800	+	2.02E-07	0.00246	AAAAAAAAAAAAAAA
27.	AT1G49480	B3	B3	SbJAZ11	1787	1801	+	2.02E-07	0.00246	AAAAAAAAAAAAAAA
28.	AT1G49480	B3	B3	SbJAZ11	1788	1802	+	2.02E-07	0.00246	AAAAAAAAAAAAAAA
29.	AT1G49480	B3	B3	SbJAZ11	1789	1803	+	2.02E-07	0.00246	AAAAAAAAAAAAAAA
30.	AT1G49480	B3	B3	SbJAZ11	1790	1804	+	2.02E-07	0.00246	AAAAAAAAAAAAAAA
31.	AT1G49480	B3	B3	SbJAZ11	973	987	-	2.23E-07	0.00246	CACAAAAAAAAAAAA
32.	AT1G49480	B3	B3	SbJAZ11	972	986	-	2.90E-07	0.00271	ACAAAAAAAAAAAAA
33.	AT1G49480	B3	B3	SbJAZ11	1782	1796	+	3.16E-07	0.00271	CCCAAAAAAAAAAAA
34.	AT1G49480	B3	B3	SbJAZ11	971	985	-	4.09E-07	0.00287	CAAAAAAAAAAAAAA
35.	AT1G49480	B3	B3	SbJAZ11	1784	1798	+	4.09E-07	0.00287	CAAAAAAAAAAAAAA
36.	AT1G49480	B3	B3	SbJAZ11	969	983	-	4.60E-07	0.00296	AAAAAAAAAAAAACA
37.	AT1G49480	B3	B3	SbJAZ11	1783	1797	+	5.92E-07	0.00351	CCAAAAAAAAAAAAA
38.	AT3G26790	B3	B3	SbJAZ9	1569	1583	-	3.39E-07	0.0186	ACACGCATGCATGTG
39.	AT3G26790	B3	B3	SbJAZ7	1347	1361	+	5.53E-07	0.0186	TCATGCATGCATTCA
40.	AT3G26790	B3	B3	SbJAZ10	853	867	+	7.17E-07	0.0186	AGAGGCATGCATGCA
41.	AT5G18090	B3	B3	SbJAZ6	1778	1792	-	8.31E-07	0.0645	GCAGATGAAGCAACG
42.	AT2G01930	BPC1	BBR-BPC	SbJAZ1	1992	2015	+	8.97E-16	1.36E-11	GAGAGAGAGAGAGAGAGAGAGAGA
43.	AT2G01930	BPC1	BBR-BPC	SbJAZ1	1994	2017	+	8.97E-16	1.36E-11	GAGAGAGAGAGAGAGAGAGAGAGA
44.	AT2G01930	BPC1	BBR-BPC	SbJAZ1	1996	2019	+	8.97E-16	1.36E-11	GAGAGAGAGAGAGAGAGAGAGAGA
45.	AT2G01930	BPC1	BBR-BPC	SbJAZ1	1998	2021	+	8.97E-16	1.36E-11	GAGAGAGAGAGAGAGAGAGAGAGA
46.	AT2G01930	BPC1	BBR-BPC	SbJAZ1	2000	2023	+	8.97E-16	1.36E-11	GAGAGAGAGAGAGAGAGAGAGAGA
47.	AT2G01930	BPC1	BBR-BPC	SbJAZ1	2004	2027	+	9.42E-15	1.19E-10	GAGAGAGAGAGAGAGAGAGAAAGA
48.	AT2G01930	BPC1	BBR-BPC	SbJAZ1	2006	2029	+	1.12E-14	1.22E-10	GAGAGAGAGAGAGAGAGAAAGAGA
49.	AT2G01930	BPC1	BBR-BPC	SbJAZ1	2002	2025	+	2.01E-14	1.91E-10	GAGAGAGAGAGAGAGAGAGAGAAA
50.	AT2G01930	BPC1	BBR-BPC	SbJAZ1	1990	2013	+	3.22E-14	2.72E-10	CAGAGAGAGAGAGAGAGAGAGAGA
51.	AT2G01930	BPC1	BBR-BPC	SbJAZ1	2010	2033	+	1.28E-13	8.80E-10	GAGAGAGAGAGAGAAAGAGAGGGA
52.	AT2G01930	BPC1	BBR-BPC	SbJAZ1	2012	2035	+	1.28E-13	8.80E-10	GAGAGAGAGAGAAAGAGAGGGAGA
53.	AT2G01930	BPC1	BBR-BPC	SbJAZ1	2008	2031	+	1.50E-13	9.52E-10	GAGAGAGAGAGAGAGAAAGAGAGG
54.	AT2G01930	BPC1	BBR-BPC	SbJAZ1	1988	2011	+	1.45E-12	8.48E-09	CACAGAGAGAGAGAGAGAGAGAGA
55.	AT2G01930	BPC1	BBR-BPC	SbJAZ1	2014	2037	+	1.05E-11	5.67E-08	GAGAGAGAGAAAGAGAGGGAGAGG
56.	AT2G01930	BPC1	BBR-BPC	SbJAZ1	2016	2039	+	2.04E-10	1.03E-06	GAGAGAGAAAGAGAGGGAGAGGCC
57.	AT2G01930	BPC1	BBR-BPC	SbJAZ1	2018	2041	+	2.96E-10	1.40E-06	GAGAGAAAGAGAGGGAGAGGCCGA
58.	AT2G01930	BPC1	BBR-BPC	SbJAZ1	1986	2009	+	7.24E-10	3.05E-06	TGCACAGAGAGAGAGAGAGAGAGA
59.	AT2G01930	BPC1	BBR-BPC	SbJAZ17	2105	2128	+	7.24E-10	3.05E-06	AAGAGAAGGAGAGAGAGAGAGGAG
60.	AT2G01930	BPC1	BBR-BPC	SbJAZ17	2103	2126	+	1.80E-09	7.18E-06	GAAAGAGAAGGAGAGAGAGAGAGG
61.	AT2G01930	BPC1	BBR-BPC	SbJAZ17	2099	2122	+	3.61E-09	1.37E-05	GGCGGAAAGAGAAGGAGAGAGAGA
62.	AT2G01930	BPC1	BBR-BPC	SbJAZ17	2101	2124	+	5.52E-09	1.98E-05	CGGAAAGAGAAGGAGAGAGAGAGA
63.	AT2G01930	BPC1	BBR-BPC	SbJAZ17	2107	2130	+	5.74E-09	1.98E-05	GAGAAGGAGAGAGAGAGAGGAGTT
64.	AT2G01930	BPC1	BBR-BPC	SbJAZ18	2068	2091	+	1.26E-08	4.14E-05	TAGAGAGAGAGAGAGAGAAGAGAG
65.	AT2G01930	BPC1	BBR-BPC	SbJAZ18	2064	2087	+	1.49E-08	4.71E-05	GGTTTAGAGAGAGAGAGAGAGAAG
66.	AT2G01930	BPC1	BBR-BPC	SbJAZ12	2189	2212	-	4.20E-08	1.25E-04	GTGGAATGGAGAGAGAGAGACGGA
67.	AT2G01930	BPC1	BBR-BPC	SbJAZ18	2060	2083	+	4.29E-08	1.25E-04	AAGTGGTTTAGAGAGAGAGAGAGA
68.	AT2G01930	BPC1	BBR-BPC	SbJAZ18	2066	2089	+	4.83E-08	0.000136	TTTAGAGAGAGAGAGAGAGAAGAG

69.	AT2G01930	BPC1	BBR-BPC	SbJAZ18	2072	2095	+	6.94E-08	0.000188	GAGAGAGAGAGAGAAGAGAGAGGA
70.	AT2G01930	BPC1	BBR-BPC	SbJAZ1	1984	2007	+	7.40E-08	0.000194	ACTGCACAGAGAGAGAGAGAGAGA
71.	AT2G01930	BPC1	BBR-BPC	SbJAZ18	2070	2093	+	7.72E-08	0.000196	GAGAGAGAGAGAGAGAAGAGAGAG
72.	AT2G01930	BPC1	BBR-BPC	SbJAZ18	699	722	+	8.97E-08	0.00022	AGGAGAGAAAGAAGTAGAGAGAAG
73.	AT2G01930	BPC1	BBR-BPC	SbJAZ17	2097	2120	+	9.98E-08	0.000237	GCGGCGGAAAGAGAAGGAGAGAGA
74.	AT2G01930	BPC1	BBR-BPC	SbJAZ17	2113	2136	+	1.39E-07	0.000319	GAGAGAGAGAGAGGAGTTGCAAGT
75.	AT2G01930	BPC1	BBR-BPC	SbJAZ1	479	502	+	1.70E-07	0.000361	AAGAGAGAGAGAGAGAGGTGCAAT
76.	AT2G01930	BPC1	BBR-BPC	SbJAZ12	2185	2208	-	1.73E-07	0.000361	AATGGAGAGAGAGAGACGGACAGC
77.	AT2G01930	BPC1	BBR-BPC	SbJAZ18	2117	2140	+	1.74E-07	0.000361	GTGGGAGAGAGAAAGGTTAAGAGG
78.	AT2G01930	BPC1	BBR-BPC	SbJAZ7	2092	2115	+	1.78E-07	0.000361	GAAGGGGAAAGAGGAGGAGAGCGA
79.	AT2G01930	BPC1	BBR-BPC	SbJAZ18	2074	2097	+	1.80E-07	0.000361	GAGAGAGAGAGAAGAGAGAGGAGG
80.	AT2G01930	BPC1	BBR-BPC	SbJAZ10	1122	1145	+	2.17E-07	0.000406	AGCAGATATAGAGAGAGAGAGACT
81.	AT2G01930	BPC1	BBR-BPC	SbJAZ18	697	720	+	2.19E-07	0.000406	GGAGGAGAGAAAGAAGTAGAGAGA
82.	AT2G01930	BPC1	BBR-BPC	SbJAZ12	2181	2204	-	2.22E-07	0.000406	GAGAGAGAGAGACGGACAGCTCGG
83.	AT2G01930	BPC1	BBR-BPC	SbJAZ10	1124	1147	+	2.25E-07	0.000406	CAGATATAGAGAGAGAGAGACTAG
84.	AT2G01930	BPC1	BBR-BPC	SbJAZ8	1724	1747	-	2.66E-07	0.00047	GAGAGAGAGAGAGGTGTGGTGCGG
85.	AT2G01930	BPC1	BBR-BPC	SbJAZ8	1730	1753	-	2.79E-07	0.000474	GCGGGGGAGAGAGAGAGAGGTGTG
86.	AT2G01930	BPC1	BBR-BPC	SbJAZ1	477	500	+	2.81E-07	0.000474	ATAAGAGAGAGAGAGAGAGGTGCA
87.	AT2G01930	BPC1	BBR-BPC	SbJAZ10	1126	1149	+	3.97E-07	0.000656	GATATAGAGAGAGAGAGACTAGGG
88.	AT2G01930	BPC1	BBR-BPC	SbJAZ8	1726	1749	-	4.35E-07	0.000703	GGGAGAGAGAGAGAGGTGTGGTGC
89.	AT2G01930	BPC1	BBR-BPC	SbJAZ17	372	395	+	5.23E-07	0.000827	GAGGAGGGGGGAGGGGGAGGGGGT
90.	AT2G01930	BPC1	BBR-BPC	SbJAZ12	2187	2210	-	7.26E-07	0.00111	GGAATGGAGAGAGAGAGACGGACA
91.	AT2G01930	BPC1	BBR-BPC	SbJAZ18	2062	2085	+	7.31E-07	0.00111	GTGGTTTAGAGAGAGAGAGAGAGA
92.	AT2G01930	BPC1	BBR-BPC	SbJAZ1	475	498	+	7.50E-07	0.00112	ACATAAGAGAGAGAGAGAGAGGTG
93.	AT2G01930	BPC1	BBR-BPC	SbJAZ10	1118	1141	+	8.04E-07	0.00116	GGCAAGCAGATATAGAGAGAGAGA
94.	AT2G01930	BPC1	BBR-BPC	SbJAZ10	1579	1602	+	8.15E-07	0.00116	TAGAAAGAGAACGAAAGAAACGGA
95.	AT2G01930	BPC1	BBR-BPC	SbJAZ1	2020	2043	+	8.25E-07	0.00116	GAGAAAGAGAGGGAGAGGCCGACG
96.	AT2G01930	BPC1	BBR-BPC	SbJAZ18	2087	2110	+	9.36E-07	0.00129	GAGAGAGGAGGAAAGGGAAAGCAG
97.	AT2G01930	BPC1	BBR-BPC	SbJAZ18	701	724	+	9.73E-07	0.0013	GAGAGAAAGAAGTAGAGAGAAGAG
98.	AT2G01930	BPC1	BBR-BPC	SbJAZ1	481	504	+	9.79E-07	0.0013	GAGAGAGAGAGAGAGGTGCAATCG
99.	AT4G38910	BPC5	BBR-BPC	SbJAZ1	1991	2020	+	1.55E-19	5.89E-15	AGAGAGAGAGAGAGAGAGAGAGAGAGAGAG
100.	AT4G38910	BPC5	BBR-BPC	SbJAZ1	1993	2022	+	1.55E-19	5.89E-15	AGAGAGAGAGAGAGAGAGAGAGAGAGAGAG
101.	AT4G38910	BPC5	BBR-BPC	SbJAZ1	1995	2024	+	1.55E-18	3.93E-14	AGAGAGAGAGAGAGAGAGAGAGAGAGAGAA
102.	AT4G38910	BPC5	BBR-BPC	SbJAZ1	1999	2028	+	8.46E-18	1.28E-13	AGAGAGAGAGAGAGAGAGAGAGAGAAAGAG
103.	AT4G38910	BPC5	BBR-BPC	SbJAZ1	2001	2030	+	8.46E-18	1.28E-13	AGAGAGAGAGAGAGAGAGAGAGAAAGAGAG
104.	AT4G38910	BPC5	BBR-BPC	SbJAZ1	2007	2036	+	5.49E-17	6.94E-13	AGAGAGAGAGAGAGAGAAAGAGAGGGAGAG
105.	AT4G38910	BPC5	BBR-BPC	SbJAZ1	1997	2026	+	1.01E-16	9.64E-13	AGAGAGAGAGAGAGAGAGAGAGAGAGAAAG
106.	AT4G38910	BPC5	BBR-BPC	SbJAZ1	1989	2018	+	1.02E-16	9.64E-13	ACAGAGAGAGAGAGAGAGAGAGAGAGAGAG
107.	AT4G38910	BPC5	BBR-BPC	SbJAZ1	2005	2034	+	2.15E-15	1.81E-11	AGAGAGAGAGAGAGAGAGAAAGAGAGGGAG
108.	AT4G38910	BPC5	BBR-BPC	SbJAZ1	2003	2032	+	1.69E-14	1.28E-10	AGAGAGAGAGAGAGAGAGAGAAAGAGAGGG
109.	AT4G38910	BPC5	BBR-BPC	SbJAZ1	1987	2016	+	7.62E-14	5.26E-10	GCACAGAGAGAGAGAGAGAGAGAGAGAGAG
110.	AT4G38910	BPC5	BBR-BPC	SbJAZ1	2011	2040	+	3.91E-13	2.48E-09	AGAGAGAGAGAGAAAGAGAGGGAGAGGCCG
111.	AT4G38910	BPC5	BBR-BPC	SbJAZ1	2009	2038	+	9.37E-13	5.47E-09	AGAGAGAGAGAGAGAAAGAGAGGGAGAGGC
112.	AT4G38910	BPC5	BBR-BPC	SbJAZ1	2013	2042	+	3.40E-11	1.84E-07	AGAGAGAGAGAAAGAGAGGGAGAGGCCGAC
113.	AT4G38910	BPC5	BBR-BPC	SbJAZ1	1983	2012	+	1.26E-09	6.36E-06	GACTGCACAGAGAGAGAGAGAGAGAGAGAG
114.	AT4G38910	BPC5	BBR-BPC	SbJAZ18	2057	2086	+	1.41E-09	6.68E-06	AGAAAGTGGTTTAGAGAGAGAGAGAGAGAA
115.	AT4G38910	BPC5	BBR-BPC	SbJAZ18	2055	2084	+	2.45E-09	1.09E-05	GGAGAAAGTGGTTTAGAGAGAGAGAGAGAG
116.	AT4G38910	BPC5	BBR-BPC	SbJAZ1	1985	2014	+	2.72E-09	1.15E-05	CTGCACAGAGAGAGAGAGAGAGAGAGAGAG
117.	AT4G38910	BPC5	BBR-BPC	SbJAZ1	2015	2044	+	6.69E-09	2.67E-05	AGAGAGAGAAAGAGAGGGAGAGGCCGACGC
118.	AT4G38910	BPC5	BBR-BPC	SbJAZ17	2096	2125	+	1.42E-08	5.40E-05	AGCGGCGGAAAGAGAAGGAGAGAGAGAGAG
119.	AT4G38910	BPC5	BBR-BPC	SbJAZ17	2104	2133	+	1.96E-08	7.09E-05	AAAGAGAAGGAGAGAGAGAGAGGAGTTGCA
120.	AT4G38910	BPC5	BBR-BPC	SbJAZ1	2017	2046	+	2.75E-08	9.50E-05	AGAGAGAAAGAGAGGGAGAGGCCGACGCAC
121.	AT4G38910	BPC5	BBR-BPC	SbJAZ1	1981	2010	+	2.88E-08	9.52E-05	GAGACTGCACAGAGAGAGAGAGAGAGAGAG
122.	AT4G38910	BPC5	BBR-BPC	SbJAZ1	2019	2048	+	8.67E-08	0.000274	AGAGAAAGAGAGGGAGAGGCCGACGCACCA
123.	AT4G38910	BPC5	BBR-BPC	SbJAZ17	2102	2131	+	1.13E-07	0.000344	GGAAAGAGAAGGAGAGAGAGAGAGGAGTTG
124.	AT4G38910	BPC5	BBR-BPC	SbJAZ10	1574	1603	+	1.53E-07	0.000446	AGAAATAGAAAGAGAACGAAAGAAACGGAC
125.	AT4G38910	BPC5	BBR-BPC	SbJAZ17	2094	2123	+	1.71E-07	0.000482	CAAGCGGCGGAAAGAGAAGGAGAGAGAGAG
126.	AT4G38910	BPC5	BBR-BPC	SbJAZ1	466	495	+	1.98E-07	0.000538	AGGAATAGGACATAAGAGAGAGAGAGAGAG
127.	AT4G38910	BPC5	BBR-BPC	SbJAZ18	2069	2098	+	2.22E-07	0.000582	AGAGAGAGAGAGAGAGAAGAGAGAGGAGGA
128.	AT4G38910	BPC5	BBR-BPC	SbJAZ18	2059	2088	+	2.36E-07	0.000596	AAAGTGGTTTAGAGAGAGAGAGAGAGAAGA
129.	AT4G38910	BPC5	BBR-BPC	SbJAZ10	1123	1152	+	2.89E-07	0.000707	GCAGATATAGAGAGAGAGAGACTAGGGCCT
130.	AT4G38910	BPC5	BBR-BPC	SbJAZ12	2184	2213	-	3.61E-07	0.000857	GGTGGAATGGAGAGAGAGAGACGGACAGCT
131.	AT4G38910	BPC5	BBR-BPC	SbJAZ18	2053	2082	+	4.20E-07	0.000967	GGGGAGAAAGTGGTTTAGAGAGAGAGAGAG
132.	AT4G38910	BPC5	BBR-BPC	SbJAZ18	2047	2076	+	4.70E-07	0.00105	AGCGAGGGGGAGAAAGTGGTTTAGAGAGAG
133.	AT4G38910	BPC5	BBR-BPC	SbJAZ10	1568	1597	+	5.18E-07	0.00112	AGAAACAGAAATAGAAAGAGAACGAAAGAA
134.	AT4G38910	BPC5	BBR-BPC	SbJAZ18	2051	2080	+	7.67E-07	0.00162	AGGGGGAGAAAGTGGTTTAGAGAGAGAGAG
135.	AT4G38910	BPC5	BBR-BPC	SbJAZ18	2073	2102	+	9.37E-07	0.00192	AGAGAGAGAGAGAAGAGAGAGGAGGAAAGG
136.	AT5G42520	BPC6	BBR-BPC	SbJAZ1	1992	2012	-	4.84E-14	6.22E-10	CTCTCTCTCTCTCTCTCTCTC
137.	AT5G42520	BPC6	BBR-BPC	SbJAZ1	1994	2014	-	4.84E-14	6.22E-10	CTCTCTCTCTCTCTCTCTCTC
138.	AT5G42520	BPC6	BBR-BPC	SbJAZ1	1996	2016	-	4.84E-14	6.22E-10	CTCTCTCTCTCTCTCTCTCTC
139.	AT5G42520	BPC6	BBR-BPC	SbJAZ1	1998	2018	-	4.84E-14	6.22E-10	CTCTCTCTCTCTCTCTCTCTC
140.	AT5G42520	BPC6	BBR-BPC	SbJAZ1	2000	2020	-	4.84E-14	6.22E-10	CTCTCTCTCTCTCTCTCTCTC

141.	AT5G42520	BPC6	BBR-BPC	SbJAZ1	2002	2022	-	4.84E-14	6.22E-10	CTCTCTCTCTCTCTCTCTCTC
142.	AT5G42520	BPC6	BBR-BPC	SbJAZ1	2004	2024	-	3.13E-13	3.45E-09	TTCTCTCTCTCTCTCTCTCTC
143.	AT5G42520	BPC6	BBR-BPC	SbJAZ1	2008	2028	-	4.09E-13	3.94E-09	CTCTTTCTCTCTCTCTCTCTC
144.	AT5G42520	BPC6	BBR-BPC	SbJAZ1	2010	2030	-	6.74E-13	5.77E-09	CTCTCTTTCTCTCTCTCTCTC
145.	AT5G42520	BPC6	BBR-BPC	SbJAZ1	2006	2026	-	4.15E-12	3.20E-08	CTTTCTCTCTCTCTCTCTCTC
146.	AT5G42520	BPC6	BBR-BPC	SbJAZ1	1990	2010	-	6.97E-12	4.88E-08	CTCTCTCTCTCTCTCTCTCTG
147.	AT5G42520	BPC6	BBR-BPC	SbJAZ1	2012	2032	-	9.95E-12	6.39E-08	CCCTCTCTTTCTCTCTCTCTC
148.	AT5G42520	BPC6	BBR-BPC	SbJAZ1	2014	2034	-	1.20E-11	7.12E-08	CTCCCTCTCTTTCTCTCTCTC
149.	AT5G42520	BPC6	BBR-BPC	SbJAZ18	2068	2088	-	6.37E-10	3.50E-06	TCTTCTCTCTCTCTCTCTCTA
150.	AT5G42520	BPC6	BBR-BPC	SbJAZ17	2105	2125	-	2.39E-09	1.23E-05	CTCTCTCTCTCTCCTTCTCTT
151.	AT5G42520	BPC6	BBR-BPC	SbJAZ17	2107	2127	-	3.50E-09	1.68E-05	TCCTCTCTCTCTCTCCTTCTC
152.	AT5G42520	BPC6	BBR-BPC	SbJAZ18	2066	2086	-	4.05E-09	1.84E-05	TTCTCTCTCTCTCTCTCTAAA
153.	AT5G42520	BPC6	BBR-BPC	SbJAZ1	2018	2038	-	4.78E-09	2.05E-05	GCCTCTCCCTCTCTTTCTCTC
154.	AT5G42520	BPC6	BBR-BPC	SbJAZ18	2070	2090	-	7.61E-09	3.09E-05	TCTCTTCTCTCTCTCTCTCTC
155.	AT5G42520	BPC6	BBR-BPC	SbJAZ1	2016	2036	-	9.49E-09	3.65E-05	CTCTCCCTCTCTTTCTCTCTC
156.	AT5G42520	BPC6	BBR-BPC	SbJAZ8	1733	1753	+	1.44E-08	5.30E-05	ACCTCTCTCTCTCTCCCCCGC
157.	AT5G42520	BPC6	BBR-BPC	SbJAZ1	1988	2008	-	1.81E-08	6.09E-05	CTCTCTCTCTCTCTCTCTGTG
158.	AT5G42520	BPC6	BBR-BPC	SbJAZ1	477	497	-	1.87E-08	6.09E-05	ACCTCTCTCTCTCTCTCTTAT
159.	AT5G42520	BPC6	BBR-BPC	SbJAZ1	479	499	-	1.90E-08	6.09E-05	GCACCTCTCTCTCTCTCTCTT
160.	AT5G42520	BPC6	BBR-BPC	SbJAZ18	2064	2084	-	2.11E-08	6.49E-05	CTCTCTCTCTCTCTCTAAACC
161.	AT5G42520	BPC6	BBR-BPC	SbJAZ1	481	501	-	2.37E-08	7.01E-05	TTGCACCTCTCTCTCTCTCTC
162.	AT5G42520	BPC6	BBR-BPC	SbJAZ10	1126	1146	-	2.51E-08	7.16E-05	TAGTCTCTCTCTCTCTATATC
163.	AT5G42520	BPC6	BBR-BPC	SbJAZ10	1124	1144	-	5.42E-08	0.000149	GTCTCTCTCTCTCTATATCTG
164.	AT5G42520	BPC6	BBR-BPC	SbJAZ12	2186	2206	+	6.47E-08	0.000167	CTGTCCGTCTCTCTCTCTCCA
165.	AT5G42520	BPC6	BBR-BPC	SbJAZ8	1731	1751	+	6.51E-08	0.000167	ACACCTCTCTCTCTCTCCCCC
166.	AT5G42520	BPC6	BBR-BPC	SbJAZ17	2109	2129	-	1.01E-07	0.000251	ACTCCTCTCTCTCTCTCCTTC
167.	AT5G42520	BPC6	BBR-BPC	SbJAZ10	1128	1148	-	1.08E-07	0.000259	CCTAGTCTCTCTCTCTCTATA
168.	AT5G42520	BPC6	BBR-BPC	SbJAZ18	2062	2082	-	1.47E-07	0.000343	CTCTCTCTCTCTCTAAACCAC
169.	AT5G42520	BPC6	BBR-BPC	SbJAZ1	475	495	-	2.04E-07	0.000452	CTCTCTCTCTCTCTCTTATGT
170.	AT5G42520	BPC6	BBR-BPC	SbJAZ8	1729	1749	+	2.06E-07	0.000452	CCACACCTCTCTCTCTCTCCC
171.	AT5G42520	BPC6	BBR-BPC	SbJAZ12	2190	2210	+	2.41E-07	0.000516	CCGTCTCTCTCTCTCCATTCC
172.	AT5G42520	BPC6	BBR-BPC	SbJAZ1	1986	2006	-	2.52E-07	0.000525	CTCTCTCTCTCTCTCTGTGCA
173.	AT5G42520	BPC6	BBR-BPC	SbJAZ18	2072	2092	-	4.46E-07	0.000903	TCTCTCTTCTCTCTCTCTCTC
174.	AT5G42520	BPC6	BBR-BPC	SbJAZ12	2188	2208	+	4.68E-07	0.000924	GTCCGTCTCTCTCTCTCCATT
175.	AT5G42520	BPC6	BBR-BPC	SbJAZ10	1130	1150	-	4.91E-07	0.000946	GCCCTAGTCTCTCTCTCTCTA
176.	AT5G42520	BPC6	BBR-BPC	SbJAZ18	701	721	-	5.05E-07	0.000948	TTCTCTCTACTTCTTTCTCTC
177.	AT1G19350	BES1	BES1	SbJAZ18	1712	1722	+	2.32E-08	1.60E-03	CGCACGTGGGC
178.	AT1G19350	BES1	BES1	SbJAZ11	1907	1917	-	6.87E-07	0.0174	CGCACGTGGAG
179.	AT1G19350	BES1	BES1	SbJAZ11	1420	1430	-	7.56E-07	0.0174	CACACGTGTCG
180.	AT1G75080	BES1	BES1	SbJAZ11	1907	1926	+	3.74E-07	0.0248	CTCCACGTGCGCGCCTCGCG
181.	AT1G78700	BES1	BES1	SbJAZ18	1712	1722	+	4.33E-07	0.029	CGCACGTGGGC
182.	AT4G18890	BES1	BES1	SbJAZ18	1712	1722	+	6.94E-07	0.0504	CGCACGTGGGC
183.	AT4G36780	BES1	BES1	SbJAZ18	1712	1722	-	4.10E-07	0.0296	GCCCACGTGCG
184.	AT1G10120	BHLH74	bHLH	SbJAZ18	1689	1709	-	3.10E-07	0.00839	GGGATCCGGCACGTGCCCGCA
185.	AT1G10120	BHLH74	bHLH	SbJAZ18	1865	1885	-	3.30E-07	0.00839	CGAGGCCCCCACGTGGTACGT
186.	AT1G10120	BHLH74	bHLH	SbJAZ11	1901	1921	+	5.24E-07	0.00839	CGCGTTCTCCACGTGCGCGCC
187.	AT1G10120	BHLH74	bHLH	SbJAZ8	1875	1895	-	5.94E-07	0.00839	CACGAAGAACACGTGATTCCT
188.	AT1G10120	BHLH74	bHLH	SbJAZ18	1686	1706	+	6.36E-07	0.00839	GGCTGCGGGCACGTGCCGGAT
189.	AT1G59640	BPE	bHLH	SbJAZ18	1687	1707	-	9.00E-08	0.00348	GATCCGGCACGTGCCCGCAGC
190.	AT1G59640	BPE	bHLH	SbJAZ12	1242	1262	+	1.16E-07	0.00348	CAGGAAGCACGTGGGACCCGT
191.	AT1G59640	BPE	bHLH	SbJAZ18	1688	1708	+	1.49E-07	0.00348	CTGCGGGCACGTGCCGGATCC
192.	AT1G59640	BPE	bHLH	SbJAZ12	1503	1523	+	5.86E-07	0.009	GCCGTACCACGTGGGGGAGTC
193.	AT1G59640	BPE	bHLH	SbJAZ18	1864	1884	+	6.45E-07	0.009	TACGTACCACGTGGGGGCCTC
194.	AT1G69010	BPE	bHLH	SbJAZ11	1910	1924	+	3.10E-07	0.0222	CACGTGCGCGCCTCG
195.	AT2G18300	BHLH64	bHLH	SbJAZ18	1691	1702	+	3.89E-09	2.60E-04	CGGGCACGTGCC
196.	AT2G18300	BHLH64	bHLH	SbJAZ18	1693	1704	-	1.55E-08	5.20E-04	CCGGCACGTGCC
197.	AT2G18300	BHLH64	bHLH	SbJAZ14	1884	1895	-	3.10E-08	6.92E-04	CGCGCACGTGCT
198.	AT2G18300	BHLH64	bHLH	SbJAZ17	1594	1605	-	5.53E-07	0.00817	CATGCACGTGCC
199.	AT2G18300	BHLH64	bHLH	SbJAZ11	1908	1919	-	6.10E-07	0.00817	CGCGCACGTGGA
200.	AT2G18300	BHLH64	bHLH	SbJAZ17	1592	1603	+	8.44E-07	0.00873	GTGGCACGTGCA
201.	AT2G18300	BHLH64	bHLH	SbJAZ7	1685	1696	+	9.12E-07	0.00873	GCTGCACGTGCA
202.	AT2G20180	BHLH15	bHLH	SbJAZ12	1339	1352	-	8.53E-07	0.0563	AGGACGACACGTGT
203.	AT2G22750	BHLH18	bHLH	SbJAZ11	1901	1917	+	4.05E-08	3.03E-03	CGCGTTCTCCACGTGCG
204.	AT2G22750	BHLH18	bHLH	SbJAZ13	1951	1967	-	2.48E-07	0.00929	CGCATGAAACACGTGAC
205.	AT2G22750	BHLH18	bHLH	SbJAZ12	1247	1263	-	5.24E-07	0.0112	AACGGGTCCCACGTGCT
206.	AT2G22750	BHLH18	bHLH	SbJAZ9	2100	2116	+	6.11E-07	0.0112	CTCGCCTTTCACGTGCT
207.	AT2G22750	BHLH18	bHLH	SbJAZ14	1877	1893	+	8.91E-07	0.0112	CACACGAAGCACGTGCG
208.	AT2G22750	BHLH18	bHLH	SbJAZ13	268	284	-	9.00E-07	0.0112	CACATGTTTCACGAGGG
209.	AT2G24260	BHLH66	bHLH	SbJAZ18	1693	1702	+	2.76E-07	0.00786	GGCACGTGCC
210.	AT2G24260	BHLH66	bHLH	SbJAZ18	1693	1702	-	2.76E-07	0.00786	GGCACGTGCC
211.	AT2G24260	BHLH66	bHLH	SbJAZ18	1712	1721	+	4.14E-07	0.00786	CGCACGTGGG
212.	AT2G24260	BHLH66	bHLH	SbJAZ18	1712	1721	-	4.14E-07	0.00786	CCCACGTGCG
213.	AT3G23210	bHLH34	bHLH	SbJAZ18	1687	1709	+	2.91E-08	1.06E-03	GCTGCGGGCACGTGCCGGATCCC
214.	AT3G23210	bHLH34	bHLH	SbJAZ18	1686	1708	-	3.18E-08	1.06E-03	GGATCCGGCACGTGCCCGCAGCC
215.	AT3G23210	bHLH34	bHLH	SbJAZ11	1902	1924	+	2.90E-07	0.00583	GCGTTCTCCACGTGCGCGCCTCG

216.	AT3G23210	bHLH34	bHLH	SbJAZ14	2080	2102	-	3.50E-07	0.00583	GAGCACGCCGCGCGCCCCCACGC
217.	AT3G23210	bHLH34	bHLH	SbJAZ18	1863	1885	+	5.17E-07	0.00603	GTACGTACCACGTGGGGGCCTCG
218.	AT3G23210	bHLH34	bHLH	SbJAZ10	207	229	-	5.84E-07	0.00603	GTGCAAGCCGCGTGCACGCGGGC
219.	AT3G23210	bHLH34	bHLH	SbJAZ11	1901	1923	-	6.33E-07	0.00603	GAGGCGCGCACGTGGAGAACGCG
220.	AT3G23210	bHLH34	bHLH	SbJAZ12	1331	1353	-	7.62E-07	0.00635	AAGGACGACACGTGTCCGGGAAG
221.	AT3G23210	bHLH34	bHLH	SbJAZ12	206	228	-	8.91E-07	0.0066	GTGTCCCCCACGCGCGGGAGTAT
222.	AT3G23690	bHLH34	bHLH	SbJAZ18	1688	1702	+	9.14E-07	0.0651	CTGCGGGCACGTGCC
223.	AT4G14410	bHLH104	bHLH	SbJAZ18	1693	1702	+	6.91E-08	0.00241	GGCACGTGCC
224.	AT4G14410	bHLH104	bHLH	SbJAZ18	1693	1702	-	6.91E-08	0.00241	GGCACGTGCC
225.	AT4G21330	bHLH104	bHLH	SbJAZ13	1952	1965	-	1.39E-07	0.0108	CATGAAACACGTGA
226.	AT4G30980	BHLH69	bHLH	SbJAZ18	1871	1885	-	1.68E-07	0.00543	CGAGGCCCCCACGTG
227.	AT4G30980	BHLH69	bHLH	SbJAZ11	1901	1915	+	2.01E-07	0.00543	CGCGTTCTCCACGTG
228.	AT4G30980	BHLH69	bHLH	SbJAZ11	1910	1924	-	2.59E-07	0.00543	CGAGGCGCGCACGTG
229.	AT4G36930	BHLH24	bHLH	SbJAZ12	1248	1256	-	4.12E-07	0.0149	CCCACGTGC
230.	AT4G36930	BHLH24	bHLH	SbJAZ18	1713	1721	-	4.12E-07	0.0149	CCCACGTGC
231.	AT4G36930	BHLH24	bHLH	SbJAZ12	1509	1517	-	8.24E-07	0.0149	CCCACGTGG
232.	AT4G36930	BHLH24	bHLH	SbJAZ18	1870	1878	-	8.24E-07	0.0149	CCCACGTGG
233.	AT5G08130	BHLH24	bHLH	SbJAZ13	1945	1958	-	9.10E-07	0.0644	CACGTGACTGCCGG
234.	AT5G61270	BHLH72	bHLH	SbJAZ18	1870	1884	-	1.18E-07	0.00826	GAGGCCCCCACGTGG
235.	AT5G61270	BHLH72	bHLH	SbJAZ12	1248	1262	-	3.46E-07	0.0121	ACGGGTCCCACGTGC
236.	AT1G06850	bZIP	bZIP	SbJAZ12	2174	2187	+	7.46E-07	0.0547	GTGCCAGCCGAGCT
237.	AT1G32150	bZIP68	bZIP	SbJAZ13	1190	1204	+	4.02E-07	0.0295	TGCCACGTCGTCGGA
238.	AT1G45249	BZIP36	bZIP	SbJAZ13	1185	1202	+	9.19E-07	0.0695	TGAGATGCCACGTCGTCG
239.	AT1G49720	BZIP35	bZIP	SbJAZ12	1330	1348	-	3.78E-08	2.26E-03	CGACACGTGTCCGGGAAGG
240.	AT1G49720	BZIP35	bZIP	SbJAZ18	1543	1561	-	1.58E-07	0.00364	GCCCGCGTGGCGTGCGCGC
241.	AT1G49720	BZIP35	bZIP	SbJAZ13	1756	1774	+	1.82E-07	0.00364	GATCACGTGTCGCTAGCGC
242.	AT2G18160	bZIP2	bZIP	SbJAZ13	1190	1204	-	8.47E-07	0.0622	TCCGACGACGTGGCA
243.	AT2G35530	BZIP39	bZIP	SbJAZ13	1190	1204	-	7.12E-07	0.0487	TCCGACGACGTGGCA
244.	AT2G36270	BZIP39	bZIP	SbJAZ13	1188	1205	-	8.03E-07	0.058	ATCCGACGACGTGGCATC
245.	AT2G46270	BZIP39	bZIP	SbJAZ13	1190	1204	+	4.70E-07	0.0332	TGCCACGTCGTCGGA
246.	AT3G10800	BZIP39	bZIP	SbJAZ13	1190	1204	-	9.75E-08	0.00734	TCCGACGACGTGGCA
247.	AT3G62420	BZIP53	bZIP	SbJAZ6	468	482	-	5.46E-07	0.0385	ACAGCTGACGTGACA
248.	AT4G34590	BZIP11	bZIP	SbJAZ6	468	482	+	6.46E-07	0.0454	TGTCACGTCAGCTGT
249.	AT4G36730	BZIP41	bZIP	SbJAZ17	1171	1180	+	2.75E-07	0.00902	CGACGTGGCA
250.	AT4G36730	BZIP41	bZIP	SbJAZ13	1190	1199	-	2.75E-07	0.00902	CGACGTGGCA
251.	AT5G11260	BZIP56	bZIP	SbJAZ6	1688	1702	-	4.37E-07	0.0314	GGTGGTGACGTGTTC
252.	AT5G11260	BZIP56	bZIP	SbJAZ13	1190	1204	-	8.61E-07	0.0314	TCCGACGACGTGGCA
253.	AT5G15830	bZIP3	bZIP	SbJAZ13	1190	1204	-	4.76E-07	0.0336	TCCGACGACGTGGCA
254.	AT1G03840	IDD3	C2H2	SbJAZ11	1116	1135	+	1.67E-07	0.0127	AATAAAACGACAAAAGAAAT
255.	AT1G14580	IDD6	C2H2	SbJAZ11	1116	1135	-	3.16E-07	0.0241	ATTTCTTTTGTCGTTTTATT
256.	AT1G55110	IDD7	C2H2	SbJAZ11	1116	1133	+	2.20E-07	0.017	AATAAAACGACAAAAGAA
257.	AT1G72050	TFIIIA	C2H2	SbJAZ13	2162	2180	-	2.83E-10	1.12E-05	CTTCCTCCTCCTCCTCTGC
258.	AT1G72050	TFIIIA	C2H2	SbJAZ13	2165	2183	-	3.11E-10	1.12E-05	TCGCTTCCTCCTCCTCCTC
259.	AT1G72050	TFIIIA	C2H2	SbJAZ18	2160	2178	+	2.13E-09	5.12E-05	CTTCTTCTTCTTCCTCCTC
260.	AT1G72050	TFIIIA	C2H2	SbJAZ18	2163	2181	+	3.22E-09	5.81E-05	CTTCTTCTTCCTCCTCTTC
261.	AT1G72050	TFIIIA	C2H2	SbJAZ8	2022	2040	+	4.68E-09	6.25E-05	CCTGCCCCTCCTCCTCCGC
262.	AT1G72050	TFIIIA	C2H2	SbJAZ17	379	397	-	5.20E-09	6.25E-05	CCACCCCCTCCCCCTCCCC
263.	AT1G72050	TFIIIA	C2H2	SbJAZ13	2159	2177	-	7.74E-09	7.98E-05	CCTCCTCCTCCTCTGCTGC
264.	AT1G72050	TFIIIA	C2H2	SbJAZ1	230	248	-	1.06E-08	9.57E-05	CCCCCTCCCCCGCCACCTC
265.	AT1G72050	TFIIIA	C2H2	SbJAZ8	2025	2043	+	1.89E-08	1.51E-04	GCCCCTCCTCCTCCGCCCC
266.	AT1G72050	TFIIIA	C2H2	SbJAZ18	2157	2175	+	3.19E-08	2.30E-04	CCGCTTCTTCTTCTTCCTC
267.	AT1G72050	TFIIIA	C2H2	SbJAZ17	372	390	-	4.15E-08	2.72E-04	CTCCCCCTCCCCCCTCCTC
268.	AT1G72050	TFIIIA	C2H2	SbJAZ8	2028	2046	+	4.56E-08	0.000274	CCTCCTCCTCCGCCCCCAT
269.	AT1G72050	TFIIIA	C2H2	SbJAZ17	376	394	-	5.34E-08	0.000296	CCCCCTCCCCCTCCCCCCT
270.	AT1G72050	TFIIIA	C2H2	SbJAZ18	2169	2187	+	8.40E-08	0.000384	CTTCCTCCTCTTCTTTTTC
271.	AT1G72050	TFIIIA	C2H2	SbJAZ13	2168	2186	-	8.70E-08	0.000384	TCCTCGCTTCCTCCTCCTC
272.	AT1G72050	TFIIIA	C2H2	SbJAZ18	2166	2184	+	9.89E-08	0.000384	CTTCTTCCTCCTCTTCTTT
273.	AT1G72050	TFIIIA	C2H2	SbJAZ14	1857	1875	+	1.00E-07	0.000384	CCACCTCCTCCCCTCCCGC
274.	AT1G72050	TFIIIA	C2H2	SbJAZ1	227	245	-	1.00E-07	0.000384	CCTCCCCCGCCACCTCTTT
275.	AT1G72050	TFIIIA	C2H2	SbJAZ17	322	340	-	1.04E-07	0.000384	TCCCGTCCTCCCCCTCCCC
276.	AT1G72050	TFIIIA	C2H2	SbJAZ17	382	400	-	1.07E-07	0.000384	TCACCACCCCCTCCCCCTC
277.	AT1G72050	TFIIIA	C2H2	SbJAZ8	2150	2168	-	1.48E-07	0.000487	CTTGCTTCTCCTCCTTCTC
278.	AT1G72050	TFIIIA	C2H2	SbJAZ1	450	468	-	1.53E-07	0.000487	CCTCCTACTACTCCTCCTT
279.	AT1G72050	TFIIIA	C2H2	SbJAZ14	1851	1869	+	1.55E-07	0.000487	TCCTCCCCACCTCCTCCCC
280.	AT1G72050	TFIIIA	C2H2	SbJAZ17	325	343	-	1.74E-07	0.000524	CCCTCCCGTCCTCCCCCTC
281.	AT1G72050	TFIIIA	C2H2	SbJAZ8	2031	2049	+	2.30E-07	0.000649	CCTCCTCCGCCCCCATTTC
282.	AT1G72050	TFIIIA	C2H2	SbJAZ12	2099	2117	-	2.34E-07	0.000649	GCACCTCCTCGTCCTCCCC
283.	AT1G72050	TFIIIA	C2H2	SbJAZ12	2096	2114	-	2.51E-07	0.00067	CCTCCTCGTCCTCCCCCCT
284.	AT1G72050	TFIIIA	C2H2	SbJAZ11	1943	1961	-	2.60E-07	0.00067	ACTACCCCTCCTCCTCCTT
285.	AT1G72050	TFIIIA	C2H2	SbJAZ17	369	387	-	2.85E-07	0.00071	CCCCTCCCCCCTCCTCCCG
286.	AT1G72050	TFIIIA	C2H2	SbJAZ13	1824	1842	+	3.24E-07	0.000752	CCCCCTGCCCCTCCTCCGT
287.	AT1G72050	TFIIIA	C2H2	SbJAZ8	2019	2037	+	3.30E-07	0.000752	TGCCCTGCCCCTCCTCCTC
288.	AT1G72050	TFIIIA	C2H2	SbJAZ11	1937	1955	-	3.33E-07	0.000752	CCTCCTCCTCCTTGCTCTC
289.	AT1G72050	TFIIIA	C2H2	SbJAZ14	1854	1872	+	3.66E-07	0.000799	TCCCCACCTCCTCCCCTCC
290.	AT1G72050	TFIIIA	C2H2	SbJAZ18	2002	2020	+	3.76E-07	0.000799	CGCCATCCGCCTCCTCTCC

291.	AT1G72050	TFIIIA	C2H2	SbJAZ17	375	393	-	4.06E-07	0.000819	CCCCTCCCCCTCCCCCCTC
292.	AT1G72050	TFIIIA	C2H2	SbJAZ1	453	471	-	4.09E-07	0.000819	ATTCCTCCTACTACTCCTC
293.	AT1G72050	TFIIIA	C2H2	SbJAZ18	2008	2026	+	4.87E-07	0.00095	CCGCCTCCTCTCCTCCTTC
294.	AT1G72050	TFIIIA	C2H2	SbJAZ18	692	710	-	5.61E-07	0.00106	TCTTTCTCTCCTCCACCTC
295.	AT1G72050	TFIIIA	C2H2	SbJAZ6	1964	1982	+	6.45E-07	0.00119	CGGCCTCCCTCTCCTCCTG
296.	AT1G72050	TFIIIA	C2H2	SbJAZ7	2093	2111	-	7.13E-07	0.00129	TCTCCTCCTCTTTCCCCTT
297.	AT1G72050	TFIIIA	C2H2	SbJAZ1	456	474	-	8.09E-07	0.00142	CCTATTCCTCCTACTACTC
298.	AT1G72050	TFIIIA	C2H2	SbJAZ1	1233	1251	+	8.80E-07	0.00148	CCGCGACTACCTCCTCCCC
299.	AT1G72050	TFIIIA	C2H2	SbJAZ7	2096	2114	-	8.80E-07	0.00148	CGCTCTCCTCCTCTTTCCC
300.	AT1G72050	TFIIIA	C2H2	SbJAZ8	1926	1944	+	9.67E-07	0.00159	TCTCCCCCCCTCCCCCCAC
301.	AT2G02070	IDD5	C2H2	SbJAZ11	1116	1131	-	9.69E-07	0.0743	CTTTTGTCGTTTTATT
302.	AT2G02080	IDD5	C2H2	SbJAZ11	1116	1132	+	5.63E-07	0.0439	AATAAAACGACAAAAGA
303.	AT2G15740	IDD5	C2H2	SbJAZ10	2115	2125	-	4.55E-08	0.00348	GACGACGACGA
304.	AT3G13810	C2H2	C2H2	SbJAZ11	1117	1137	-	1.32E-07	0.0102	TTATTTCTTTTGTCGTTTTAT
305.	AT3G46070	C2H2	C2H2	SbJAZ18	911	925	+	9.45E-08	0.00737	TCTTCCTCACTCACC
306.	AT3G50700	C2H2	C2H2	SbJAZ11	1116	1130	+	1.63E-07	0.0127	AATAAAACGACAAAA
307.	AT5G03150	IDD10	C2H2	SbJAZ11	1116	1135	-	1.92E-07	0.0149	ATTTCTTTTGTCGTTTTATT
308.	AT5G04390	IDD10	C2H2	SbJAZ5	188	198	-	6.53E-07	0.051	GAGTGGAGGGG
309.	AT5G22990	C2H2	C2H2	SbJAZ10	2112	2126	+	1.58E-08	1.19E-03	ACGTCGTCGTCGTCG
310.	AT5G22990	C2H2	C2H2	SbJAZ12	251	265	-	8.92E-08	0.00335	TCGTCATCGATCTCC
311.	AT5G66730	C2H2	C2H2	SbJAZ11	1116	1132	+	2.26E-07	0.0176	AATAAAACGACAAAAGA
312.	AT3G12130	C3H	C3H	SbJAZ14	1352	1365	+	6.92E-07	0.054	AGGCAAAAAGTTAA
313.	AT5G63260	C3H	C3H	SbJAZ2	804	814	-	7.01E-07	0.0548	GAAAAAGTGAA
314.	AT2G22300	CAMTA3	CAMTA	SbJAZ18	2225	2233	-	2.08E-07	0.0115	CCGCGTCGC
315.	AT2G22300	CAMTA3	CAMTA	SbJAZ6	1872	1880	-	6.24E-07	0.0115	CCGCGTCGG
316.	AT2G22300	CAMTA3	CAMTA	SbJAZ6	1897	1905	-	6.24E-07	0.0115	CCGCGTCGG
317.	AT2G22300	CAMTA3	CAMTA	SbJAZ6	1958	1966	-	6.24E-07	0.0115	CCGCGTCGG
318.	AT4G16150	CMTA5	CAMTA	SbJAZ18	1423	1440	-	4.98E-09	3.59E-04	GCGCGTGTGTGGCGCGTT
319.	AT4G16150	CMTA5	CAMTA	SbJAZ2	1886	1903	-	3.76E-07	0.0135	ACGCGTTTTGGGCGTGTG
320.	AT4G16150	CMTA5	CAMTA	SbJAZ9	1883	1900	-	7.11E-07	0.0171	GCGAGGCAGGCGCGCGTG
321.	AT5G09410	CAMTA1	CAMTA	SbJAZ14	2075	2086	+	1.46E-07	0.00984	TAAGCGCGTGGG
322.	AT2G37590	Dof	Dof	SbJAZ14	494	508	+	4.17E-07	0.0325	ACTTTTTACTTTTTG
323.	AT3G50410	Dof	Dof	SbJAZ18	2166	2186	+	7.21E-07	0.0559	CTTCTTCCTCCTCTTCTTTTT
324.	AT4G38000	Dof	Dof	SbJAZ18	2160	2187	+	5.37E-08	0.00415	CTTCTTCTTCTTCCTCCTCTTCTTTTTC
325.	AT5G02460		Dof	SbJAZ18	2167	2187	-	7.49E-07	0.0582	GAAAAAGAAGAGGAGGAAGAA
326.	AT5G22220	E2F/DP	E2F/DP	SbJAZ14	2084	2093	-	5.25E-08	0.00316	GCGCGCCCCC
327.	AT5G22220	E2F/DP	E2F/DP	SbJAZ3	1789	1798	+	1.91E-07	0.00316	GCGCGCCAAC
328.	AT5G22220	E2F/DP	E2F/DP	SbJAZ11	1888	1897	-	2.09E-07	0.00316	GCGCGCCCCG
329.	AT5G22220	E2F/DP	E2F/DP	SbJAZ9	2018	2027	+	2.09E-07	0.00316	GCGCGCCCCG
330.	AT5G22220	E2F/DP	E2F/DP	SbJAZ3	1043	1052	+	9.03E-07	0.0109	GGGCGCCACG
331.	AT1G03800	ERF10	ERF	SbJAZ12	1384	1404	+	6.47E-09	2.54E-04	GCTGGACGCTCCGCCGCCGCA
332.	AT1G03800	ERF10	ERF	SbJAZ17	2024	2044	+	9.48E-09	2.54E-04	TCGCCCTTTCTCGCCGCCGCC
333.	AT1G03800	ERF10	ERF	SbJAZ2	1269	1289	+	1.21E-08	2.54E-04	CCCTTGCCAAGCGCCGCCGCC
334.	AT1G03800	ERF10	ERF	SbJAZ7	1940	1960	+	1.68E-08	2.54E-04	GTAGCTCTCCCCGCCGCCACG
335.	AT1G03800	ERF10	ERF	SbJAZ1	229	249	-	1.81E-08	2.54E-04	CCCCCCTCCCCCGCCACCTCT
336.	AT1G03800	ERF10	ERF	SbJAZ6	1922	1942	+	2.29E-08	2.68E-04	CGCCCCGACTCCACCGCCGGC
337.	AT1G03800	ERF10	ERF	SbJAZ18	1734	1754	-	6.49E-08	0.00065	CTCGCTGTTCGCGCCGCCGTG
338.	AT1G03800	ERF10	ERF	SbJAZ14	1967	1987	+	1.14E-07	0.000925	TCGCCCTCTACCTCCGCCAGC
339.	AT1G03800	ERF10	ERF	SbJAZ11	1820	1840	+	1.23E-07	0.000925	CCCGCACCGCCCGCCGCAGCG
340.	AT1G03800	ERF10	ERF	SbJAZ13	1849	1869	+	1.32E-07	0.000925	GATCACGTCGCCACCGCCACC
341.	AT1G03800	ERF10	ERF	SbJAZ13	1846	1866	+	1.51E-07	0.000964	TCCGATCACGTCGCCACCGCC
342.	AT1G03800	ERF10	ERF	SbJAZ12	1381	1401	+	2.40E-07	0.0014	CACGCTGGACGCTCCGCCGCC
343.	AT1G03800	ERF10	ERF	SbJAZ12	1558	1578	+	3.30E-07	0.00178	GCCGAGAGCGACGCCGCCGAT
344.	AT1G03800	ERF10	ERF	SbJAZ14	2225	2245	-	3.86E-07	0.00193	ACAAATCTCCGCGCCGCCGGC
345.	AT1G03800	ERF10	ERF	SbJAZ10	633	653	-	5.09E-07	0.00238	GCCGCCTCGATCGCCGTCTCG
346.	AT1G03800	ERF10	ERF	SbJAZ12	1356	1376	+	6.92E-07	0.00288	GAGCACCCGCCCGCCGCCTCC
347.	AT1G03800	ERF10	ERF	SbJAZ6	1849	1869	+	6.99E-07	0.00288	GTCAACACCGACGCCACCACG
348.	AT1G06160	ERF59	ERF	SbJAZ2	1280	1287	+	6.26E-07	0.00525	CGCCGCCG
349.	AT1G06160	ERF59	ERF	SbJAZ12	1395	1402	+	6.26E-07	0.00525	CGCCGCCG
350.	AT1G06160	ERF59	ERF	SbJAZ12	1569	1576	+	6.26E-07	0.00525	CGCCGCCG
351.	AT1G06160	ERF59	ERF	SbJAZ18	1736	1743	-	6.26E-07	0.00525	CGCCGCCG
352.	AT1G06160	ERF59	ERF	SbJAZ17	2035	2042	+	6.26E-07	0.00525	CGCCGCCG
353.	AT1G06160	ERF59	ERF	SbJAZ14	2227	2234	-	6.26E-07	0.00525	CGCCGCCG
354.	AT1G06160	ERF59	ERF	SbJAZ13	2302	2309	+	6.26E-07	0.00525	CGCCGCCG
355.	AT1G12630	ERF27	ERF	SbJAZ13	1848	1868	+	2.28E-07	0.0066	CGATCACGTCGCCACCGCCAC
356.	AT1G12630	ERF27	ERF	SbJAZ12	1383	1403	+	2.32E-07	0.0066	CGCTGGACGCTCCGCCGCCGC
357.	AT1G12630	ERF27	ERF	SbJAZ13	1252	1272	+	3.42E-07	0.0066	GACGTCGCCCGTTGCCGACAG
358.	AT1G12630	ERF27	ERF	SbJAZ6	1921	1941	+	4.28E-07	0.0066	ACGCCCCGACTCCACCGCCGG
359.	AT1G12630	ERF27	ERF	SbJAZ12	1402	1422	+	7.72E-07	0.00722	GCACACCCTTCCCACCGACAT
360.	AT1G12630	ERF27	ERF	SbJAZ17	2023	2043	+	8.86E-07	0.00722	CTCGCCCTTTCTCGCCGCCGC
361.	AT1G12630	ERF27	ERF	SbJAZ7	1939	1959	+	8.98E-07	0.00722	AGTAGCTCTCCCCGCCGCCAC
362.	AT1G12630	ERF27	ERF	SbJAZ12	1557	1577	+	9.36E-07	0.00722	CGCCGAGAGCGACGCCGCCGA
363.	AT1G19210	ERF17	ERF	SbJAZ1	2049	2062	-	5.70E-08	0.0037	GTGGCCGGTGGTGC
364.	AT1G19210	ERF17	ERF	SbJAZ3	478	491	+	3.77E-07	0.0122	ATGGACGGCGGATA
365.	AT1G19210	ERF17	ERF	SbJAZ17	2034	2047	-	8.21E-07	0.0151	AGGGGCGGCGGCGA

366.	AT1G19210	ERF17	ERF	SbJAZ17	341	354	+	9.32E-07	0.0151	GGGGGCGGCGGCAG
367.	AT1G21910	ERF012	ERF	SbJAZ18	1551	1570	+	1.21E-08	4.05E-04	GCCACGCGGGCTCCACCGCC
368.	AT1G21910	ERF012	ERF	SbJAZ6	1920	1939	+	1.43E-08	4.05E-04	GACGCCCCGACTCCACCGCC
369.	AT1G21910	ERF012	ERF	SbJAZ13	1847	1866	+	1.82E-08	4.05E-04	CCGATCACGTCGCCACCGCC
370.	AT1G21910	ERF012	ERF	SbJAZ11	1811	1830	+	8.53E-08	0.00142	GGGACGCCACCCGCACCGCC
371.	AT1G21910	ERF012	ERF	SbJAZ12	1401	1420	+	3.34E-07	0.00347	CGCACACCCTTCCCACCGAC
372.	AT1G21910	ERF012	ERF	SbJAZ9	1843	1862	+	3.55E-07	0.00347	CGCGCTCCCATCCCACCGCC
373.	AT1G21910	ERF012	ERF	SbJAZ8	2022	2041	+	3.64E-07	0.00347	CCTGCCCCTCCTCCTCCGCC
374.	AT1G21910	ERF012	ERF	SbJAZ18	1905	1924	+	4.88E-07	0.0037	CCGTGGCGGGGCCCACCGCC
375.	AT1G21910	ERF012	ERF	SbJAZ17	2025	2044	+	5.00E-07	0.0037	CGCCCTTTCTCGCCGCCGCC
376.	AT1G21910	ERF012	ERF	SbJAZ2	1270	1289	+	5.67E-07	0.00378	CCTTGCCAAGCGCCGCCGCC
377.	AT1G21910	ERF012	ERF	SbJAZ14	2210	2229	-	8.79E-07	0.00532	CCGGCTCCAACTCCTCCGCC
378.	AT1G21910	ERF012	ERF	SbJAZ10	636	655	-	9.83E-07	0.00546	CTGCCGCCTCGATCGCCGTC
379.	AT1G22810	ERF019	ERF	SbJAZ17	338	352	+	1.63E-08	9.73E-04	GGAGGGGGCGGCGGC
380.	AT1G22810	ERF019	ERF	SbJAZ13	1858	1872	-	2.67E-08	9.73E-04	TGGGGTGGCGGTGGC
381.	AT1G22810	ERF019	ERF	SbJAZ13	2300	2314	-	7.29E-08	0.00177	GGCCGCGGCGGCGCC
382.	AT1G22810	ERF019	ERF	SbJAZ12	1365	1379	-	1.65E-07	0.00301	GCCGGAGGCGGCGGG
383.	AT1G22810	ERF019	ERF	SbJAZ13	1855	1869	-	2.65E-07	0.00386	GGTGGCGGTGGCGAC
384.	AT1G22810	ERF019	ERF	SbJAZ12	1393	1407	-	3.95E-07	0.00479	GTGTGCGGCGGCGGA
385.	AT1G22810	ERF019	ERF	SbJAZ6	1852	1866	-	7.13E-07	0.00708	GGTGGCGTCGGTGTT
386.	AT1G22810	ERF019	ERF	SbJAZ6	433	447	+	7.77E-07	0.00708	GGATTTGGCGGTGCC
387.	AT1G28160	ERF087	ERF	SbJAZ2	1276	1290	-	5.10E-08	0.00286	GGGCGGCGGCGCTTG
388.	AT1G28160	ERF087	ERF	SbJAZ12	1391	1405	-	9.27E-08	0.00286	GTGCGGCGGCGGAGC
389.	AT1G28160	ERF087	ERF	SbJAZ12	1388	1402	-	1.66E-07	0.00286	CGGCGGCGGAGCGTC
390.	AT1G28160	ERF087	ERF	SbJAZ13	1693	1707	+	2.28E-07	0.00286	CCGCGGCGCCGGGAG
391.	AT1G28160	ERF087	ERF	SbJAZ17	343	357	+	2.47E-07	0.00286	GGGCGGCGGCAGAGC
392.	AT1G28160	ERF087	ERF	SbJAZ17	2031	2045	-	2.66E-07	0.00286	GGGCGGCGGCGAGAA
393.	AT1G28160	ERF087	ERF	SbJAZ7	1944	1958	-	3.44E-07	0.00315	TGGCGGCGGGGAGAG
394.	AT1G28160	ERF087	ERF	SbJAZ13	2298	2312	-	3.91E-07	0.00315	CCGCGGCGGCGCCCT
395.	AT1G28160	ERF087	ERF	SbJAZ12	1363	1377	-	5.29E-07	0.00378	CGGAGGCGGCGGGCG
396.	AT1G28160	ERF087	ERF	SbJAZ13	1853	1867	-	6.95E-07	0.00426	TGGCGGTGGCGACGT
397.	AT1G28160	ERF087	ERF	SbJAZ6	1926	1940	-	7.27E-07	0.00426	CGGCGGTGGAGTCGG
398.	AT1G28160	ERF087	ERF	SbJAZ14	2224	2238	+	9.37E-07	0.00503	AGCCGGCGGCGCGGA
399.	AT1G28360	ERF12	ERF	SbJAZ2	1280	1287	+	6.26E-07	0.00488	CGCCGCCG
400.	AT1G28360	ERF12	ERF	SbJAZ12	1395	1402	+	6.26E-07	0.00488	CGCCGCCG
401.	AT1G28360	ERF12	ERF	SbJAZ12	1569	1576	+	6.26E-07	0.00488	CGCCGCCG
402.	AT1G28360	ERF12	ERF	SbJAZ18	1736	1743	-	6.26E-07	0.00488	CGCCGCCG
403.	AT1G28360	ERF12	ERF	SbJAZ17	2035	2042	+	6.26E-07	0.00488	CGCCGCCG
404.	AT1G28360	ERF12	ERF	SbJAZ14	2227	2234	-	6.26E-07	0.00488	CGCCGCCG
405.	AT1G28360	ERF12	ERF	SbJAZ13	2302	2309	+	6.26E-07	0.00488	CGCCGCCG
406.	AT1G28370	ERF13	ERF	SbJAZ2	1276	1290	+	7.31E-08	0.00244	CAAGCGCCGCCGCCC
407.	AT1G28370	ERF13	ERF	SbJAZ12	1391	1405	+	7.60E-08	0.00244	GCTCCGCCGCCGCAC
408.	AT1G28370	ERF13	ERF	SbJAZ12	1388	1402	+	1.73E-07	0.00326	GACGCTCCGCCGCCG
409.	AT1G28370	ERF13	ERF	SbJAZ12	1363	1377	+	3.22E-07	0.00326	CGCCCGCCGCCTCCG
410.	AT1G28370	ERF13	ERF	SbJAZ17	343	357	-	3.31E-07	0.00326	GCTCTGCCGCCGCCC
411.	AT1G28370	ERF13	ERF	SbJAZ17	2031	2045	+	3.87E-07	0.00326	TTCTCGCCGCCGCCC
412.	AT1G28370	ERF13	ERF	SbJAZ13	1853	1867	+	4.07E-07	0.00326	ACGTCGCCACCGCCA
413.	AT1G28370	ERF13	ERF	SbJAZ13	1693	1707	-	4.07E-07	0.00326	CTCCCGGCGCCGCGG
414.	AT1G28370	ERF13	ERF	SbJAZ6	1926	1940	+	5.83E-07	0.00416	CCGACTCCACCGCCG
415.	AT1G28370	ERF13	ERF	SbJAZ7	1947	1961	+	8.23E-07	0.00528	TCCCCGCCGCCACGA
416.	AT1G28370	ERF13	ERF	SbJAZ14	2224	2238	-	9.69E-07	0.00565	TCCGCGCCGCCGGCT
417.	AT1G36060	ERF055	ERF	SbJAZ17	2034	2047	-	3.02E-08	1.32E-03	AGGGGCGGCGGCGA
418.	AT1G36060	ERF055	ERF	SbJAZ13	1856	1869	-	4.47E-08	0.00132	GGTGGCGGTGGCGA
419.	AT1G36060	ERF055	ERF	SbJAZ18	1560	1573	-	6.27E-08	0.00132	TGGGGCGGTGGAGC
420.	AT1G36060	ERF055	ERF	SbJAZ17	341	354	+	9.17E-08	0.00145	GGGGGCGGCGGCAG
421.	AT1G36060	ERF055	ERF	SbJAZ2	1279	1292	-	3.84E-07	0.00484	ACGGGCGGCGGCGC
422.	AT1G36060	ERF055	ERF	SbJAZ10	1844	1857	+	5.54E-07	0.00583	GTGGTCGGTCGCGG
423.	AT1G36060	ERF055	ERF	SbJAZ18	2186	2199	-	9.32E-07	0.00672	AGTGGCGGTGCCGA
424.	AT1G36060	ERF055	ERF	SbJAZ18	1914	1927	-	9.47E-07	0.00672	GTGGGCGGTGGGCC
425.	AT1G36060	ERF055	ERF	SbJAZ6	436	449	+	9.59E-07	0.00672	TTTGGCGGTGCCGA
426.	AT1G43160	ERF108	ERF	SbJAZ12	1391	1405	-	5.09E-08	0.00327	GTGCGGCGGCGGAGC
427.	AT1G43160	ERF108	ERF	SbJAZ2	1276	1290	-	1.44E-07	0.00443	GGGCGGCGGCGCTTG
428.	AT1G43160	ERF108	ERF	SbJAZ17	343	357	+	2.12E-07	0.00443	GGGCGGCGGCAGAGC
429.	AT1G43160	ERF108	ERF	SbJAZ13	1853	1867	-	3.52E-07	0.00443	TGGCGGTGGCGACGT
430.	AT1G43160	ERF108	ERF	SbJAZ7	1944	1958	-	3.77E-07	0.00443	TGGCGGCGGGGAGAG
431.	AT1G43160	ERF108	ERF	SbJAZ6	1926	1940	-	4.57E-07	0.00443	CGGCGGTGGAGTCGG
432.	AT1G43160	ERF108	ERF	SbJAZ13	1693	1707	+	5.34E-07	0.00443	CCGCGGCGCCGGGAG
433.	AT1G43160	ERF108	ERF	SbJAZ12	1388	1402	-	5.50E-07	0.00443	CGGCGGCGGAGCGTC
434.	AT1G43160	ERF108	ERF	SbJAZ12	1369	1383	-	6.47E-07	0.00463	GTGCGCCGGAGGCGG
435.	AT1G43160	ERF108	ERF	SbJAZ17	2031	2045	-	9.19E-07	0.00532	GGGCGGCGGCGAGAA
436.	AT1G43160	ERF108	ERF	SbJAZ12	1366	1380	-	9.27E-07	0.00532	CGCCGGAGGCGGCGG
437.	AT1G43160	ERF108	ERF	SbJAZ1	231	245	+	9.91E-07	0.00532	AGGTGGCGGGGGAGG
438.	AT1G44830	ERF014	ERF	SbJAZ6	1926	1940	+	6.16E-09	4.27E-04	CCGACTCCACCGCCG
439.	AT1G44830	ERF014	ERF	SbJAZ13	1853	1867	+	2.79E-08	9.65E-04	ACGTCGCCACCGCCA
440.	AT1G44830	ERF014	ERF	SbJAZ6	438	452	-	2.98E-07	0.00688	GCATCGGCACCGCCA

441.	AT1G44830	ERF014	ERF	SbJAZ9	1849	1863	+	9.41E-07	0.0137	CCCATCCCACCGCCA
442.	AT1G44830	ERF014	ERF	SbJAZ12	1366	1380	+	9.88E-07	0.0137	CCGCCGCCTCCGGCG
443.	AT1G46768	ERF006	ERF	SbJAZ13	1856	1868	-	1.66E-08	1.12E-03	GTGGCGGTGGCGA
444.	AT1G46768	ERF006	ERF	SbJAZ17	2034	2046	-	2.86E-07	0.00578	GGGGCGGCGGCGA
445.	AT1G46768	ERF006	ERF	SbJAZ18	2186	2198	-	3.05E-07	0.00578	GTGGCGGTGCCGA
446.	AT1G46768	ERF006	ERF	SbJAZ7	1947	1959	-	3.42E-07	0.00578	GTGGCGGCGGGGA
447.	AT1G46768	ERF006	ERF	SbJAZ12	1391	1403	-	5.68E-07	0.00634	GCGGCGGCGGAGC
448.	AT1G46768	ERF006	ERF	SbJAZ18	1560	1572	-	5.87E-07	0.00634	GGGGCGGTGGAGC
449.	AT1G46768	ERF006	ERF	SbJAZ17	342	354	+	6.55E-07	0.00634	GGGGCGGCGGCAG
450.	AT1G50640	ERF3	ERF	SbJAZ2	1276	1290	-	9.46E-08	0.00371	GGGCGGCGGCGCTTG
451.	AT1G50640	ERF3	ERF	SbJAZ12	1391	1405	-	1.56E-07	0.00371	GTGCGGCGGCGGAGC
452.	AT1G50640	ERF3	ERF	SbJAZ13	1853	1867	-	2.38E-07	0.00371	TGGCGGTGGCGACGT
453.	AT1G50640	ERF3	ERF	SbJAZ12	1388	1402	-	2.46E-07	0.00371	CGGCGGCGGAGCGTC
454.	AT1G50640	ERF3	ERF	SbJAZ6	1926	1940	-	2.73E-07	0.00371	CGGCGGTGGAGTCGG
455.	AT1G50640	ERF3	ERF	SbJAZ7	1944	1958	-	4.36E-07	0.00402	TGGCGGCGGGGAGAG
456.	AT1G50640	ERF3	ERF	SbJAZ13	1693	1707	+	5.29E-07	0.00402	CCGCGGCGCCGGGAG
457.	AT1G50640	ERF3	ERF	SbJAZ12	1363	1377	-	5.34E-07	0.00402	CGGAGGCGGCGGGCG
458.	AT1G50640	ERF3	ERF	SbJAZ17	2031	2045	-	5.52E-07	0.00402	GGGCGGCGGCGAGAA
459.	AT1G50640	ERF3	ERF	SbJAZ17	343	357	+	5.91E-07	0.00402	GGGCGGCGGCAGAGC
460.	AT1G50640	ERF3	ERF	SbJAZ13	1125	1139	-	9.63E-07	0.00595	CGACGGCGATGGCTG
461.	AT1G53170	ERF079	ERF	SbJAZ12	1390	1404	+	4.55E-09	3.12E-04	CGCTCCGCCGCCGCA
462.	AT1G53170	ERF079	ERF	SbJAZ2	1275	1289	+	1.98E-08	6.79E-04	CCAAGCGCCGCCGCC
463.	AT1G53170	ERF079	ERF	SbJAZ13	1855	1869	+	9.62E-08	0.00193	GTCGCCACCGCCACC
464.	AT1G53170	ERF079	ERF	SbJAZ17	344	358	-	1.20E-07	0.00193	AGCTCTGCCGCCGCC
465.	AT1G53170	ERF079	ERF	SbJAZ17	2030	2044	+	1.41E-07	0.00193	TTTCTCGCCGCCGCC
466.	AT1G53170	ERF079	ERF	SbJAZ14	2225	2239	-	2.12E-07	0.00243	CTCCGCGCCGCCGGC
467.	AT1G53170	ERF079	ERF	SbJAZ6	1928	1942	+	2.72E-07	0.00266	GACTCCACCGCCGGC
468.	AT1G53170	ERF079	ERF	SbJAZ7	1946	1960	+	3.37E-07	0.00289	CTCCCCGCCGCCACG
469.	AT1G53170	ERF079	ERF	SbJAZ12	1387	1401	+	4.33E-07	0.0033	GGACGCTCCGCCGCC
470.	AT1G53170	ERF079	ERF	SbJAZ8	2027	2041	+	6.05E-07	0.00416	CCCTCCTCCTCCGCC
471.	AT1G53170	ERF079	ERF	SbJAZ12	1365	1379	+	7.94E-07	0.0048	CCCGCCGCCTCCGGC
472.	AT1G53170	ERF079	ERF	SbJAZ13	1694	1708	-	8.40E-07	0.0048	TCTCCCGGCGCCGCG
473.	AT1G53910	ERF074	ERF	SbJAZ13	1684	1704	+	2.06E-08	6.43E-04	AGGCGGGGACCGCGGCGCCGG
474.	AT1G53910	ERF074	ERF	SbJAZ12	1391	1411	-	3.16E-08	6.43E-04	AAGGGTGTGCGGCGGCGGAGC
475.	AT1G53910	ERF074	ERF	SbJAZ12	1363	1383	-	3.67E-08	6.43E-04	GTGCGCCGGAGGCGGCGGGCG
476.	AT1G53910	ERF074	ERF	SbJAZ17	337	357	+	3.85E-08	6.43E-04	GGGAGGGGGCGGCGGCAGAGC
477.	AT1G53910	ERF074	ERF	SbJAZ6	429	449	+	5.45E-08	0.000727	CGGCGGATTTGGCGGTGCCGA
478.	AT1G53910	ERF074	ERF	SbJAZ17	334	354	+	1.37E-07	0.00149	GACGGGAGGGGGCGGCGGCAG
479.	AT1G53910	ERF074	ERF	SbJAZ13	1856	1876	-	1.56E-07	0.00149	GCGCTGGGGTGGCGGTGGCGA
480.	AT1G53910	ERF074	ERF	SbJAZ14	2218	2238	+	1.96E-07	0.00157	AGTTGGAGCCGGCGGCGCGGA
481.	AT1G53910	ERF074	ERF	SbJAZ12	1565	1585	-	2.11E-07	0.00157	TGACCGGATCGGCGGCGTCGC
482.	AT1G53910	ERF074	ERF	SbJAZ12	1394	1414	-	4.15E-07	0.00277	GGGAAGGGTGTGCGGCGGCGG
483.	AT1G53910	ERF074	ERF	SbJAZ10	638	658	+	5.83E-07	0.00337	CGGCGATCGAGGCGGCAGCGC
484.	AT1G53910	ERF074	ERF	SbJAZ5	1720	1740	-	6.06E-07	0.00337	GGGGTGGTTCGGCGGCTGCCA
485.	AT1G53910	ERF074	ERF	SbJAZ17	2034	2054	-	8.55E-07	0.0044	CAAACAGAGGGGCGGCGGCGA
486.	AT1G68550	ERF118	ERF	SbJAZ12	1391	1405	+	3.22E-08	2.12E-03	GCTCCGCCGCCGCAC
487.	AT1G68550	ERF118	ERF	SbJAZ2	1276	1290	+	6.98E-08	0.00213	CAAGCGCCGCCGCCC
488.	AT1G68550	ERF118	ERF	SbJAZ6	1926	1940	+	1.44E-07	0.00213	CCGACTCCACCGCCG
489.	AT1G68550	ERF118	ERF	SbJAZ13	1693	1707	-	1.70E-07	0.00213	CTCCCGGCGCCGCGG
490.	AT1G68550	ERF118	ERF	SbJAZ17	343	357	-	1.76E-07	0.00213	GCTCTGCCGCCGCCC
491.	AT1G68550	ERF118	ERF	SbJAZ12	1363	1377	+	2.11E-07	0.00213	CGCCCGCCGCCTCCG
492.	AT1G68550	ERF118	ERF	SbJAZ13	1853	1867	+	2.27E-07	0.00213	ACGTCGCCACCGCCA
493.	AT1G68550	ERF118	ERF	SbJAZ12	1388	1402	+	2.79E-07	0.0023	GACGCTCCGCCGCCG
494.	AT1G68550	ERF118	ERF	SbJAZ17	2031	2045	+	3.36E-07	0.00245	TTCTCGCCGCCGCCC
495.	AT1G68550	ERF118	ERF	SbJAZ13	2298	2312	+	3.71E-07	0.00245	AGGGCGCCGCCGCGG
496.	AT1G68550	ERF118	ERF	SbJAZ7	1947	1961	+	4.47E-07	0.00268	TCCCCGCCGCCACGA
497.	AT1G68550	ERF118	ERF	SbJAZ2	2221	2235	-	5.70E-07	0.00313	TCGCTACCGCCGCGG
498.	AT1G68550	ERF118	ERF	SbJAZ7	1944	1958	+	7.17E-07	0.00357	CTCTCCCCGCCGCCA
499.	AT1G68550	ERF118	ERF	SbJAZ13	1856	1870	+	7.58E-07	0.00357	TCGCCACCGCCACCC
500.	AT1G71450	ERF021	ERF	SbJAZ8	2026	2045	+	9.43E-09	6.68E-04	CCCCTCCTCCTCCGCCCCCA
501.	AT1G71450	ERF021	ERF	SbJAZ17	79	98	+	5.63E-08	0.00176	CCCCGTCCCCGTCCCCGCCA
502.	AT1G71450	ERF021	ERF	SbJAZ6	1924	1943	+	7.45E-08	0.00176	CCCCGACTCCACCGCCGGCC
503.	AT1G71450	ERF021	ERF	SbJAZ10	635	654	-	1.17E-07	0.00199	TGCCGCCTCGATCGCCGTCT
504.	AT1G71450	ERF021	ERF	SbJAZ6	429	448	-	1.71E-07	0.00199	CGGCACCGCCAAATCCGCCG
505.	AT1G71450	ERF021	ERF	SbJAZ13	1684	1703	-	2.08E-07	0.00199	CGGCGCCGCGGTCCCCGCCT
506.	AT1G71450	ERF021	ERF	SbJAZ13	1854	1873	+	2.25E-07	0.00199	CGTCGCCACCGCCACCCCAG
507.	AT1G71450	ERF021	ERF	SbJAZ14	2218	2237	-	2.38E-07	0.00199	CCGCGCCGCCGGCTCCAACT
508.	AT1G71450	ERF021	ERF	SbJAZ14	2215	2234	-	2.60E-07	0.00199	CGCCGCCGGCTCCAACTCCT
509.	AT1G71450	ERF021	ERF	SbJAZ12	1557	1576	+	2.83E-07	0.00199	CGCCGAGAGCGACGCCGCCG
510.	AT1G71450	ERF021	ERF	SbJAZ14	2209	2228	-	3.29E-07	0.00199	CGGCTCCAACTCCTCCGCCC
511.	AT1G71450	ERF021	ERF	SbJAZ10	638	657	-	3.36E-07	0.00199	CGCTGCCGCCTCGATCGCCG
512.	AT1G71450	ERF021	ERF	SbJAZ12	1554	1573	+	4.21E-07	0.00229	CCCCGCCGAGAGCGACGCCG
513.	AT1G71450	ERF021	ERF	SbJAZ6	426	445	-	4.63E-07	0.00234	CACCGCCAAATCCGCCGATG
514.	AT1G71450	ERF021	ERF	SbJAZ1	228	247	-	5.60E-07	0.00264	CCCCTCCCCCGCCACCTCTT
515.	AT1G71450	ERF021	ERF	SbJAZ13	1681	1700	-	6.02E-07	0.00267	CGCCGCGGTCCCCGCCTCCC

516.	AT1G71450	ERF021	ERF	SbJAZ13	1672	1691	-	7.96E-07	0.00316	CCCCGCCTCCCCGGCGGCCG
517.	AT1G71450	ERF021	ERF	SbJAZ18	1896	1915	-	8.73E-07	0.00316	CCCCGCCACGGCGACCGGCT
518.	AT1G71450	ERF021	ERF	SbJAZ14	2212	2231	-	8.82E-07	0.00316	CGCCGGCTCCAACTCCTCCG
519.	AT1G71450	ERF021	ERF	SbJAZ6	1962	1981	+	8.91E-07	0.00316	CGCGGCCTCCCTCTCCTCCT
520.	AT1G71450	ERF021	ERF	SbJAZ6	1901	1920	+	9.86E-07	0.00333	CGCGGCCTCCCTCTCCCCCG
521.	AT1G72360	ERF73	ERF	SbJAZ12	1388	1408	+	4.49E-08	0.00126	GACGCTCCGCCGCCGCACACC
522.	AT1G72360	ERF73	ERF	SbJAZ13	1687	1707	-	4.58E-08	0.00126	CTCCCGGCGCCGCGGTCCCCG
523.	AT1G72360	ERF73	ERF	SbJAZ13	1853	1873	+	5.52E-08	0.00126	ACGTCGCCACCGCCACCCCAG
524.	AT1G72360	ERF73	ERF	SbJAZ12	1360	1380	+	8.50E-08	0.00146	ACCCGCCCGCCGCCTCCGGCG
525.	AT1G72360	ERF73	ERF	SbJAZ12	1363	1383	+	1.34E-07	0.00151	CGCCCGCCGCCTCCGGCGCAC
526.	AT1G72360	ERF73	ERF	SbJAZ14	2221	2241	-	1.51E-07	0.00151	ATCTCCGCGCCGCCGGCTCCA
527.	AT1G72360	ERF73	ERF	SbJAZ7	1944	1964	+	1.54E-07	0.00151	CTCTCCCCGCCGCCACGAGAA
528.	AT1G72360	ERF73	ERF	SbJAZ2	2215	2235	-	2.97E-07	0.00231	TCGCTACCGCCGCGGGCGCGG
529.	AT1G72360	ERF73	ERF	SbJAZ17	2028	2048	+	3.02E-07	0.00231	CCTTTCTCGCCGCCGCCCCTC
530.	AT1G72360	ERF73	ERF	SbJAZ6	432	452	-	3.37E-07	0.00231	GCATCGGCACCGCCAAATCCG
531.	AT1G72360	ERF73	ERF	SbJAZ6	1926	1946	+	4.60E-07	0.0027	CCGACTCCACCGCCGGCCGGG
532.	AT1G72360	ERF73	ERF	SbJAZ18	1730	1750	-	4.72E-07	0.0027	CTGTTCGCGCCGCCGTGGCGT
533.	AT1G72360	ERF73	ERF	SbJAZ1	225	245	-	6.37E-07	0.00336	CCTCCCCCGCCACCTCTTTTC
534.	AT1G72360	ERF73	ERF	SbJAZ2	1273	1293	+	9.17E-07	0.00449	TGCCAAGCGCCGCCGCCCGTC
535.	AT1G75490	ERF049	ERF	SbJAZ6	1926	1940	+	8.55E-09	5.93E-04	CCGACTCCACCGCCG
536.	AT1G75490	ERF049	ERF	SbJAZ13	1853	1867	+	3.11E-08	1.08E-03	ACGTCGCCACCGCCA
537.	AT1G75490	ERF049	ERF	SbJAZ2	1276	1290	+	3.23E-07	0.00726	CAAGCGCCGCCGCCC
538.	AT1G75490	ERF049	ERF	SbJAZ18	1557	1571	+	4.55E-07	0.00726	CGGGCTCCACCGCCC
539.	AT1G75490	ERF049	ERF	SbJAZ17	343	357	-	5.24E-07	0.00726	GCTCTGCCGCCGCCC
540.	AT1G75490	ERF049	ERF	SbJAZ12	1388	1402	+	8.77E-07	0.00981	GACGCTCCGCCGCCG
541.	AT1G75490	ERF049	ERF	SbJAZ6	438	452	-	9.90E-07	0.00981	GCATCGGCACCGCCA
542.	AT1G77640	ERF013	ERF	SbJAZ13	1858	1878	-	9.75E-09	6.41E-04	CCGCGCTGGGGTGGCGGTGGC
543.	AT1G77640	ERF013	ERF	SbJAZ6	1852	1872	-	2.23E-08	7.32E-04	GGGCGTGGTGGCGTCGGTGTT
544.	AT1G77640	ERF013	ERF	SbJAZ6	427	447	+	1.32E-07	0.0029	ATCGGCGGATTTGGCGGTGCC
545.	AT1G77640	ERF013	ERF	SbJAZ6	1931	1951	-	4.59E-07	0.00625	GTGGCCCCGGCCGGCGGTGGA
546.	AT1G77640	ERF013	ERF	SbJAZ9	1854	1874	-	4.76E-07	0.00625	GGTTGGCGAACTGGCGGTGGG
547.	AT1G77640	ERF013	ERF	SbJAZ12	1365	1385	-	8.36E-07	0.00915	GCGTGCGCCGGAGGCGGCGGG
548.	AT1G78080	ERF059	ERF	SbJAZ18	1918	1927	+	4.14E-07	0.0248	CACCGCCCAC
549.	AT1G78080	ERF059	ERF	SbJAZ17	341	350	-	7.25E-07	0.0248	CGCCGCCCCC
550.	AT2G31230	ERF093	ERF	SbJAZ12	1389	1403	+	2.31E-08	1.65E-03	ACGCTCCGCCGCCGC
551.	AT2G31230	ERF093	ERF	SbJAZ13	1854	1868	+	8.47E-08	0.00236	CGTCGCCACCGCCAC
552.	AT2G31230	ERF093	ERF	SbJAZ2	1274	1288	+	1.12E-07	0.00236	GCCAAGCGCCGCCGC
553.	AT2G31230	ERF093	ERF	SbJAZ7	1945	1959	+	1.32E-07	0.00236	TCTCCCCGCCGCCAC
554.	AT2G31230	ERF093	ERF	SbJAZ17	2029	2043	+	2.24E-07	0.00321	CTTTCTCGCCGCCGC
555.	AT2G31230	ERF093	ERF	SbJAZ12	1361	1375	+	6.17E-07	0.00737	CCCGCCCGCCGCCTC
556.	AT2G31230	ERF093	ERF	SbJAZ6	1927	1941	+	8.07E-07	0.00825	CGACTCCACCGCCGG
557.	AT2G33710	ERF112	ERF	SbJAZ12	1391	1405	+	8.37E-08	0.00432	GCTCCGCCGCCGCAC
558.	AT2G33710	ERF112	ERF	SbJAZ2	1276	1290	+	2.02E-07	0.00432	CAAGCGCCGCCGCCC
559.	AT2G33710	ERF112	ERF	SbJAZ13	1693	1707	-	2.79E-07	0.00432	CTCCCGGCGCCGCGG
560.	AT2G33710	ERF112	ERF	SbJAZ13	1853	1867	+	2.84E-07	0.00432	ACGTCGCCACCGCCA
561.	AT2G33710	ERF112	ERF	SbJAZ6	1926	1940	+	3.44E-07	0.00432	CCGACTCCACCGCCG
562.	AT2G33710	ERF112	ERF	SbJAZ12	1388	1402	+	4.33E-07	0.00443	GACGCTCCGCCGCCG
563.	AT2G33710	ERF112	ERF	SbJAZ17	343	357	-	4.94E-07	0.00443	GCTCTGCCGCCGCCC
564.	AT2G33710	ERF112	ERF	SbJAZ7	1944	1958	+	7.01E-07	0.00518	CTCTCCCCGCCGCCA
565.	AT2G33710	ERF112	ERF	SbJAZ12	1366	1380	+	7.43E-07	0.00518	CCGCCGCCTCCGGCG
566.	AT2G35700	ERF038	ERF	SbJAZ13	1853	1867	-	1.73E-07	0.00627	TGGCGGTGGCGACGT
567.	AT2G35700	ERF038	ERF	SbJAZ6	1926	1940	-	2.03E-07	0.00627	CGGCGGTGGAGTCGG
568.	AT2G35700	ERF038	ERF	SbJAZ12	1407	1421	-	2.75E-07	0.00627	TGTCGGTGGGAAGGG
569.	AT2G38340	ERF046	ERF	SbJAZ13	1854	1870	+	7.84E-07	0.053	CGTCGCCACCGCCACCC
570.	AT2G40220	ERF052	ERF	SbJAZ8	2079	2088	+	1.76E-08	9.67E-04	CGGCGCCCCC
571.	AT2G40220	ERF052	ERF	SbJAZ17	341	350	-	3.52E-08	9.67E-04	CGCCGCCCCC
572.	AT2G40220	ERF052	ERF	SbJAZ2	1280	1289	+	1.40E-07	0.00192	CGCCGCCGCC
573.	AT2G40220	ERF052	ERF	SbJAZ17	2035	2044	+	1.40E-07	0.00192	CGCCGCCGCC
574.	AT2G40220	ERF052	ERF	SbJAZ10	648	657	-	3.67E-07	0.00336	CGCTGCCGCC
575.	AT2G40220	ERF052	ERF	SbJAZ9	2139	2148	+	4.54E-07	0.00336	CGGCGCCCGC
576.	AT2G40220	ERF052	ERF	SbJAZ12	1600	1609	+	4.89E-07	0.00336	CGCCGCCCCT
577.	AT2G40220	ERF052	ERF	SbJAZ17	2038	2047	+	4.89E-07	0.00336	CGCCGCCCCT
578.	AT2G40220	ERF052	ERF	SbJAZ12	1367	1376	+	5.77E-07	0.00352	CGCCGCCTCC
579.	AT2G40220	ERF052	ERF	SbJAZ13	1694	1703	-	6.98E-07	0.00384	CGGCGCCGCG
580.	AT2G40220	ERF052	ERF	SbJAZ10	749	758	-	8.89E-07	0.00444	CGGTGCCTCC
581.	AT2G40340	ERF048	ERF	SbJAZ14	2216	2236	+	3.58E-08	8.69E-04	GGAGTTGGAGCCGGCGGCGCG
582.	AT2G40340	ERF048	ERF	SbJAZ17	381	401	+	3.92E-08	8.69E-04	GGAGGGGGAGGGGGTGGTGAG
583.	AT2G40340	ERF048	ERF	SbJAZ12	1365	1385	-	4.92E-08	0.000869	GCGTGCGCCGGAGGCGGCGGG
584.	AT2G40340	ERF048	ERF	SbJAZ8	2030	2050	-	4.92E-08	0.000869	GGAAATGGGGGCGGAGGAGGA
585.	AT2G40340	ERF048	ERF	SbJAZ13	1855	1875	-	8.74E-08	0.00123	CGCTGGGGTGGCGGTGGCGAC
586.	AT2G40340	ERF048	ERF	SbJAZ12	1393	1413	-	1.47E-07	0.00141	GGAAGGGTGTGCGGCGGCGGA
587.	AT2G40340	ERF048	ERF	SbJAZ13	1682	1702	+	1.64E-07	0.00141	GGAGGCGGGGACCGCGGCGCC
588.	AT2G40340	ERF048	ERF	SbJAZ14	2145	2165	-	1.73E-07	0.00141	CGCAGCTGCTGCGGTGGTTGC
589.	AT2G40340	ERF048	ERF	SbJAZ6	427	447	+	1.98E-07	0.00141	ATCGGCGGATTTGGCGGTGCC
590.	AT2G40340	ERF048	ERF	SbJAZ14	1856	1876	-	2.00E-07	0.00141	GGCGGGAGGGGAGGAGGTGGG

591.	AT2G40340	ERF048	ERF	SbJAZ13	2158	2178	+	5.71E-07	0.00366	AGCAGCAGAGGAGGAGGAGGA
592.	AT2G40340	ERF048	ERF	SbJAZ12	1555	1575	-	6.88E-07	0.00376	GGCGGCGTCGCTCTCGGCGGG
593.	AT2G40340	ERF048	ERF	SbJAZ6	1849	1869	-	8.06E-07	0.00376	CGTGGTGGCGTCGGTGTTGAC
594.	AT2G40340	ERF048	ERF	SbJAZ17	332	352	+	8.69E-07	0.00376	AGGACGGGAGGGGGCGGCGGC
595.	AT2G40340	ERF048	ERF	SbJAZ8	2027	2047	-	8.76E-07	0.00376	AATGGGGGCGGAGGAGGAGGG
596.	AT2G40340	ERF048	ERF	SbJAZ10	1867	1887	-	8.83E-07	0.00376	CGCGATGGAGCTGGAGGTGCG
597.	AT2G40340	ERF048	ERF	SbJAZ14	2148	2168	-	9.06E-07	0.00376	GGTCGCAGCTGCTGCGGTGGT
598.	AT2G44840	FAR1	ERF	SbJAZ12	1392	1406	+	3.15E-07	0.0105	CTCCGCCGCCGCACA
599.	AT2G44840	FAR1	ERF	SbJAZ7	1948	1962	+	3.82E-07	0.0105	CCCCGCCGCCACGAG
600.	AT2G44840	FAR1	ERF	SbJAZ14	2223	2237	-	5.67E-07	0.0105	CCGCGCCGCCGGCTC
601.	AT2G44840	GATA	ERF	SbJAZ13	2299	2313	+	6.44E-07	0.0105	GGGCGCCGCCGCGGC
602.	AT2G44840	GATA	ERF	SbJAZ18	1732	1746	-	8.03E-07	0.0105	TCGCGCCGCCGTGGC
603.	AT2G44840	GeBP	ERF	SbJAZ12	1364	1378	+	9.73E-07	0.0106	GCCCGCCGCCTCCGG
604.	AT3G11020	ERF044	ERF	SbJAZ13	1853	1873	-	1.70E-08	1.17E-03	CTGGGGTGGCGGTGGCGACGT
605.	AT3G11020	ERF044	ERF	SbJAZ18	1557	1577	-	2.24E-07	0.00649	TCGGTGGGGCGGTGGAGCCCG
606.	AT3G11020	ERF044	ERF	SbJAZ12	1360	1380	-	2.83E-07	0.00649	CGCCGGAGGCGGCGGGCGGGT
607.	AT3G11020	ERF044	ERF	SbJAZ17	337	357	+	4.12E-07	0.00708	GGGAGGGGGCGGCGGCAGAGC
608.	AT3G11020	ERF044	ERF	SbJAZ6	432	452	+	5.73E-07	0.00788	CGGATTTGGCGGTGCCGATGC
609.	AT3G15210	ERF078	ERF	SbJAZ12	1391	1405	+	1.76E-08	1.23E-03	GCTCCGCCGCCGCAC
610.	AT3G15210	ERF078	ERF	SbJAZ7	1947	1961	+	1.46E-07	0.00509	TCCCCGCCGCCACGA
611.	AT3G15210	ERF078	ERF	SbJAZ2	1276	1290	+	3.64E-07	0.00638	CAAGCGCCGCCGCCC
612.	AT3G15210	ERF078	ERF	SbJAZ12	1363	1377	+	4.41E-07	0.00638	CGCCCGCCGCCTCCG
613.	AT3G15210	ERF078	ERF	SbJAZ14	2224	2238	-	5.52E-07	0.00638	TCCGCGCCGCCGGCT
614.	AT3G15210	ERF078	ERF	SbJAZ17	2034	2048	+	5.92E-07	0.00638	TCGCCGCCGCCCCTC
615.	AT3G15210	ERF078	ERF	SbJAZ17	340	354	-	6.40E-07	0.00638	CTGCCGCCGCCCCCT
616.	AT3G16770	ERF072	ERF	SbJAZ2	1279	1286	+	6.26E-07	0.00909	GCGCCGCC
617.	AT3G16770	ERF072	ERF	SbJAZ18	1737	1744	-	6.26E-07	0.00909	GCGCCGCC
618.	AT3G16770	ERF072	ERF	SbJAZ14	2228	2235	-	6.26E-07	0.00909	GCGCCGCC
619.	AT3G16770	ERF072	ERF	SbJAZ13	2301	2308	+	6.26E-07	0.00909	GCGCCGCC
620.	AT3G20310	ERF083	ERF	SbJAZ17	81	101	+	1.57E-08	6.67E-04	CCGTCCCCGTCCCCGCCATCG
621.	AT3G20310	ERF083	ERF	SbJAZ13	1847	1867	+	3.00E-08	6.67E-04	CCGATCACGTCGCCACCGCCA
622.	AT3G20310	ERF083	ERF	SbJAZ17	2025	2045	+	3.11E-08	6.67E-04	CGCCCTTTCTCGCCGCCGCCC
623.	AT3G20310	ERF083	ERF	SbJAZ8	2022	2042	+	3.73E-08	6.67E-04	CCTGCCCCTCCTCCTCCGCCC
624.	AT3G20310	ERF083	ERF	SbJAZ12	1556	1576	+	5.39E-08	0.000771	CCGCCGAGAGCGACGCCGCCG
625.	AT3G20310	ERF083	ERF	SbJAZ10	635	655	-	7.72E-08	0.000772	CTGCCGCCTCGATCGCCGTCT
626.	AT3G20310	ERF083	ERF	SbJAZ2	1270	1290	+	9.17E-08	0.000772	CCTTGCCAAGCGCCGCCGCCC
627.	AT3G20310	ERF083	ERF	SbJAZ13	1824	1844	+	9.28E-08	0.000772	CCCCCTGCCCCTCCTCCGTCC
628.	AT3G20310	ERF083	ERF	SbJAZ12	1382	1402	+	9.71E-08	0.000772	ACGCTGGACGCTCCGCCGCCG
629.	AT3G20310	ERF083	ERF	SbJAZ6	1923	1943	+	1.11E-07	0.000796	GCCCCGACTCCACCGCCGGCC
630.	AT3G20310	ERF083	ERF	SbJAZ8	1933	1953	+	1.64E-07	0.00107	CCCTCCCCCCACCCGCCGGCG
631.	AT3G20310	ERF083	ERF	SbJAZ7	1938	1958	+	1.93E-07	0.00115	CAGTAGCTCTCCCCGCCGCCA
632.	AT3G20310	ERF083	ERF	SbJAZ1	231	251	-	2.09E-07	0.00115	AACCCCCCTCCCCCGCCACCT
633.	AT3G20310	ERF083	ERF	SbJAZ18	1733	1753	-	2.45E-07	0.00125	TCGCTGTTCGCGCCGCCGTGG
634.	AT3G20310	ERF083	ERF	SbJAZ12	1360	1380	+	2.85E-07	0.00134	ACCCGCCCGCCGCCTCCGGCG
635.	AT3G20310	ERF083	ERF	SbJAZ1	228	248	-	3.00E-07	0.00134	CCCCCTCCCCCGCCACCTCTT
636.	AT3G20310	ERF083	ERF	SbJAZ14	1968	1988	+	3.82E-07	0.00159	CGCCCTCTACCTCCGCCAGCG
637.	AT3G20310	ERF083	ERF	SbJAZ13	1693	1713	-	3.98E-07	0.00159	TCTCATCTCCCGGCGCCGCGG
638.	AT3G20310	ERF083	ERF	SbJAZ6	1906	1926	+	4.51E-07	0.0017	CCTCCCTCTCCCCCGACGCCC
639.	AT3G20310	ERF083	ERF	SbJAZ14	2209	2229	-	4.75E-07	0.0017	CCGGCTCCAACTCCTCCGCCC
640.	AT3G20310	ERF083	ERF	SbJAZ17	343	363	-	5.32E-07	0.00177	CTGAGAGCTCTGCCGCCGCCC
641.	AT3G20310	ERF083	ERF	SbJAZ12	1385	1405	+	5.71E-07	0.00177	CTGGACGCTCCGCCGCCGCAC
642.	AT3G20310	ERF083	ERF	SbJAZ17	2022	2042	+	5.95E-07	0.00177	CCTCGCCCTTTCTCGCCGCCG
643.	AT3G20310	ERF083	ERF	SbJAZ14	1965	1985	+	6.07E-07	0.00177	ACTCGCCCTCTACCTCCGCCA
644.	AT3G20310	ERF083	ERF	SbJAZ7	1941	1961	+	6.19E-07	0.00177	TAGCTCTCCCCGCCGCCACGA
645.	AT3G20310	ERF083	ERF	SbJAZ11	1818	1838	+	7.49E-07	0.00199	CACCCGCACCGCCCGCCGCAG
646.	AT3G20310	ERF083	ERF	SbJAZ13	1850	1870	+	7.64E-07	0.00199	ATCACGTCGCCACCGCCACCC
647.	AT3G20310	ERF083	ERF	SbJAZ5	1821	1841	+	7.80E-07	0.00199	CACTCGCACCGGGCGCCGTCA
648.	AT3G20310	ERF083	ERF	SbJAZ6	1920	1940	+	8.53E-07	0.00211	GACGCCCCGACTCCACCGCCG
649.	AT3G23220	ERF095	ERF	SbJAZ12	1391	1405	-	2.44E-08	1.18E-03	GTGCGGCGGCGGAGC
650.	AT3G23220	ERF095	ERF	SbJAZ12	1363	1377	-	6.03E-08	0.00118	CGGAGGCGGCGGGCG
651.	AT3G23220	ERF095	ERF	SbJAZ7	1947	1961	-	7.12E-08	0.00118	TCGTGGCGGCGGGGA
652.	AT3G23220	ERF095	ERF	SbJAZ2	1276	1290	-	7.35E-08	0.00118	GGGCGGCGGCGCTTG
653.	AT3G23220	ERF095	ERF	SbJAZ13	1856	1870	-	1.23E-07	0.00158	GGGTGGCGGTGGCGA
654.	AT3G23220	ERF095	ERF	SbJAZ12	1366	1380	-	2.19E-07	0.00192	CGCCGGAGGCGGCGG
655.	AT3G23220	ERF095	ERF	SbJAZ14	2224	2238	+	2.33E-07	0.00192	AGCCGGCGGCGCGGA
656.	AT3G23220	ERF095	ERF	SbJAZ13	2298	2312	-	2.39E-07	0.00192	CCGCGGCGGCGCCCT
657.	AT3G23220	ERF095	ERF	SbJAZ17	340	354	+	3.76E-07	0.00268	AGGGGGCGGCGGCAG
658.	AT3G23220	ERF095	ERF	SbJAZ18	2186	2200	-	4.71E-07	0.00301	CAGTGGCGGTGCCGA
659.	AT3G23220	ERF095	ERF	SbJAZ17	2031	2045	-	7.05E-07	0.00369	GGGCGGCGGCGAGAA
660.	AT3G23220	ERF095	ERF	SbJAZ13	1853	1867	-	7.49E-07	0.00369	TGGCGGTGGCGACGT
661.	AT3G23220	ERF095	ERF	SbJAZ6	1929	1943	-	8.15E-07	0.00369	GGCCGGCGGTGGAGT
662.	AT3G23220	ERF095	ERF	SbJAZ17	2034	2048	-	8.57E-07	0.00369	GAGGGGCGGCGGCGA
663.	AT3G23220	ERF095	ERF	SbJAZ17	343	357	+	8.64E-07	0.00369	GGGCGGCGGCAGAGC
664.	AT3G23240	ERF092	ERF	SbJAZ12	1360	1387	+	6.41E-09	3.86E-04	ACCCGCCCGCCGCCTCCGGCGCACGCTG
665.	AT3G23240	ERF092	ERF	SbJAZ12	1388	1415	+	1.80E-08	4.77E-04	GACGCTCCGCCGCCGCACACCCTTCCCA

666.	AT3G23240	ERF092	ERF	SbJAZ17	330	357	-	2.38E-08	4.77E-04	GCTCTGCCGCCGCCCCCTCCCGTCCTCC
667.	AT3G23240	ERF092	ERF	SbJAZ14	2214	2241	-	5.62E-08	0.000845	ATCTCCGCGCCGCCGGCTCCAACTCCTC
668.	AT3G23240	ERF092	ERF	SbJAZ7	1944	1971	+	1.62E-07	0.00195	CTCTCCCCGCCGCCACGAGAAGAACGTG
669.	AT3G23240	ERF092	ERF	SbJAZ17	2028	2055	+	1.97E-07	0.00197	CCTTTCTCGCCGCCGCCCCTCTGTTTGC
670.	AT3G23240	ERF092	ERF	SbJAZ5	1717	1744	+	3.08E-07	0.00251	ACGTGGCAGCCGCCGAACCACCCCCGCC
671.	AT3G23240	ERF092	ERF	SbJAZ17	2031	2058	+	3.58E-07	0.00251	TTCTCGCCGCCGCCCCTCTGTTTGCCCC
672.	AT3G23240	ERF092	ERF	SbJAZ12	1562	1589	+	4.10E-07	0.00251	AGAGCGACGCCGCCGATCCGGTCACCGC
673.	AT3G23240	ERF092	ERF	SbJAZ18	1723	1750	-	4.17E-07	0.00251	CTGTTCGCGCCGCCGTGGCGTGGGGACC
674.	AT3G23240	ERF092	ERF	SbJAZ2	1273	1300	+	5.01E-07	0.00274	TGCCAAGCGCCGCCGCCCGTCTGTTCCG
675.	AT3G23240	ERF092	ERF	SbJAZ13	1847	1874	+	8.33E-07	0.00418	CCGATCACGTCGCCACCGCCACCCCAGC
676.	AT3G23240	ERF092	ERF	SbJAZ8	2028	2055	+	9.63E-07	0.00446	CCTCCTCCTCCGCCCCCATTTCCCGACC
677.	AT3G50260	ERF011	ERF	SbJAZ6	1926	1940	+	2.06E-08	1.46E-03	CCGACTCCACCGCCG
678.	AT3G50260	ERF011	ERF	SbJAZ13	1853	1867	+	5.17E-08	0.00183	ACGTCGCCACCGCCA
679.	AT3G50260	ERF011	ERF	SbJAZ11	1817	1831	+	1.60E-07	0.00363	CCACCCGCACCGCCC
680.	AT3G50260	ERF011	ERF	SbJAZ18	1557	1571	+	2.05E-07	0.00363	CGGGCTCCACCGCCC
681.	AT3G50260	ERF011	ERF	SbJAZ6	438	452	-	4.17E-07	0.00574	GCATCGGCACCGCCA
682.	AT3G50260	ERF011	ERF	SbJAZ2	1276	1290	+	4.87E-07	0.00574	CAAGCGCCGCCGCCC
683.	AT3G50260	ERF011	ERF	SbJAZ12	1407	1421	+	6.39E-07	0.00645	CCCTTCCCACCGACA
684.	AT3G50260	ERF011	ERF	SbJAZ17	343	357	-	7.42E-07	0.00655	GCTCTGCCGCCGCCC
685.	AT3G50260	ERF011	ERF	SbJAZ9	1849	1863	+	9.36E-07	0.00735	CCCATCCCACCGCCA
686.	AT3G57600	C2H2	ERF	SbJAZ13	1147	1157	-	5.52E-08	0.00144	CCGCCACCGCT
687.	AT3G57600	ERF051	ERF	SbJAZ13	1856	1866	+	6.68E-08	0.00144	TCGCCACCGCC
688.	AT3G57600	ERF051	ERF	SbJAZ17	2034	2044	+	8.44E-08	0.00144	TCGCCGCCGCC
689.	AT3G57600	ERF051	ERF	SbJAZ2	1279	1289	+	9.89E-08	0.00144	GCGCCGCCGCC
690.	AT3G57600	ERF051	ERF	SbJAZ17	344	354	-	1.05E-07	0.00144	CTGCCGCCGCC
691.	AT3G57600	ERF051	ERF	SbJAZ18	1560	1570	+	2.55E-07	0.00292	GCTCCACCGCC
692.	AT3G57600	ERF051	ERF	SbJAZ12	1391	1401	+	3.59E-07	0.00352	GCTCCGCCGCC
693.	AT3G57600	ERF051	ERF	SbJAZ12	1394	1404	+	4.63E-07	0.00397	CCGCCGCCGCA
694.	AT3G57600	ERF051	ERF	SbJAZ6	1929	1939	+	5.61E-07	0.00428	ACTCCACCGCC
695.	AT4G06746	ERF051	ERF	SbJAZ12	1405	1423	+	1.57E-07	0.0104	CACCCTTCCCACCGACATG
696.	AT4G16750	ERF039	ERF	SbJAZ13	1854	1868	+	3.19E-09	2.16E-04	CGTCGCCACCGCCAC
697.	AT4G16750	ERF039	ERF	SbJAZ6	1927	1941	+	2.66E-07	0.00902	CGACTCCACCGCCGG
698.	AT4G16750	ERF039	ERF	SbJAZ12	1389	1403	+	4.70E-07	0.0105	ACGCTCCGCCGCCGC
699.	AT4G16750	ERF039	ERF	SbJAZ7	1945	1959	+	6.21E-07	0.0105	TCTCCCCGCCGCCAC
700.	AT4G17490	GATA	ERF	SbJAZ17	337	357	-	2.10E-08	6.44E-04	GCTCTGCCGCCGCCCCCTCCC
701.	AT4G17490	ERF103	ERF	SbJAZ12	1360	1380	+	2.60E-08	6.44E-04	ACCCGCCCGCCGCCTCCGGCG
702.	AT4G17490	ERF103	ERF	SbJAZ12	1388	1408	+	3.38E-08	6.44E-04	GACGCTCCGCCGCCGCACACC
703.	AT4G17490	ERF103	ERF	SbJAZ7	1944	1964	+	4.76E-08	0.000644	CTCTCCCCGCCGCCACGAGAA
704.	AT4G17490	ERF103	ERF	SbJAZ17	2028	2048	+	6.56E-08	0.000644	CCTTTCTCGCCGCCGCCCCTC
705.	AT4G17490	ERF103	ERF	SbJAZ14	2221	2241	-	7.15E-08	0.000644	ATCTCCGCGCCGCCGGCTCCA
706.	AT4G17490	ERF103	ERF	SbJAZ13	1853	1873	+	7.64E-08	0.000644	ACGTCGCCACCGCCACCCCAG
707.	AT4G17490	ERF103	ERF	SbJAZ18	1730	1750	-	2.27E-07	0.00167	CTGTTCGCGCCGCCGTGGCGT
708.	AT4G17490	ERF103	ERF	SbJAZ2	1273	1293	+	2.94E-07	0.00193	TGCCAAGCGCCGCCGCCCGTC
709.	AT4G17490	ERF103	ERF	SbJAZ6	1926	1946	+	4.29E-07	0.00253	CCGACTCCACCGCCGGCCGGG
710.	AT4G17490	ERF103	ERF	SbJAZ13	1850	1870	+	7.09E-07	0.00381	ATCACGTCGCCACCGCCACCC
711.	AT4G17490	ERF103	ERF	SbJAZ1	225	245	-	9.29E-07	0.00457	CCTCCCCCGCCACCTCTTTTC
712.	AT4G18450	ERF091	ERF	SbJAZ12	1387	1403	-	1.27E-08	5.85E-04	GCGGCGGCGGAGCGTCC
713.	AT4G18450	ERF091	ERF	SbJAZ7	1943	1959	-	1.76E-08	5.85E-04	GTGGCGGCGGGGAGAGC
714.	AT4G18450	ERF091	ERF	SbJAZ6	1925	1941	-	1.09E-07	0.00193	CCGGCGGTGGAGTCGGG
715.	AT4G18450	ERF091	ERF	SbJAZ13	1852	1868	-	1.30E-07	0.00193	GTGGCGGTGGCGACGTG
716.	AT4G18450	ERF091	ERF	SbJAZ17	2027	2043	-	1.45E-07	0.00193	GCGGCGGCGAGAAAGGG
717.	AT4G18450	ERF091	ERF	SbJAZ18	1735	1751	+	1.81E-07	0.00201	ACGGCGGCGCGAACAGC
718.	AT4G18450	ERF091	ERF	SbJAZ12	1359	1375	-	2.87E-07	0.00259	GAGGCGGCGGGCGGGTG
719.	AT4G18450	ERF091	ERF	SbJAZ1	230	246	+	3.11E-07	0.00259	GAGGTGGCGGGGGAGGG
720.	AT4G18450	ERF091	ERF	SbJAZ2	1272	1288	-	3.73E-07	0.00276	GCGGCGGCGCTTGGCAA
721.	AT4G18450	ERF091	ERF	SbJAZ13	2294	2310	-	6.68E-07	0.00445	GCGGCGGCGCCCTTCTC
722.	AT4G23750	ERF064	ERF	SbJAZ17	344	351	-	6.26E-07	0.0052	CCGCCGCC
723.	AT4G23750	ERF064	ERF	SbJAZ2	1282	1289	+	6.26E-07	0.0052	CCGCCGCC
724.	AT4G23750	ERF064	ERF	SbJAZ12	1366	1373	+	6.26E-07	0.0052	CCGCCGCC
725.	AT4G23750	ERF064	ERF	SbJAZ12	1394	1401	+	6.26E-07	0.0052	CCGCCGCC
726.	AT4G23750	ERF064	ERF	SbJAZ12	1599	1606	+	6.26E-07	0.0052	CCGCCGCC
727.	AT4G23750	ERF064	ERF	SbJAZ7	1950	1957	+	6.26E-07	0.0052	CCGCCGCC
728.	AT4G23750	ERF064	ERF	SbJAZ17	2037	2044	+	6.26E-07	0.0052	CCGCCGCC
729.	AT4G27950	ERF066	ERF	SbJAZ17	337	357	-	1.57E-09	1.09E-04	GCTCTGCCGCCGCCCCCTCCC
730.	AT4G27950	ERF066	ERF	SbJAZ13	1856	1876	+	6.13E-09	1.52E-04	TCGCCACCGCCACCCCAGCGC
731.	AT4G27950	ERF066	ERF	SbJAZ10	638	658	-	9.12E-09	1.52E-04	GCGCTGCCGCCTCGATCGCCG
732.	AT4G27950	ERF066	ERF	SbJAZ17	334	354	-	1.03E-08	1.52E-04	CTGCCGCCGCCCCCTCCCGTC
733.	AT4G27950	ERF066	ERF	SbJAZ8	2031	2051	+	1.10E-08	1.52E-04	CCTCCTCCGCCCCCATTTCCC
734.	AT4G27950	ERF066	ERF	SbJAZ12	1391	1411	+	2.53E-08	2.92E-04	GCTCCGCCGCCGCACACCCTT
735.	AT4G27950	ERF066	ERF	SbJAZ13	1672	1692	-	2.95E-08	2.92E-04	TCCCCGCCTCCCCGGCGGCCG
736.	AT4G27950	ERF066	ERF	SbJAZ12	1366	1386	+	3.82E-08	3.31E-04	CCGCCGCCTCCGGCGCACGCT
737.	AT4G27950	ERF066	ERF	SbJAZ6	429	449	-	4.53E-08	0.000349	TCGGCACCGCCAAATCCGCCG
738.	AT4G27950	ERF066	ERF	SbJAZ2	2215	2235	-	5.56E-08	0.000385	TCGCTACCGCCGCGGGCGCGG
739.	AT4G27950	ERF066	ERF	SbJAZ12	1394	1414	+	1.45E-07	0.000914	CCGCCGCCGCACACCCTTCCC
740.	AT4G27950	ERF066	ERF	SbJAZ5	1734	1754	+	1.71E-07	0.000943	CCACCCCCGCCTCGCGCGCCA

741.	AT4G27950	ERF066	ERF	SbJAZ1	225	245	-	1.77E-07	0.000943	CCTCCCCCGCCACCTCTTTTC
742.	AT4G27950	ERF066	ERF	SbJAZ8	2025	2045	+	1.93E-07	0.000954	GCCCCTCCTCCTCCGCCCCCA
743.	AT4G27950	ERF066	ERF	SbJAZ14	2218	2238	-	2.12E-07	0.000978	TCCGCGCCGCCGGCTCCAACT
744.	AT4G27950	ERF066	ERF	SbJAZ14	1857	1877	+	2.51E-07	0.00109	CCACCTCCTCCCCTCCCGCCC
745.	AT4G27950	ERF066	ERF	SbJAZ8	2022	2042	+	2.77E-07	0.00113	CCTGCCCCTCCTCCTCCGCCC
746.	AT4G27950	ERF066	ERF	SbJAZ13	1853	1873	+	3.35E-07	0.00128	ACGTCGCCACCGCCACCCCAG
747.	AT4G27950	ERF066	ERF	SbJAZ14	2146	2166	+	3.52E-07	0.00128	CAACCACCGCAGCAGCTGCGA
748.	AT4G27950	ERF066	ERF	SbJAZ17	377	397	-	4.05E-07	0.0014	CCACCCCCTCCCCCTCCCCCC
749.	AT4G27950	ERF066	ERF	SbJAZ13	2157	2177	-	4.35E-07	0.00144	CCTCCTCCTCCTCTGCTGCTT
750.	AT4G27950	ERF066	ERF	SbJAZ13	2160	2180	-	5.03E-07	0.00152	CTTCCTCCTCCTCCTCTGCTG
751.	AT4G27950	ERF066	ERF	SbJAZ17	75	95	+	5.32E-07	0.00152	CCGTCCCCGTCCCCGTCCCCG
752.	AT4G27950	ERF066	ERF	SbJAZ2	1913	1933	+	5.49E-07	0.00152	CCTCTGCCGCCCCACCCCTCC
753.	AT4G27950	ERF066	ERF	SbJAZ17	2034	2054	+	5.49E-07	0.00152	TCGCCGCCGCCCCTCTGTTTG
754.	AT4G27950	ERF066	ERF	SbJAZ8	2028	2048	+	6.33E-07	0.00169	CCTCCTCCTCCGCCCCCATTT
755.	AT4G27950	ERF066	ERF	SbJAZ12	1388	1408	+	7.87E-07	0.00198	GACGCTCCGCCGCCGCACACC
756.	AT4G27950	ERF066	ERF	SbJAZ17	81	101	+	8.00E-07	0.00198	CCGTCCCCGTCCCCGCCATCG
757.	AT4G27950	ERF066	ERF	SbJAZ13	1684	1704	-	8.64E-07	0.00206	CCGGCGCCGCGGTCCCCGCCT
758.	AT4G27950	ERF066	ERF	SbJAZ13	1859	1879	+	9.76E-07	0.0022	CCACCGCCACCCCAGCGCGGC
759.	AT4G27950	ERF066	ERF	SbJAZ6	1926	1946	+	9.84E-07	0.0022	CCGACTCCACCGCCGGCCGGG
760.	AT4G28140	ERF054	ERF	SbJAZ18	1551	1570	-	6.02E-09	2.29E-04	GGCGGTGGAGCCCGCGTGGC
761.	AT4G28140	ERF054	ERF	SbJAZ13	1847	1866	-	7.80E-09	2.29E-04	GGCGGTGGCGACGTGATCGG
762.	AT4G28140	ERF054	ERF	SbJAZ6	1920	1939	-	1.25E-08	2.34E-04	GGCGGTGGAGTCGGGGCGTC
763.	AT4G28140	ERF054	ERF	SbJAZ17	2025	2044	-	1.59E-08	2.34E-04	GGCGGCGGCGAGAAAGGGCG
764.	AT4G28140	ERF054	ERF	SbJAZ11	1811	1830	-	2.82E-08	2.84E-04	GGCGGTGCGGGTGGCGTCCC
765.	AT4G28140	ERF054	ERF	SbJAZ6	1923	1942	-	2.91E-08	2.84E-04	GCCGGCGGTGGAGTCGGGGC
766.	AT4G28140	ERF054	ERF	SbJAZ2	1270	1289	-	5.48E-08	0.000459	GGCGGCGGCGCTTGGCAAGG
767.	AT4G28140	ERF054	ERF	SbJAZ12	1382	1401	-	1.07E-07	0.000786	GGCGGCGGAGCGTCCAGCGT
768.	AT4G28140	ERF054	ERF	SbJAZ17	344	363	+	1.47E-07	0.000897	GGCGGCGGCAGAGCTCTCAG
769.	AT4G28140	ERF054	ERF	SbJAZ8	1933	1952	-	1.53E-07	0.000897	GCCGGCGGGTGGGGGGAGGG
770.	AT4G28140	ERF054	ERF	SbJAZ12	1556	1575	-	2.02E-07	0.00108	GGCGGCGTCGCTCTCGGCGG
771.	AT4G28140	ERF054	ERF	SbJAZ10	636	655	+	2.48E-07	0.00121	GACGGCGATCGAGGCGGCAG
772.	AT4G28140	ERF054	ERF	SbJAZ9	1843	1862	-	3.32E-07	0.00143	GGCGGTGGGATGGGAGCGCG
773.	AT4G28140	ERF054	ERF	SbJAZ12	1401	1420	-	3.41E-07	0.00143	GTCGGTGGGAAGGGTGTGCG
774.	AT4G28140	ERF054	ERF	SbJAZ6	439	458	+	3.86E-07	0.0015	GGCGGTGCCGATGCCATACG
775.	AT4G28140	ERF054	ERF	SbJAZ18	1905	1924	-	4.10E-07	0.0015	GGCGGTGGGCCCCGCCACGG
776.	AT4G28140	ERF054	ERF	SbJAZ7	1938	1957	-	6.09E-07	0.0021	GGCGGCGGGGAGAGCTACTG
777.	AT4G28140	ERF054	ERF	SbJAZ1	2040	2059	-	7.17E-07	0.00234	GCCGGTGGTGCTGGTGCGTC
778.	AT4G28140	ERF054	ERF	SbJAZ11	1863	1882	-	8.23E-07	0.00254	GACGGCGCGCGCGCGGGAGA
779.	AT4G28140	ERF054	ERF	SbJAZ13	1104	1123	+	9.74E-07	0.00286	GCCGGCGCCCGGCGAGGCGA
780.	AT4G31060	ERF015	ERF	SbJAZ6	427	446	-	5.32E-08	0.0018	GCACCGCCAAATCCGCCGAT
781.	AT4G31060	ERF015	ERF	SbJAZ6	1853	1872	+	5.50E-08	0.0018	ACACCGACGCCACCACGCCC
782.	AT4G31060	ERF015	ERF	SbJAZ13	1859	1878	+	9.10E-08	0.00198	CCACCGCCACCCCAGCGCGG
783.	AT4G31060	ERF015	ERF	SbJAZ12	1394	1413	+	4.34E-07	0.00511	CCGCCGCCGCACACCCTTCC
784.	AT4G31060	ERF015	ERF	SbJAZ9	1855	1874	+	4.51E-07	0.00511	CCACCGCCAGTTCGCCAACC
785.	AT4G31060	ERF015	ERF	SbJAZ12	1366	1385	+	4.69E-07	0.00511	CCGCCGCCTCCGGCGCACGC
786.	AT4G31060	ERF015	ERF	SbJAZ13	1174	1193	+	5.83E-07	0.00543	CCGCCGACAGATGAGATGCC
787.	AT4G31060	ERF015	ERF	SbJAZ14	2216	2235	-	7.21E-07	0.00543	GCGCCGCCGGCTCCAACTCC
788.	AT4G31060	ERF015	ERF	SbJAZ6	1932	1951	+	7.47E-07	0.00543	CCACCGCCGGCCGGGGCCAC
789.	AT4G34410	ERF109	ERF	SbJAZ7	1948	1962	-	7.33E-07	0.0482	CTCGTGGCGGCGGGG
790.	AT4G36900	ERF009	ERF	SbJAZ13	2300	2309	+	1.05E-07	0.00322	GGCGCCGCCG
791.	AT4G36900	ERF009	ERF	SbJAZ18	1736	1745	-	2.09E-07	0.00322	CGCGCCGCCG
792.	AT4G36900	ERF009	ERF	SbJAZ14	2227	2236	-	2.09E-07	0.00322	CGCGCCGCCG
793.	AT4G36900	ERF009	ERF	SbJAZ2	1278	1287	+	2.79E-07	0.00322	AGCGCCGCCG
794.	AT4G36900	ERF009	ERF	SbJAZ17	343	352	-	4.35E-07	0.00322	GCCGCCGCCC
795.	AT4G36900	ERF009	ERF	SbJAZ2	1281	1290	+	4.35E-07	0.00322	GCCGCCGCCC
796.	AT4G36900	ERF009	ERF	SbJAZ17	2036	2045	+	4.35E-07	0.00322	GCCGCCGCCC
797.	AT4G36900	ERF009	ERF	SbJAZ12	1365	1374	+	4.70E-07	0.00322	CCCGCCGCCT
798.	AT4G36900	ERF009	ERF	SbJAZ12	1598	1607	+	6.10E-07	0.00371	CCCGCCGCCC
799.	AT4G36900	ERF009	ERF	SbJAZ13	2299	2308	-	8.53E-07	0.00467	GGCGGCGCCC
800.	AT5G07310	ERF115	ERF	SbJAZ12	1366	1380	+	4.10E-09	2.91E-04	CCGCCGCCTCCGGCG
801.	AT5G07310	ERF115	ERF	SbJAZ17	337	351	-	6.66E-08	0.00237	CCGCCGCCCCCTCCC
802.	AT5G07310	ERF115	ERF	SbJAZ14	2221	2235	-	1.80E-07	0.00427	GCGCCGCCGGCTCCA
803.	AT5G07310	ERF115	ERF	SbJAZ14	2149	2163	+	2.77E-07	0.00493	CCACCGCAGCAGCTG
804.	AT5G07310	ERF115	ERF	SbJAZ13	1859	1873	+	5.77E-07	0.00626	CCACCGCCACCCCAG
805.	AT5G07310	ERF115	ERF	SbJAZ6	1926	1940	+	6.14E-07	0.00626	CCGACTCCACCGCCG
806.	AT5G07310	ERF115	ERF	SbJAZ13	1125	1139	+	7.27E-07	0.00626	CAGCCATCGCCGTCG
807.	AT5G07310	ERF115	ERF	SbJAZ13	1856	1870	+	7.72E-07	0.00626	TCGCCACCGCCACCC
808.	AT5G07310	ERF115	ERF	SbJAZ17	340	354	-	8.37E-07	0.00626	CTGCCGCCGCCCCCT
809.	AT5G07310	ERF115	ERF	SbJAZ2	2215	2229	-	9.05E-07	0.00626	CCGCCGCGGGCGCGG
810.	AT5G07310	ERF115	ERF	SbJAZ12	1391	1405	+	9.70E-07	0.00626	GCTCCGCCGCCGCAC
811.	AT5G13910	ERF085	ERF	SbJAZ13	1685	1705	-	9.69E-10	6.35E-05	CCCGGCGCCGCGGTCCCCGCC
812.	AT5G13910	ERF085	ERF	SbJAZ12	1390	1410	+	8.76E-09	1.44E-04	CGCTCCGCCGCCGCACACCCT
813.	AT5G13910	ERF085	ERF	SbJAZ13	1855	1875	+	1.02E-08	1.44E-04	GTCGCCACCGCCACCCCAGCG
814.	AT5G13910	ERF085	ERF	SbJAZ14	2216	2236	-	1.04E-08	1.44E-04	CGCGCCGCCGGCTCCAACTCC
815.	AT5G13910	ERF085	ERF	SbJAZ12	1393	1413	+	1.10E-08	1.44E-04	TCCGCCGCCGCACACCCTTCC

816.	AT5G13910	ERF085	ERF	SbJAZ17	335	355	-	2.74E-08	2.99E-04	TCTGCCGCCGCCCCCTCCCGT
817.	AT5G13910	ERF085	ERF	SbJAZ12	1365	1385	+	4.28E-08	3.59E-04	CCCGCCGCCTCCGGCGCACGC
818.	AT5G13910	ERF085	ERF	SbJAZ12	1362	1382	+	4.38E-08	0.000359	CCGCCCGCCGCCTCCGGCGCA
819.	AT5G13910	ERF085	ERF	SbJAZ6	430	450	-	6.12E-08	0.000446	ATCGGCACCGCCAAATCCGCC
820.	AT5G13910	ERF085	ERF	SbJAZ2	2213	2233	-	1.59E-07	0.000996	GCTACCGCCGCGGGCGCGGTC
821.	AT5G13910	ERF085	ERF	SbJAZ14	2219	2239	-	1.67E-07	0.000996	CTCCGCGCCGCCGGCTCCAAC
822.	AT5G13910	ERF085	ERF	SbJAZ2	1278	1298	+	3.51E-07	0.00172	AGCGCCGCCGCCCGTCTGTTC
823.	AT5G13910	ERF085	ERF	SbJAZ13	1673	1693	-	3.71E-07	0.00172	GTCCCCGCCTCCCCGGCGGCC
824.	AT5G13910	ERF085	ERF	SbJAZ8	2030	2050	+	3.82E-07	0.00172	TCCTCCTCCGCCCCCATTTCC
825.	AT5G13910	ERF085	ERF	SbJAZ17	2033	2053	+	3.93E-07	0.00172	CTCGCCGCCGCCCCTCTGTTT
826.	AT5G13910	ERF085	ERF	SbJAZ12	1564	1584	+	5.56E-07	0.00205	AGCGACGCCGCCGATCCGGTC
827.	AT5G13910	ERF085	ERF	SbJAZ18	1897	1917	-	5.61E-07	0.00205	GGCCCCGCCACGGCGACCGGC
828.	AT5G13910	ERF085	ERF	SbJAZ13	1852	1872	+	6.27E-07	0.00205	CACGTCGCCACCGCCACCCCA
829.	AT5G13910	ERF085	ERF	SbJAZ12	1552	1572	+	6.50E-07	0.00205	GGCCCCGCCGAGAGCGACGCC
830.	AT5G13910	ERF085	ERF	SbJAZ10	636	656	-	6.50E-07	0.00205	GCTGCCGCCTCGATCGCCGTC
831.	AT5G13910	ERF085	ERF	SbJAZ6	1931	1951	+	6.56E-07	0.00205	TCCACCGCCGGCCGGGGCCAC
832.	AT5G13910	ERF085	ERF	SbJAZ13	1858	1878	+	8.15E-07	0.00243	GCCACCGCCACCCCAGCGCGG
833.	AT5G13910	ERF085	ERF	SbJAZ6	1928	1948	+	9.91E-07	0.00283	GACTCCACCGCCGGCCGGGGC
834.	AT5G18450	ERF050	ERF	SbJAZ12	1365	1379	-	1.12E-08	5.73E-04	GCCGGAGGCGGCGGG
835.	AT5G18450	ERF050	ERF	SbJAZ17	338	352	+	1.97E-08	5.73E-04	GGAGGGGGCGGCGGC
836.	AT5G18450	ERF050	ERF	SbJAZ13	1858	1872	-	2.37E-08	5.73E-04	TGGGGTGGCGGTGGC
837.	AT5G18450	ERF050	ERF	SbJAZ13	2300	2314	-	7.17E-08	0.0013	GGCCGCGGCGGCGCC
838.	AT5G18450	ERF050	ERF	SbJAZ12	1393	1407	-	1.73E-07	0.00251	GTGTGCGGCGGCGGA
839.	AT5G18450	ERF050	ERF	SbJAZ13	1855	1869	-	5.48E-07	0.00663	GGTGGCGGTGGCGAC
840.	AT5G18450	ERF050	ERF	SbJAZ14	2222	2236	+	9.77E-07	0.0101	GGAGCCGGCGGCGCG
841.	AT5G18560	ERF050	ERF	SbJAZ12	1391	1405	+	4.37E-08	0.00309	GCTCCGCCGCCGCAC
842.	AT5G18560	ERF050	ERF	SbJAZ2	1276	1290	+	8.63E-08	0.00309	CAAGCGCCGCCGCCC
843.	AT5G18560	ERF050	ERF	SbJAZ13	1693	1707	-	2.20E-07	0.0043	CTCCCGGCGCCGCGG
844.	AT5G18560	ERF050	ERF	SbJAZ17	343	357	-	2.40E-07	0.0043	GCTCTGCCGCCGCCC
845.	AT5G18560	ERF050	ERF	SbJAZ17	2031	2045	+	3.39E-07	0.00486	TTCTCGCCGCCGCCC
846.	AT5G18560	ERF050	ERF	SbJAZ13	2298	2312	+	7.39E-07	0.00771	AGGGCGCCGCCGCGG
847.	AT5G18560	ERF050	ERF	SbJAZ12	1388	1402	+	7.53E-07	0.00771	GACGCTCCGCCGCCG
848.	AT5G18560	ERF050	ERF	SbJAZ6	1926	1940	+	8.93E-07	0.00776	CCGACTCCACCGCCG
849.	AT5G18560	ERF050	ERF	SbJAZ13	1853	1867	+	9.75E-07	0.00776	ACGTCGCCACCGCCA
850.	AT5G19790	ERF002	ERF	SbJAZ12	1366	1380	+	1.30E-08	4.98E-04	CCGCCGCCTCCGGCG
851.	AT5G19790	ERF002	ERF	SbJAZ6	1929	1943	+	1.63E-08	4.98E-04	ACTCCACCGCCGGCC
852.	AT5G19790	ERF002	ERF	SbJAZ8	1939	1953	+	5.33E-08	0.00109	CCCCACCCGCCGGCG
853.	AT5G19790	ERF002	ERF	SbJAZ17	343	357	-	9.18E-08	0.00118	GCTCTGCCGCCGCCC
854.	AT5G19790	ERF002	ERF	SbJAZ13	1125	1139	+	9.63E-08	0.00118	CAGCCATCGCCGTCG
855.	AT5G19790	ERF002	ERF	SbJAZ14	2224	2238	-	1.36E-07	0.00123	TCCGCGCCGCCGGCT
856.	AT5G19790	ERF002	ERF	SbJAZ11	1083	1097	-	1.40E-07	0.00123	TCTTCGTCGCCGGCT
857.	AT5G19790	ERF002	ERF	SbJAZ17	2031	2045	+	2.60E-07	0.00199	TTCTCGCCGCCGCCC
858.	AT5G19790	ERF002	ERF	SbJAZ2	1276	1290	+	3.12E-07	0.00211	CAAGCGCCGCCGCCC
859.	AT5G19790	ERF002	ERF	SbJAZ9	2481	2495	-	3.52E-07	0.00211	CCTCGTTCGCCGGCT
860.	AT5G19790	ERF002	ERF	SbJAZ6	1936	1950	-	3.80E-07	0.00211	TGGCCCCGGCCGGCG
861.	AT5G19790	ERF002	ERF	SbJAZ12	1289	1303	+	7.33E-07	0.00374	CACCCGTGGCCGCCC
862.	AT5G19790	ERF002	ERF	SbJAZ7	1944	1958	+	8.07E-07	0.0038	CTCTCCCCGCCGCCA
863.	AT5G19790	ERF002	ERF	SbJAZ11	2034	2048	-	9.12E-07	0.00399	GAACCCCGGCCGGCG
864.	AT5G25190	ERF003	ERF	SbJAZ17	337	355	+	2.69E-09	1.60E-04	GGGAGGGGGCGGCGGCAGA
865.	AT5G25190	ERF003	ERF	SbJAZ13	1855	1873	-	4.77E-09	1.60E-04	CTGGGGTGGCGGTGGCGAC
866.	AT5G25190	ERF003	ERF	SbJAZ12	1390	1408	-	5.07E-08	0.00113	GGTGTGCGGCGGCGGAGCG
867.	AT5G25190	ERF003	ERF	SbJAZ12	1362	1380	-	8.33E-08	0.00131	CGCCGGAGGCGGCGGGCGG
868.	AT5G25190	ERF003	ERF	SbJAZ12	1393	1411	-	9.78E-08	0.00131	AAGGGTGTGCGGCGGCGGA
869.	AT5G25190	ERF003	ERF	SbJAZ13	1687	1705	+	1.49E-07	0.00167	CGGGGACCGCGGCGCCGGG
870.	AT5G25190	ERF003	ERF	SbJAZ6	432	450	+	2.03E-07	0.00194	CGGATTTGGCGGTGCCGAT
871.	AT5G25190	ERF003	ERF	SbJAZ12	1365	1383	-	2.44E-07	0.00196	GTGCGCCGGAGGCGGCGGG
872.	AT5G25190	ERF003	ERF	SbJAZ14	2218	2236	+	2.62E-07	0.00196	AGTTGGAGCCGGCGGCGCG
873.	AT5G25190	ERF003	ERF	SbJAZ8	2027	2045	-	4.91E-07	0.00271	TGGGGGCGGAGGAGGAGGG
874.	AT5G25190	ERF003	ERF	SbJAZ18	2185	2203	-	4.96E-07	0.00271	CACCAGTGGCGGTGCCGAA
875.	AT5G25190	ERF003	ERF	SbJAZ13	1858	1876	-	5.37E-07	0.00271	GCGCTGGGGTGGCGGTGGC
876.	AT5G25190	ERF003	ERF	SbJAZ17	2033	2051	-	5.81E-07	0.00271	ACAGAGGGGCGGCGGCGAG
877.	AT5G25190	ERF003	ERF	SbJAZ14	2221	2239	+	5.93E-07	0.00271	TGGAGCCGGCGGCGCGGAG
878.	AT5G25190	ERF003	ERF	SbJAZ8	2030	2048	-	6.05E-07	0.00271	AAATGGGGGCGGAGGAGGA
879.	AT5G25190	ERF003	ERF	SbJAZ18	1559	1577	-	7.47E-07	0.00313	TCGGTGGGGCGGTGGAGCC
880.	AT5G25190	ERF003	ERF	SbJAZ6	429	447	+	8.68E-07	0.00343	CGGCGGATTTGGCGGTGCC
881.	AT5G25390	ERF005	ERF	SbJAZ8	2024	2044	+	1.66E-09	1.13E-04	TGCCCCTCCTCCTCCGCCCCC
882.	AT5G25390	ERF005	ERF	SbJAZ14	2210	2230	-	7.91E-09	2.57E-04	GCCGGCTCCAACTCCTCCGCC
883.	AT5G25390	ERF005	ERF	SbJAZ8	2021	2041	+	1.64E-08	2.57E-04	CCCTGCCCCTCCTCCTCCGCC
884.	AT5G25390	ERF005	ERF	SbJAZ12	1362	1382	+	1.97E-08	2.57E-04	CCGCCCGCCGCCTCCGGCGCA
885.	AT5G25390	ERF005	ERF	SbJAZ13	2158	2178	-	2.35E-08	2.57E-04	TCCTCCTCCTCCTCTGCTGCT
886.	AT5G25390	ERF005	ERF	SbJAZ14	2216	2236	-	2.39E-08	2.57E-04	CGCGCCGCCGGCTCCAACTCC
887.	AT5G25390	ERF005	ERF	SbJAZ1	229	249	-	2.64E-08	2.57E-04	CCCCCCTCCCCCGCCACCTCT
888.	AT5G25390	ERF005	ERF	SbJAZ6	427	447	-	9.96E-08	0.000693	GGCACCGCCAAATCCGCCGAT
889.	AT5G25390	ERF005	ERF	SbJAZ10	636	656	-	1.02E-07	0.000693	GCTGCCGCCTCGATCGCCGTC
890.	AT5G25390	ERF005	ERF	SbJAZ14	2213	2233	-	1.11E-07	0.000693	GCCGCCGGCTCCAACTCCTCC

891.	AT5G25390	ERF005	ERF	SbJAZ12	2098	2118	-	1.12E-07	0.000693	AGCACCTCCTCGTCCTCCCCC
892.	AT5G25390	ERF005	ERF	SbJAZ17	378	398	-	1.52E-07	0.000854	ACCACCCCCTCCCCCTCCCCC
893.	AT5G25390	ERF005	ERF	SbJAZ10	639	659	-	1.65E-07	0.000854	CGCGCTGCCGCCTCGATCGCC
894.	AT5G25390	ERF005	ERF	SbJAZ13	2161	2181	-	1.76E-07	0.000854	GCTTCCTCCTCCTCCTCTGCT
895.	AT5G25390	ERF005	ERF	SbJAZ14	2148	2168	+	1.94E-07	0.00088	ACCACCGCAGCAGCTGCGACC
896.	AT5G25390	ERF005	ERF	SbJAZ13	1823	1843	+	2.61E-07	0.00111	CCCCCCTGCCCCTCCTCCGTC
897.	AT5G25390	ERF005	ERF	SbJAZ14	2145	2165	+	3.31E-07	0.00129	GCAACCACCGCAGCAGCTGCG
898.	AT5G25390	ERF005	ERF	SbJAZ6	1922	1942	+	3.41E-07	0.00129	CGCCCCGACTCCACCGCCGGC
899.	AT5G25390	ERF005	ERF	SbJAZ13	2164	2184	-	4.29E-07	0.0015	CTCGCTTCCTCCTCCTCCTCT
900.	AT5G25390	ERF005	ERF	SbJAZ8	2030	2050	+	4.49E-07	0.0015	TCCTCCTCCGCCCCCATTTCC
901.	AT5G25390	ERF005	ERF	SbJAZ6	1849	1869	+	4.62E-07	0.0015	GTCAACACCGACGCCACCACG
902.	AT5G25390	ERF005	ERF	SbJAZ13	1852	1872	+	5.27E-07	0.00161	CACGTCGCCACCGCCACCCCA
903.	AT5G25390	ERF005	ERF	SbJAZ1	226	246	-	5.58E-07	0.00161	CCCTCCCCCGCCACCTCTTTT
904.	AT5G25390	ERF005	ERF	SbJAZ5	291	311	+	5.66E-07	0.00161	TTCTCCGCCTCATCCGAGACC
905.	AT5G25390	ERF005	ERF	SbJAZ17	338	358	-	7.03E-07	0.00191	AGCTCTGCCGCCGCCCCCTCC
906.	AT5G25390	ERF005	ERF	SbJAZ10	1867	1887	+	7.61E-07	0.00197	CGCACCTCCAGCTCCATCGCG
907.	AT5G25390	ERF005	ERF	SbJAZ6	11	31	-	7.83E-07	0.00197	TCCACAACCGCCTCATCGGCA
908.	AT5G25390	ERF005	ERF	SbJAZ2	2213	2233	-	9.02E-07	0.00219	GCTACCGCCGCGGGCGCGGTC
909.	AT5G25390	ERF005	ERF	SbJAZ6	1905	1925	+	9.35E-07	0.00219	GCCTCCCTCTCCCCCGACGCC
910.	AT5G25390	ERF005	ERF	SbJAZ13	1673	1693	-	9.96E-07	0.00226	GTCCCCGCCTCCCCGGCGGCC
911.	AT5G25810	ERF040	ERF	SbJAZ13	1857	1871	+	4.27E-07	0.0285	CGCCACCGCCACCCC
912.	AT5G43410	ERF096	ERF	SbJAZ12	1394	1403	+	1.76E-08	6.82E-04	CCGCCGCCGC
913.	AT5G43410	ERF096	ERF	SbJAZ2	1279	1288	+	3.52E-08	6.82E-04	GCGCCGCCGC
914.	AT5G43410	ERF096	ERF	SbJAZ13	2301	2310	+	3.52E-08	6.82E-04	GCGCCGCCGC
915.	AT5G43410	ERF096	ERF	SbJAZ14	2226	2235	-	7.04E-08	0.00102	GCGCCGCCGG
916.	AT5G43410	ERF096	ERF	SbJAZ7	1950	1959	+	1.05E-07	0.00115	CCGCCGCCAC
917.	AT5G43410	ERF096	ERF	SbJAZ17	342	351	-	1.58E-07	0.00115	CCGCCGCCCC
918.	AT5G43410	ERF096	ERF	SbJAZ12	1599	1608	+	1.58E-07	0.00115	CCGCCGCCCC
919.	AT5G43410	ERF096	ERF	SbJAZ17	2037	2046	+	1.58E-07	0.00115	CCGCCGCCCC
920.	AT5G43410	ERF096	ERF	SbJAZ2	1282	1291	+	2.63E-07	0.0017	CCGCCGCCCG
921.	AT5G43410	ERF096	ERF	SbJAZ18	1735	1744	-	3.50E-07	0.00203	GCGCCGCCGT
922.	AT5G43410	ERF096	ERF	SbJAZ12	1366	1375	+	5.93E-07	0.00313	CCGCCGCCTC
923.	AT5G43410	ERF096	ERF	SbJAZ17	2034	2043	+	7.32E-07	0.00355	TCGCCGCCGC
924.	AT5G44210	ERF9	ERF	SbJAZ6	429	449	+	9.76E-09	3.60E-04	CGGCGGATTTGGCGGTGCCGA
925.	AT5G44210	ERF9	ERF	SbJAZ13	1684	1704	+	1.11E-08	3.60E-04	AGGCGGGGACCGCGGCGCCGG
926.	AT5G44210	ERF9	ERF	SbJAZ13	1856	1876	-	1.92E-08	3.60E-04	GCGCTGGGGTGGCGGTGGCGA
927.	AT5G44210	ERF9	ERF	SbJAZ17	337	357	+	2.48E-08	3.60E-04	GGGAGGGGGCGGCGGCAGAGC
928.	AT5G44210	ERF9	ERF	SbJAZ12	1366	1386	-	3.05E-08	3.60E-04	AGCGTGCGCCGGAGGCGGCGG
929.	AT5G44210	ERF9	ERF	SbJAZ14	2215	2235	+	3.54E-08	3.60E-04	AGGAGTTGGAGCCGGCGGCGC
930.	AT5G44210	ERF9	ERF	SbJAZ17	334	354	+	3.90E-08	3.60E-04	GACGGGAGGGGGCGGCGGCAG
931.	AT5G44210	ERF9	ERF	SbJAZ12	1394	1414	-	4.29E-08	3.60E-04	GGGAAGGGTGTGCGGCGGCGG
932.	AT5G44210	ERF9	ERF	SbJAZ12	1391	1411	-	4.57E-08	0.00036	AAGGGTGTGCGGCGGCGGAGC
933.	AT5G44210	ERF9	ERF	SbJAZ10	638	658	+	5.82E-08	0.000413	CGGCGATCGAGGCGGCAGCGC
934.	AT5G44210	ERF9	ERF	SbJAZ13	1853	1873	-	9.43E-08	0.000569	CTGGGGTGGCGGTGGCGACGT
935.	AT5G44210	ERF9	ERF	SbJAZ12	1363	1383	-	9.62E-08	0.000569	GTGCGCCGGAGGCGGCGGGCG
936.	AT5G44210	ERF9	ERF	SbJAZ8	2031	2051	-	3.81E-07	0.00208	GGGAAATGGGGGCGGAGGAGG
937.	AT5G44210	ERF9	ERF	SbJAZ13	1672	1692	+	4.18E-07	0.00212	CGGCCGCCGGGGAGGCGGGGA
938.	AT5G44210	ERF9	ERF	SbJAZ10	635	655	+	5.02E-07	0.00237	AGACGGCGATCGAGGCGGCAG
939.	AT5G44210	ERF9	ERF	SbJAZ2	2212	2232	+	7.14E-07	0.00316	GGACCGCGCCCGCGGCGGTAG
940.	AT5G44210	ERF9	ERF	SbJAZ2	1913	1933	-	7.94E-07	0.00331	GGAGGGGTGGGGCGGCAGAGG
941.	AT5G44210	ERF9	ERF	SbJAZ2	2215	2235	+	8.98E-07	0.00333	CCGCGCCCGCGGCGGTAGCGA
942.	AT5G44210	ERF9	ERF	SbJAZ14	1857	1877	-	9.30E-07	0.00333	GGGCGGGAGGGGAGGAGGTGG
943.	AT5G44210	ERF9	ERF	SbJAZ12	1568	1588	-	9.39E-07	0.00333	CGGTGACCGGATCGGCGGCGT
944.	AT5G44210	ERF9	ERF	SbJAZ14	2218	2238	+	9.89E-07	0.00334	AGTTGGAGCCGGCGGCGCGGA
945.	AT5G47220	ERF2	ERF	SbJAZ2	1270	1290	+	3.56E-08	8.46E-04	CCTTGCCAAGCGCCGCCGCCC
946.	AT5G47220	ERF2	ERF	SbJAZ12	1385	1405	+	4.03E-08	8.46E-04	CTGGACGCTCCGCCGCCGCAC
947.	AT5G47220	ERF2	ERF	SbJAZ18	1733	1753	-	4.47E-08	0.000846	TCGCTGTTCGCGCCGCCGTGG
948.	AT5G47220	ERF2	ERF	SbJAZ1	228	248	-	5.11E-08	0.000846	CCCCCTCCCCCGCCACCTCTT
949.	AT5G47220	ERF2	ERF	SbJAZ7	1941	1961	+	5.98E-08	0.000846	TAGCTCTCCCCGCCGCCACGA
950.	AT5G47220	ERF2	ERF	SbJAZ17	2025	2045	+	8.56E-08	0.000933	CGCCCTTTCTCGCCGCCGCCC
951.	AT5G47220	ERF2	ERF	SbJAZ6	1923	1943	+	9.23E-08	0.000933	GCCCCGACTCCACCGCCGGCC
952.	AT5G47220	ERF2	ERF	SbJAZ14	1968	1988	+	1.14E-07	0.00101	CGCCCTCTACCTCCGCCAGCG
953.	AT5G47220	ERF2	ERF	SbJAZ13	1847	1867	+	1.62E-07	0.00128	CCGATCACGTCGCCACCGCCA
954.	AT5G47220	ERF2	ERF	SbJAZ12	1382	1402	+	2.09E-07	0.0014	ACGCTGGACGCTCCGCCGCCG
955.	AT5G47220	ERF2	ERF	SbJAZ12	1360	1380	+	2.22E-07	0.0014	ACCCGCCCGCCGCCTCCGGCG
956.	AT5G47220	ERF2	ERF	SbJAZ12	1357	1377	+	2.57E-07	0.0014	AGCACCCGCCCGCCGCCTCCG
957.	AT5G47220	ERF2	ERF	SbJAZ12	1556	1576	+	2.57E-07	0.0014	CCGCCGAGAGCGACGCCGCCG
958.	AT5G47220	ERF2	ERF	SbJAZ10	632	652	-	4.24E-07	0.00214	CCGCCTCGATCGCCGTCTCGG
959.	AT5G47220	ERF2	ERF	SbJAZ17	343	363	-	5.55E-07	0.00248	CTGAGAGCTCTGCCGCCGCCC
960.	AT5G47220	ERF2	ERF	SbJAZ13	1850	1870	+	5.60E-07	0.00248	ATCACGTCGCCACCGCCACCC
961.	AT5G47220	ERF2	ERF	SbJAZ14	2224	2244	-	6.30E-07	0.00262	CAAATCTCCGCGCCGCCGGCT
962.	AT5G47230	ERF5	ERF	SbJAZ2	1276	1290	+	4.56E-08	0.00199	CAAGCGCCGCCGCCC
963.	AT5G47230	ERF5	ERF	SbJAZ6	1926	1940	+	9.36E-08	0.00199	CCGACTCCACCGCCG
964.	AT5G47230	ERF5	ERF	SbJAZ13	1853	1867	+	1.03E-07	0.00199	ACGTCGCCACCGCCA
965.	AT5G47230	ERF5	ERF	SbJAZ12	1363	1377	+	1.31E-07	0.00199	CGCCCGCCGCCTCCG

966.	AT5G47230	ERF5	ERF	SbJAZ12	1366	1380	+	1.38E-07	0.00199	CCGCCGCCTCCGGCG
967.	AT5G47230	ERF5	ERF	SbJAZ12	1391	1405	+	2.32E-07	0.00279	GCTCCGCCGCCGCAC
968.	AT5G47230	ERF5	ERF	SbJAZ13	1693	1707	-	3.96E-07	0.00312	CTCCCGGCGCCGCGG
969.	AT5G47230	ERF5	ERF	SbJAZ13	1856	1870	+	4.28E-07	0.00312	TCGCCACCGCCACCC
970.	AT5G47230	ERF5	ERF	SbJAZ7	1947	1961	+	4.28E-07	0.00312	TCCCCGCCGCCACGA
971.	AT5G47230	ERF5	ERF	SbJAZ12	1388	1402	+	4.66E-07	0.00312	GACGCTCCGCCGCCG
972.	AT5G47230	ERF5	ERF	SbJAZ14	2221	2235	-	4.76E-07	0.00312	GCGCCGCCGGCTCCA
973.	AT5G47230	ERF5	ERF	SbJAZ17	343	357	-	5.82E-07	0.00339	GCTCTGCCGCCGCCC
974.	AT5G47230	ERF5	ERF	SbJAZ14	2224	2238	-	6.10E-07	0.00339	TCCGCGCCGCCGGCT
975.	AT5G47230	ERF5	ERF	SbJAZ17	2031	2045	+	7.58E-07	0.0039	TTCTCGCCGCCGCCC
976.	AT5G47230	ERF5	ERF	SbJAZ8	2028	2042	+	8.38E-07	0.00403	CCTCCTCCTCCGCCC
977.	AT5G47230	ERF5	ERF	SbJAZ14	1974	1988	+	9.74E-07	0.00439	CTACCTCCGCCAGCG
978.	AT5G51190	ERF105	ERF	SbJAZ12	1392	1406	-	3.75E-08	1.01E-03	TGTGCGGCGGCGGAG
979.	AT5G51190	ERF105	ERF	SbJAZ14	2223	2237	+	4.24E-08	1.01E-03	GAGCCGGCGGCGCGG
980.	AT5G51190	ERF105	ERF	SbJAZ13	2299	2313	-	4.43E-08	0.00101	GCCGCGGCGGCGCCC
981.	AT5G51190	ERF105	ERF	SbJAZ7	1948	1962	-	7.76E-08	0.00132	CTCGTGGCGGCGGGG
982.	AT5G51190	ERF105	ERF	SbJAZ13	1857	1871	-	1.29E-07	0.00176	GGGGTGGCGGTGGCG
983.	AT5G51190	ERF105	ERF	SbJAZ17	2032	2046	-	1.63E-07	0.00186	GGGGCGGCGGCGAGA
984.	AT5G51190	ERF105	ERF	SbJAZ13	1854	1868	-	2.03E-07	0.00189	GTGGCGGTGGCGACG
985.	AT5G51190	ERF105	ERF	SbJAZ18	1732	1746	+	2.21E-07	0.00189	GCCACGGCGGCGCGA
986.	AT5G51190	ERF105	ERF	SbJAZ2	1277	1291	-	3.31E-07	0.0025	CGGGCGGCGGCGCTT
987.	AT5G51190	ERF105	ERF	SbJAZ12	1364	1378	-	4.08E-07	0.00278	CCGGAGGCGGCGGGC
988.	AT5G51190	ERF105	ERF	SbJAZ12	1389	1403	-	5.81E-07	0.0036	GCGGCGGCGGAGCGT
989.	AT5G51190	ERF105	ERF	SbJAZ18	2187	2201	-	9.44E-07	0.00536	CCAGTGGCGGTGCCG
990.	AT5G52020	ERF025	ERF	SbJAZ13	1260	1274	-	1.07E-08	7.36E-04	CGCTGTCGGCAACGG
991.	AT5G61600	ERF104	ERF	SbJAZ6	1924	1942	+	1.10E-08	7.36E-04	CCCCGACTCCACCGCCGGC
992.	AT5G61600	ERF104	ERF	SbJAZ13	1851	1869	+	5.00E-08	0.000749	TCACGTCGCCACCGCCACC
993.	AT5G61600	ERF104	ERF	SbJAZ7	1942	1960	+	5.39E-08	0.000749	AGCTCTCCCCGCCGCCACG
994.	AT5G61600	ERF104	ERF	SbJAZ12	1386	1404	+	5.52E-08	0.000749	TGGACGCTCCGCCGCCGCA
995.	AT5G61600	ERF104	ERF	SbJAZ1	229	247	-	5.58E-08	0.000749	CCCCTCCCCCGCCACCTCT
996.	AT5G61600	ERF104	ERF	SbJAZ17	2026	2044	+	7.02E-08	0.000785	GCCCTTTCTCGCCGCCGCC
997.	AT5G61600	ERF104	ERF	SbJAZ2	1271	1289	+	1.01E-07	0.000883	CTTGCCAAGCGCCGCCGCC
998.	AT5G61600	ERF104	ERF	SbJAZ18	1734	1752	-	1.05E-07	0.000883	CGCTGTTCGCGCCGCCGTG
999.	AT5G61600	ERF104	ERF	SbJAZ12	1361	1379	+	1.32E-07	0.000985	CCCGCCCGCCGCCTCCGGC
1000.	AT5G61600	ERF104	ERF	SbJAZ12	1358	1376	+	1.65E-07	0.00111	GCACCCGCCCGCCGCCTCC
1001.	AT5G61600	ERF104	ERF	SbJAZ12	1383	1401	+	3.95E-07	0.00241	CGCTGGACGCTCCGCCGCC
1002.	AT5G61600	ERF104	ERF	SbJAZ17	85	103	+	4.63E-07	0.00259	CCCCGTCCCCGCCATCGGG
1003.	AT5G61600	ERF104	ERF	SbJAZ10	633	651	-	5.46E-07	0.00282	CGCCTCGATCGCCGTCTCG
1004.	AT5G61600	ERF104	ERF	SbJAZ5	1825	1843	+	6.48E-07	0.00292	CGCACCGGGCGCCGTCAAC
1005.	AT5G61600	ERF104	ERF	SbJAZ14	1969	1987	+	7.08E-07	0.00292	GCCCTCTACCTCCGCCAGC
1006.	AT5G61600	ERF104	ERF	SbJAZ13	1848	1866	+	7.15E-07	0.00292	CGATCACGTCGCCACCGCC
1007.	AT5G61600	ERF104	ERF	SbJAZ6	1876	1894	+	7.40E-07	0.00292	CGCGGCCGTGGCCACCGCG
1008.	AT5G61600	ERF104	ERF	SbJAZ13	1123	1141	+	8.82E-07	0.00312	ATCAGCCATCGCCGTCGAC
1009.	AT5G61600	ERF104	ERF	SbJAZ6	1851	1869	+	8.82E-07	0.00312	CAACACCGACGCCACCACG
1010.	AT5G61600	ERF104	ERF	SbJAZ11	1867	1885	+	9.45E-07	0.00317	CCGCGCGCGCGCCGTCTTC
1011.	AT5G61600	ERF104	ERF	SbJAZ13	2293	2311	+	9.95E-07	0.00318	GGAGAAGGGCGCCGCCGCG
1012.	AT5G64750	ERF111	ERF	SbJAZ13	1685	1703	+	3.97E-09	2.49E-04	GGCGGGGACCGCGGCGCCG
1013.	AT5G64750	ERF111	ERF	SbJAZ6	430	448	+	4.36E-08	7.11E-04	GGCGGATTTGGCGGTGCCG
1014.	AT5G64750	ERF111	ERF	SbJAZ12	1392	1410	-	4.42E-08	0.000711	AGGGTGTGCGGCGGCGGAG
1015.	AT5G64750	ERF111	ERF	SbJAZ12	1395	1413	-	5.73E-08	0.000711	GGAAGGGTGTGCGGCGGCG
1016.	AT5G64750	ERF111	ERF	SbJAZ14	2216	2234	+	5.73E-08	0.000711	GGAGTTGGAGCCGGCGGCG
1017.	AT5G64750	ERF111	ERF	SbJAZ13	1857	1875	-	6.81E-08	0.000711	CGCTGGGGTGGCGGTGGCG
1018.	AT5G64750	ERF111	ERF	SbJAZ17	338	356	+	1.22E-07	0.00107	GGAGGGGGCGGCGGCAGAG
1019.	AT5G64750	ERF111	ERF	SbJAZ12	1364	1382	-	1.45E-07	0.00107	TGCGCCGGAGGCGGCGGGC
1020.	AT5G64750	ERF111	ERF	SbJAZ17	335	353	+	1.53E-07	0.00107	ACGGGAGGGGGCGGCGGCA
1021.	AT5G64750	ERF111	ERF	SbJAZ12	1566	1584	-	2.55E-07	0.0016	GACCGGATCGGCGGCGTCG
1022.	AT5G64750	ERF111	ERF	SbJAZ14	2219	2237	+	3.33E-07	0.0019	GTTGGAGCCGGCGGCGCGG
1023.	AT5G64750	ERF111	ERF	SbJAZ10	639	657	+	6.00E-07	0.00298	GGCGATCGAGGCGGCAGCG
1024.	AT5G64750	ERF111	ERF	SbJAZ17	2035	2053	-	6.18E-07	0.00298	AAACAGAGGGGCGGCGGCG
1025.	AT5G65130	ERF057	ERF	SbJAZ13	1853	1867	+	3.70E-08	1.09E-03	ACGTCGCCACCGCCA
1026.	AT5G65130	ERF057	ERF	SbJAZ2	1276	1290	+	3.88E-08	1.09E-03	CAAGCGCCGCCGCCC
1027.	AT5G65130	ERF057	ERF	SbJAZ6	1926	1940	+	4.83E-08	0.00109	CCGACTCCACCGCCG
1028.	AT5G65130	ERF057	ERF	SbJAZ17	343	357	-	9.50E-08	0.00161	GCTCTGCCGCCGCCC
1029.	AT5G65130	ERF057	ERF	SbJAZ13	1856	1870	+	1.25E-07	0.00169	TCGCCACCGCCACCC
1030.	AT5G65130	ERF057	ERF	SbJAZ12	1391	1405	+	1.86E-07	0.00188	GCTCCGCCGCCGCAC
1031.	AT5G65130	ERF057	ERF	SbJAZ8	2028	2042	+	1.94E-07	0.00188	CCTCCTCCTCCGCCC
1032.	AT5G65130	ERF057	ERF	SbJAZ8	2031	2045	+	2.36E-07	0.00196	CCTCCTCCGCCCCCA
1033.	AT5G65130	ERF057	ERF	SbJAZ17	2031	2045	+	2.60E-07	0.00196	TTCTCGCCGCCGCCC
1034.	AT5G65130	ERF057	ERF	SbJAZ1	234	248	-	3.28E-07	0.00206	CCCCCTCCCCCGCCA
1035.	AT5G65130	ERF057	ERF	SbJAZ18	1557	1571	+	3.34E-07	0.00206	CGGGCTCCACCGCCC
1036.	AT5G65130	ERF057	ERF	SbJAZ12	1363	1377	+	4.98E-07	0.00275	CGCCCGCCGCCTCCG
1037.	AT5G65130	ERF057	ERF	SbJAZ6	438	452	-	5.29E-07	0.00275	GCATCGGCACCGCCA
1038.	AT5G65130	ERF057	ERF	SbJAZ12	1388	1402	+	7.04E-07	0.0034	GACGCTCCGCCGCCG
1039.	AT5G65130	ERF057	ERF	SbJAZ17	340	354	-	9.02E-07	0.00407	CTGCCGCCGCCCCCT
1040.	AT5G67000	ERF122	ERF	SbJAZ12	1394	1408	-	2.80E-08	1.89E-03	GGTGTGCGGCGGCGG

1041.	AT5G67000	ERF122	ERF	SbJAZ18	1730	1744	+	1.10E-07	0.00372	ACGCCACGGCGGCGC
1042.	AT5G67000	ERF122	ERF	SbJAZ10	479	493	-	2.67E-07	0.00451	TCAACGCGGCTGCGG
1043.	AT5G67000	ERF122	ERF	SbJAZ12	1391	1405	-	2.67E-07	0.00451	GTGCGGCGGCGGAGC
1044.	AT5G67000	ERF122	ERF	SbJAZ14	1719	1733	-	5.91E-07	0.00727	CGTGCGCGGTGGTGG
1045.	AT5G67000	ERF122	ERF	SbJAZ14	2146	2160	-	6.45E-07	0.00727	CTGCTGCGGTGGTTG
1046.	AT5G67000	ERF122	ERF	SbJAZ12	1363	1377	-	8.22E-07	0.00794	CGGAGGCGGCGGGCG
1047.	AT5G67190	ERF010	ERF	SbJAZ13	1853	1867	+	2.74E-08	1.09E-03	ACGTCGCCACCGCCA
1048.	AT5G67190	ERF010	ERF	SbJAZ6	1926	1940	+	3.34E-08	1.09E-03	CCGACTCCACCGCCG
1049.	AT5G67190	ERF010	ERF	SbJAZ18	1557	1571	+	4.02E-07	0.00806	CGGGCTCCACCGCCC
1050.	AT5G67190	ERF010	ERF	SbJAZ6	438	452	-	4.92E-07	0.00806	GCATCGGCACCGCCA
1051.	AT5G67190	ERF010	ERF	SbJAZ12	1407	1421	+	7.15E-07	0.00833	CCCTTCCCACCGACA
1052.	AT5G67190	ERF010	ERF	SbJAZ11	1817	1831	+	7.62E-07	0.00833	CCACCCGCACCGCCC
1053.	AT3G22170	FAR1	FAR1	SbJAZ9	1743	1754	+	5.29E-08	0.00351	CCCACGCGCCCG
1054.	AT3G22170	FAR1	FAR1	SbJAZ14	2075	2086	-	1.02E-07	0.00351	CCCACGCGCTTA
1055.	AT3G22170	FAR1	FAR1	SbJAZ9	2092	2103	+	1.61E-07	0.0037	GTCACGCGCTCG
1056.	AT3G22170	FAR1	FAR1	SbJAZ10	1637	1648	-	5.01E-07	0.00863	CCCACGCGCGCG
1057.	AT3G22170	FAR1	FAR1	SbJAZ9	1881	1892	+	6.84E-07	0.00943	CCCACGCGCGCC
1058.	AT4G15090	FAR1	FAR1	SbJAZ9	1741	1755	+	6.06E-09	3.98E-04	CCCCCACGCGCCCGC
1059.	AT4G15090	FAR1	FAR1	SbJAZ14	2074	2088	-	1.21E-07	0.00395	CCCCCACGCGCTTAT
1060.	AT4G15090	FAR1	FAR1	SbJAZ9	1879	1893	+	2.14E-07	0.00431	CCCCCACGCGCGCCT
1061.	AT4G15090	FAR1	FAR1	SbJAZ12	210	224	-	3.29E-07	0.00431	CCCCCACGCGCGGGA
1062.	AT4G15090	FAR1	FAR1	SbJAZ4	655	669	-	3.29E-07	0.00431	CCCCCACGCGCGGGA
1063.	AT4G15090	FAR1	FAR1	SbJAZ18	1430	1444	+	4.14E-07	0.00452	CACACACGCGCCACT
1064.	AT4G15090	FAR1	FAR1	SbJAZ10	1636	1650	-	8.50E-07	0.00796	CACCCACGCGCGCGC
1065.	AT4G15090	FAR1	FAR1	SbJAZ9	2090	2104	+	9.90E-07	0.00811	GCGTCACGCGCTCGC
1066.	AT3G21175	GATA	GATA	SbJAZ11	1166	1180	+	8.25E-07	0.0644	TCATCATCTTGATCA
1067.	AT3G24050	GATA	GATA	SbJAZ13	1854	1868	+	3.11E-07	0.0228	GTGGCGGTGGCGACG
1068.	AT3G24050	GATA	GATA	SbJAZ6	1854	1868	-	5.92E-07	0.0228	GTGGTGGCGTCGGTG
1069.	AT4G36620	GATA	GATA	SbJAZ4	629	639	+	7.46E-07	0.0539	CCGATCGGGTC
1070.	AT1G66420	GeBP	GeBP	SbJAZ18	569	587	+	3.24E-07	0.0252	AAACTCATCCATTACTTGA
1071.	AT4G00270	GeBP	GeBP	SbJAZ10	605	612	-	6.26E-07	0.021	CCGGCCGC
1072.	AT4G00270	GeBP	GeBP	SbJAZ13	1671	1678	+	6.26E-07	0.021	CCGGCCGC
1073.	AT2G01570	GRAS	GRAS	SbJAZ1	2012	2031	+	1.61E-08	7.45E-04	GAGAGAGAGAGAAAGAGAGG
1074.	AT2G01570	GRAS	GRAS	SbJAZ1	2010	2029	+	8.22E-08	0.000745	GAGAGAGAGAGAGAAAGAGA
1075.	AT2G01570	GRAS	GRAS	SbJAZ17	2107	2126	+	8.70E-08	0.000745	GAGAAGGAGAGAGAGAGAGG
1076.	AT2G01570	GRAS	GRAS	SbJAZ18	2072	2091	+	1.04E-07	0.000745	GAGAGAGAGAGAGAAGAGAG
1077.	AT2G01570	GRAS	GRAS	SbJAZ1	1992	2011	+	1.19E-07	0.000745	GAGAGAGAGAGAGAGAGAGA
1078.	AT2G01570	GRAS	GRAS	SbJAZ1	1994	2013	+	1.19E-07	0.000745	GAGAGAGAGAGAGAGAGAGA
1079.	AT2G01570	GRAS	GRAS	SbJAZ1	1996	2015	+	1.19E-07	0.000745	GAGAGAGAGAGAGAGAGAGA
1080.	AT2G01570	GRAS	GRAS	SbJAZ1	1998	2017	+	1.19E-07	0.000745	GAGAGAGAGAGAGAGAGAGA
1081.	AT2G01570	GRAS	GRAS	SbJAZ1	2000	2019	+	1.19E-07	0.000745	GAGAGAGAGAGAGAGAGAGA
1082.	AT2G01570	GRAS	GRAS	SbJAZ1	2002	2021	+	1.19E-07	0.000745	GAGAGAGAGAGAGAGAGAGA
1083.	AT2G01570	GRAS	GRAS	SbJAZ1	2004	2023	+	1.19E-07	0.000745	GAGAGAGAGAGAGAGAGAGA
1084.	AT2G01570	GRAS	GRAS	SbJAZ1	2008	2027	+	1.30E-07	0.000745	GAGAGAGAGAGAGAGAAAGA
1085.	AT2G01570	GRAS	GRAS	SbJAZ1	1990	2009	+	1.32E-07	0.000745	CAGAGAGAGAGAGAGAGAGA
1086.	AT2G01570	GRAS	GRAS	SbJAZ1	2006	2025	+	2.97E-07	0.00156	GAGAGAGAGAGAGAGAGAAA
1087.	AT2G01570	GRAS	GRAS	SbJAZ18	2070	2089	+	3.71E-07	0.00182	GAGAGAGAGAGAGAGAAGAG
1088.	AT2G01570	GRAS	GRAS	SbJAZ18	2074	2093	+	4.20E-07	0.00193	GAGAGAGAGAGAAGAGAGAG
1089.	AT2G01570	GRAS	GRAS	SbJAZ1	1988	2007	+	6.83E-07	0.00295	CACAGAGAGAGAGAGAGAGA
1090.	AT2G01570	GRAS	GRAS	SbJAZ17	2105	2124	+	9.40E-07	0.00384	AAGAGAAGGAGAGAGAGAGA
1091.	AT4G17460	HD-ZIP	HD-ZIP	SbJAZ7	935	944	+	5.37E-07	0.021	CCAATCATGG
1092.	AT4G17460	HD-ZIP	HD-ZIP	SbJAZ10	1210	1219	+	5.37E-07	0.021	CCAATCATGG
1093.	AT2G41690	HSF	HSF	SbJAZ17	835	849	-	9.47E-07	0.0663	CTTCTAGATTCTTTC
1094.	AT1G06280	LBD2	LBD	SbJAZ11	1876	1890	-	5.40E-08	0.00388	CCGAGGAAGACGGCG
1095.	AT1G65620	LBD6	LBD	SbJAZ13	1684	1704	-	2.44E-09	1.53E-04	CCGGCGCCGCGGTCCCCGCCT
1096.	AT1G65620	LBD6	LBD	SbJAZ6	429	449	-	1.17E-08	3.34E-04	TCGGCACCGCCAAATCCGCCG
1097.	AT1G65620	LBD6	LBD	SbJAZ12	1360	1380	+	1.93E-08	3.34E-04	ACCCGCCCGCCGCCTCCGGCG
1098.	AT1G65620	LBD6	LBD	SbJAZ14	2212	2232	-	2.12E-08	3.34E-04	CCGCCGGCTCCAACTCCTCCG
1099.	AT1G65620	LBD6	LBD	SbJAZ10	638	658	-	4.41E-08	0.000505	GCGCTGCCGCCTCGATCGCCG
1100.	AT1G65620	LBD6	LBD	SbJAZ12	1553	1573	+	4.82E-08	0.000505	GCCCCGCCGAGAGCGACGCCG
1101.	AT1G65620	LBD6	LBD	SbJAZ12	1557	1577	-	6.34E-08	0.00057	TCGGCGGCGTCGCTCTCGGCG
1102.	AT1G65620	LBD6	LBD	SbJAZ14	1857	1877	+	2.68E-07	0.00173	CCACCTCCTCCCCTCCCGCCC
1103.	AT1G65620	LBD6	LBD	SbJAZ17	337	357	-	2.76E-07	0.00173	GCTCTGCCGCCGCCCCCTCCC
1104.	AT1G65620	LBD6	LBD	SbJAZ8	2022	2042	+	2.96E-07	0.00173	CCTGCCCCTCCTCCTCCGCCC
1105.	AT1G65620	LBD6	LBD	SbJAZ10	635	655	-	3.23E-07	0.00173	CTGCCGCCTCGATCGCCGTCT
1106.	AT1G65620	LBD6	LBD	SbJAZ5	1734	1754	+	3.57E-07	0.00173	CCACCCCCGCCTCGCGCGCCA
1107.	AT1G65620	LBD6	LBD	SbJAZ12	1363	1383	+	3.78E-07	0.00173	CGCCCGCCGCCTCCGGCGCAC
1108.	AT1G65620	LBD6	LBD	SbJAZ14	2215	2235	-	3.86E-07	0.00173	GCGCCGCCGGCTCCAACTCCT
1109.	AT1G65620	LBD6	LBD	SbJAZ6	1920	1940	+	6.12E-07	0.00257	GACGCCCCGACTCCACCGCCG
1110.	AT1G65620	LBD6	LBD	SbJAZ10	1894	1914	+	7.39E-07	0.0029	GCGCGGGCGTCGACGCCTGCG
1111.	AT1G65620	LBD6	LBD	SbJAZ13	2160	2180	-	8.90E-07	0.00329	CTTCCTCCTCCTCCTCTGCTG
1112.	AT1G65620	LBD6	LBD	SbJAZ9	1721	1741	-	9.84E-07	0.00344	GTTTCTGCTCGGTTTCCGGCG
1113.	AT2G30340	LBD13	LBD	SbJAZ6	429	445	+	2.52E-09	1.70E-04	CGGCGGATTTGGCGGTG
1114.	AT2G30340	LBD13	LBD	SbJAZ13	1684	1700	+	9.18E-09	3.09E-04	AGGCGGGGACCGCGGCG
1115.	AT2G30340	LBD13	LBD	SbJAZ12	1364	1380	-	2.02E-07	0.00398	CGCCGGAGGCGGCGGGC

1116.	AT2G30340	LBD13	LBD	SbJAZ11	1876	1892	-	2.82E-07	0.00398	CCCCGAGGAAGACGGCG
1117.	AT2G30340	LBD13	LBD	SbJAZ6	1924	1940	-	3.35E-07	0.00398	CGGCGGTGGAGTCGGGG
1118.	AT2G30340	LBD13	LBD	SbJAZ12	1557	1573	+	3.54E-07	0.00398	CGCCGAGAGCGACGCCG
1119.	AT2G30340	LBD13	LBD	SbJAZ12	1557	1573	-	6.04E-07	0.00582	CGGCGTCGCTCTCGGCG
1120.	AT2G30340	LBD13	LBD	SbJAZ12	1367	1383	-	8.71E-07	0.00673	GTGCGCCGGAGGCGGCG
1121.	AT2G30340	LBD13	LBD	SbJAZ12	1545	1561	-	8.98E-07	0.00673	CGGCGGGGCCCGCGCCA
1122.	AT2G42430	LBD16	LBD	SbJAZ14	722	736	-	9.90E-07	0.0662	CCGGAGGAGCAGGAG
1123.	AT2G45420	LBD18	LBD	SbJAZ13	1686	1704	-	8.57E-08	0.00465	CCGGCGCCGCGGTCCCCGC
1124.	AT2G45420	LBD18	LBD	SbJAZ14	718	736	+	1.38E-07	0.00465	TCACCTCCTGCTCCTCCGG
1125.	AT2G45420	LBD18	LBD	SbJAZ12	1535	1553	-	5.00E-07	0.0112	CCCGCGCCACGTTTACCGG
1126.	AT3G26620	LBD18	LBD	SbJAZ12	1546	1560	-	4.17E-07	0.0298	GGCGGGGCCCGCGCC
1127.	AT5G63090	LOB	LBD	SbJAZ13	1682	1702	-	3.61E-09	1.73E-04	GGCGCCGCGGTCCCCGCCTCC
1128.	AT5G63090	LOB	LBD	SbJAZ14	2210	2230	-	5.30E-09	1.73E-04	GCCGGCTCCAACTCCTCCGCC
1129.	AT5G63090	LOB	LBD	SbJAZ14	2213	2233	-	3.81E-08	5.30E-04	GCCGCCGGCTCCAACTCCTCC
1130.	AT5G63090	LOB	LBD	SbJAZ13	2158	2178	-	4.14E-08	5.30E-04	TCCTCCTCCTCCTCTGCTGCT
1131.	AT5G63090	LOB	LBD	SbJAZ12	1362	1382	+	4.59E-08	0.00053	CCGCCCGCCGCCTCCGGCGCA
1132.	AT5G63090	LOB	LBD	SbJAZ12	1555	1575	+	4.88E-08	0.00053	CCCGCCGAGAGCGACGCCGCC
1133.	AT5G63090	LOB	LBD	SbJAZ8	2024	2044	+	7.71E-08	0.000614	TGCCCCTCCTCCTCCGCCCCC
1134.	AT5G63090	LOB	LBD	SbJAZ12	1365	1385	+	8.61E-08	0.000614	CCCGCCGCCTCCGGCGCACGC
1135.	AT5G63090	LOB	LBD	SbJAZ8	2021	2041	+	9.14E-08	0.000614	CCCTGCCCCTCCTCCTCCGCC
1136.	AT5G63090	LOB	LBD	SbJAZ6	1922	1942	+	9.42E-08	0.000614	CGCCCCGACTCCACCGCCGGC
1137.	AT5G63090	LOB	LBD	SbJAZ10	633	653	-	1.05E-07	0.000622	GCCGCCTCGATCGCCGTCTCG
1138.	AT5G63090	LOB	LBD	SbJAZ17	335	355	-	1.47E-07	0.000779	TCTGCCGCCGCCCCCTCCCGT
1139.	AT5G63090	LOB	LBD	SbJAZ10	636	656	-	1.55E-07	0.000779	GCTGCCGCCTCGATCGCCGTC
1140.	AT5G63090	LOB	LBD	SbJAZ13	2161	2181	-	1.85E-07	0.000842	GCTTCCTCCTCCTCCTCTGCT
1141.	AT5G63090	LOB	LBD	SbJAZ6	427	447	-	1.94E-07	0.000842	GGCACCGCCAAATCCGCCGAT
1142.	AT5G63090	LOB	LBD	SbJAZ14	717	737	+	3.05E-07	0.00124	ATCACCTCCTGCTCCTCCGGA
1143.	AT5G63090	LOB	LBD	SbJAZ6	1905	1925	+	5.02E-07	0.00193	GCCTCCCTCTCCCCCGACGCC
1144.	AT5G63090	LOB	LBD	SbJAZ9	1719	1739	-	5.60E-07	0.00203	TTCTGCTCGGTTTCCGGCGCG
1145.	AT5G63090	LOB	LBD	SbJAZ12	1555	1575	-	8.60E-07	0.00295	GGCGGCGTCGCTCTCGGCGGG
1146.	AT5G63090	LOB	LBD	SbJAZ13	1823	1843	+	9.15E-07	0.00298	CCCCCCTGCCCCTCCTCCGTC
1147.	AT1G77080	MIKC_MADS	MIKC_MADS	SbJAZ10	1572	1586	-	5.71E-07	0.0445	TCTTTCTATTTCTGT
1148.	AT2G45660	MIKC_MADS	MIKC_MADS	SbJAZ1	2011	2031	-	6.27E-11	2.49E-06	CCTCTCTTTCTCTCTCTCTCT
1149.	AT2G45660	MIKC_MADS	MIKC_MADS	SbJAZ1	2005	2025	-	1.70E-10	2.49E-06	TTTCTCTCTCTCTCTCTCTCT
1150.	AT2G45660	MIKC_MADS	MIKC_MADS	SbJAZ1	1991	2011	-	3.08E-10	2.49E-06	TCTCTCTCTCTCTCTCTCTCT
1151.	AT2G45660	MIKC_MADS	MIKC_MADS	SbJAZ1	1993	2013	-	3.08E-10	2.49E-06	TCTCTCTCTCTCTCTCTCTCT
1152.	AT2G45660	MIKC_MADS	MIKC_MADS	SbJAZ1	1995	2015	-	3.08E-10	2.49E-06	TCTCTCTCTCTCTCTCTCTCT
1153.	AT2G45660	MIKC_MADS	MIKC_MADS	SbJAZ1	1997	2017	-	3.08E-10	2.49E-06	TCTCTCTCTCTCTCTCTCTCT
1154.	AT2G45660	MIKC_MADS	MIKC_MADS	SbJAZ1	1999	2019	-	3.08E-10	2.49E-06	TCTCTCTCTCTCTCTCTCTCT
1155.	AT2G45660	MIKC_MADS	MIKC_MADS	SbJAZ1	2001	2021	-	3.08E-10	2.49E-06	TCTCTCTCTCTCTCTCTCTCT
1156.	AT2G45660	MIKC_MADS	MIKC_MADS	SbJAZ1	2003	2023	-	3.08E-10	2.49E-06	TCTCTCTCTCTCTCTCTCTCT
1157.	AT2G45660	MIKC_MADS	MIKC_MADS	SbJAZ1	2009	2029	-	4.74E-10	3.46E-06	TCTCTTTCTCTCTCTCTCTCT
1158.	AT2G45660	MIKC_MADS	MIKC_MADS	SbJAZ1	2007	2027	-	2.14E-09	1.42E-05	TCTTTCTCTCTCTCTCTCTCT
1159.	AT2G45660	MIKC_MADS	MIKC_MADS	SbJAZ1	2015	2035	-	8.47E-09	4.89E-05	TCTCCCTCTCTTTCTCTCTCT
1160.	AT2G45660	MIKC_MADS	MIKC_MADS	SbJAZ1	2013	2033	-	8.73E-09	4.89E-05	TCCCTCTCTTTCTCTCTCTCT
1161.	AT2G45660	MIKC_MADS	MIKC_MADS	SbJAZ1	1989	2009	-	1.40E-08	7.31E-05	TCTCTCTCTCTCTCTCTCTGT
1162.	AT2G45660	MIKC_MADS	MIKC_MADS	SbJAZ18	2069	2089	-	2.16E-08	1.05E-04	CTCTTCTCTCTCTCTCTCTCT
1163.	AT2G45660	MIKC_MADS	MIKC_MADS	SbJAZ1	2017	2037	-	2.37E-08	1.08E-04	CCTCTCCCTCTCTTTCTCTCT
1164.	AT2G45660	MIKC_MADS	MIKC_MADS	SbJAZ17	2104	2124	-	4.77E-08	0.000204	TCTCTCTCTCTCCTTCTCTTT
1165.	AT2G45660	MIKC_MADS	MIKC_MADS	SbJAZ17	2108	2128	-	7.53E-08	0.000305	CTCCTCTCTCTCTCTCCTTCT
1166.	AT2G45660	MIKC_MADS	MIKC_MADS	SbJAZ11	1784	1804	-	9.12E-08	0.00034	TTTTTTTTTTTTTTTTTTTTG
1167.	AT2G45660	MIKC_MADS	MIKC_MADS	SbJAZ18	2073	2093	-	9.34E-08	0.00034	CTCTCTCTTCTCTCTCTCTCT
1168.	AT2G45660	MIKC_MADS	MIKC_MADS	SbJAZ17	2102	2122	-	9.81E-08	0.00034	TCTCTCTCTCCTTCTCTTTCC
1169.	AT2G45660	MIKC_MADS	MIKC_MADS	SbJAZ18	2071	2091	-	1.08E-07	0.000357	CTCTCTTCTCTCTCTCTCTCT
1170.	AT2G45660	MIKC_MADS	MIKC_MADS	SbJAZ11	1786	1806	-	2.12E-07	0.000672	CGTTTTTTTTTTTTTTTTTTT
1171.	AT2G45660	MIKC_MADS	MIKC_MADS	SbJAZ18	2086	2106	-	2.26E-07	0.000685	TTTCCCTTTCCTCCTCTCTCT
1172.	AT2G45660	MIKC_MADS	MIKC_MADS	SbJAZ11	1785	1805	-	2.35E-07	0.000685	GTTTTTTTTTTTTTTTTTTTT
1173.	AT2G45660	MIKC_MADS	MIKC_MADS	SbJAZ10	1574	1594	-	3.17E-07	0.000887	TTTCGTTCTCTTTCTATTTCT
1174.	AT2G45660	MIKC_MADS	MIKC_MADS	SbJAZ10	1580	1600	-	3.91E-07	0.00102	CGTTTCTTTCGTTCTCTTTCT
1175.	AT2G45660	MIKC_MADS	MIKC_MADS	SbJAZ11	1783	1803	-	3.91E-07	0.00102	TTTTTTTTTTTTTTTTTTTGG
1176.	AT2G45660	MIKC_MADS	MIKC_MADS	SbJAZ11	1789	1809	-	4.13E-07	0.00104	CCCCGTTTTTTTTTTTTTTTT
1177.	AT2G45660	MIKC_MADS	MIKC_MADS	SbJAZ18	2160	2180	+	4.53E-07	0.0011	CTTCTTCTTCTTCCTCCTCTT
1178.	AT2G45660	MIKC_MADS	MIKC_MADS	SbJAZ1	1987	2007	-	5.43E-07	0.00128	TCTCTCTCTCTCTCTCTGTGC
1179.	AT2G45660	MIKC_MADS	MIKC_MADS	SbJAZ11	965	985	+	5.93E-07	0.00133	CTTTTGTTTTTTTTTTTTTTG
1180.	AT2G45660	MIKC_MADS	MIKC_MADS	SbJAZ18	2163	2183	+	6.03E-07	0.00133	CTTCTTCTTCCTCCTCTTCTT
1181.	AT2G45660	MIKC_MADS	MIKC_MADS	SbJAZ11	966	986	+	6.36E-07	0.00136	TTTTGTTTTTTTTTTTTTTGT
1182.	AT1G09540	MYB61	MYB	SbJAZ4	1916	1930	+	2.85E-08	2.20E-03	ACCACCCACCACCGC
1183.	AT1G09540	MYB61	MYB	SbJAZ1	807	821	-	6.72E-08	0.0026	CCCACCCACCACAAA
1184.	AT1G68320	MYB62	MYB	SbJAZ14	1343	1357	+	7.65E-07	0.0597	GCCAAAGTTAGGCAA
1185.	AT1G74650	MYB31	MYB	SbJAZ8	1935	1949	+	3.95E-07	0.0194	CTCCCCCCACCCGCC
1186.	AT1G74650	MYB31	MYB	SbJAZ9	1508	1522	+	5.02E-07	0.0194	CAACCCCCACCTAAA
1187.	AT1G74650	MYB31	MYB	SbJAZ11	1380	1394	+	8.79E-07	0.0227	TACCAACCAACCAAC
1188.	AT2G02820	MYB88	MYB	SbJAZ9	1741	1755	+	1.18E-08	7.37E-04	CCCCCACGCGCCCGC
1189.	AT2G02820	MYB88	MYB	SbJAZ18	1430	1444	+	6.82E-08	0.00142	CACACACGCGCCACT
1190.	AT2G02820	MYB88	MYB	SbJAZ5	1911	1925	+	6.82E-08	0.00142	CACCCACGCTCCTCG

1191.	AT2G02820	MYB88	MYB	SbJAZ17	2000	2014	+	4.88E-07	0.00761	TACACCCGCGCCGCG
1192.	AT2G02820	MYB88	MYB	SbJAZ12	1384	1398	+	6.26E-07	0.0078	GCTGGACGCTCCGCC
1193.	AT2G02820	MYB88	MYB	SbJAZ16	2013	2027	+	8.44E-07	0.00876	TACGCACGCGCCACA
1194.	AT3G06490	MYB108	MYB	SbJAZ14	1341	1355	+	6.48E-07	0.0504	TTGCCAAAGTTAGGC
1195.	AT3G08500	MYB83	MYB	SbJAZ12	2209	2219	-	5.23E-07	0.0346	GTGGGTGGTGG
1196.	AT3G08500	MYB83	MYB	SbJAZ15	416	426	-	9.55E-07	0.0346	GAGGGTGGTGG
1197.	AT3G11440	MYB65	MYB	SbJAZ17	596	608	-	9.73E-07	0.076	CCCATAACCGCCA
1198.	AT3G12720	MYB65	MYB	SbJAZ2	1979	1999	-	8.93E-07	0.0693	GTTGGGGGGCTGTTTGGTGTG
1199.	AT3G28910	MYB30	MYB	SbJAZ4	1914	1928	+	2.29E-07	0.0175	CGACCACCCACCACC
1200.	AT3G28910	MYB30	MYB	SbJAZ12	2209	2223	+	6.05E-07	0.0231	CCACCACCCACCAGC
1201.	AT3G47600	C2H2	MYB	SbJAZ4	1912	1930	-	5.42E-07	0.0417	GCGGTGGTGGGTGGTCGGC
1202.	AT3G49690	MYB94	MYB	SbJAZ4	1917	1929	-	1.57E-07	0.0101	CGGTGGTGGGTGG
1203.	AT4G01680	MYB55	MYB	SbJAZ1	809	822	-	8.11E-08	0.00606	ACCCACCCACCACA
1204.	AT4G01680	MYB55	MYB	SbJAZ4	1915	1928	+	2.51E-07	0.00936	GACCACCCACCACC
1205.	AT4G01680	MYB55	MYB	SbJAZ12	2210	2223	+	4.91E-07	0.0122	CACCACCCACCAGC
1206.	AT5G62470	MYB96	MYB	SbJAZ9	1508	1525	-	1.97E-07	0.0151	ATGTTTAGGTGGGGGTTG
1207.	AT5G62470	MYB96	MYB	SbJAZ11	1380	1397	-	8.47E-07	0.0326	TTGGTTGGTTGGTTGGTA
1208.	AT1G18960	MYB_related	MYB_related	SbJAZ10	270	291	+	3.14E-09	2.22E-04	CAACAACAACAACAACAACAAC
1209.	AT1G18960	MYB_related	MYB_related	SbJAZ10	273	294	+	1.68E-07	0.00596	CAACAACAACAACAACAACAAG
1210.	AT1G18960	MYB_related	MYB_related	SbJAZ10	267	288	+	4.63E-07	0.0109	ATGCAACAACAACAACAACAAC
1211.	AT1G18960	MYB_related	MYB_related	SbJAZ8	1027	1048	+	7.24E-07	0.0128	CGCTTCCCACAACAACGCGGCC
1212.	AT3G10113	MYB83	MYB_related	SbJAZ12	867	881	-	5.62E-07	0.0439	CAAAAAAATATCTAA
1213.	AT1G01720	NAC002	NAC	SbJAZ4	1015	1036	+	8.58E-07	0.0615	TACGTGCATAACTTTGCAACTT
1214.	AT1G28470	NAC010	NAC	SbJAZ11	1208	1222	+	4.30E-07	0.0335	AACGTGACGGGCACG
1215.	AT1G32510	NAC011	NAC	SbJAZ3	1685	1705	-	7.53E-07	0.0583	GTGGCTTGGCTCAAAAGCAAA
1216.	AT1G34180	NAC016	NAC	SbJAZ11	1210	1224	+	6.53E-07	0.0506	CGTGACGGGCACGTA
1217.	AT1G34190	NAC017	NAC	SbJAZ9	1244	1262	-	7.40E-07	0.0575	AGTTTACTTGTAAATCACG
1218.	AT1G52890	NAC017	NAC	SbJAZ10	658	669	-	3.79E-08	2.61E-03	GACACGCAACCG
1219.	AT1G54330	NAC020	NAC	SbJAZ9	1239	1259	-	7.20E-07	0.056	TTACTTGTAAATCACGATACG
1220.	AT1G69490	NAC29	NAC	SbJAZ10	658	668	-	4.50E-07	0.0348	ACACGCAACCG
1221.	AT1G71930	NAC030	NAC	SbJAZ10	659	670	-	3.59E-07	0.0227	CGACACGCAACC
1222.	AT2G43000	NAC042	NAC	SbJAZ11	1071	1090	-	4.00E-07	0.0255	CGCCGGCTCTTTTCCGGCGG
1223.	AT2G43000	NAC042	NAC	SbJAZ11	1072	1091	+	8.52E-07	0.0272	CGCCGGAAAAGAGCCGGCGA
1224.	AT3G12910	NAC	NAC	SbJAZ11	1072	1088	+	1.20E-07	0.00757	CGCCGGAAAAGAGCCGG
1225.	AT3G12910	NAC	NAC	SbJAZ9	1990	2006	-	5.45E-07	0.0166	CGCCGGCACCGCACCGC
1226.	AT3G12910	NAC	NAC	SbJAZ11	1074	1090	-	7.88E-07	0.0166	CGCCGGCTCTTTTCCGG
1227.	AT3G15500	NAC3	NAC	SbJAZ10	659	679	-	3.21E-07	0.0249	GGGCGACGGCGACACGCAACC
1228.	AT3G61910	NAC66	NAC	SbJAZ10	658	670	-	2.54E-07	0.0177	CGACACGCAACCG
1229.	AT4G01550	NAC66	NAC	SbJAZ6	664	678	-	3.20E-07	0.0246	TACTTCTCCTCCAAG
1230.	AT4G17980	NAC66	NAC	SbJAZ6	662	676	+	4.31E-08	3.36E-03	TGCTTGGAGGAGAAG
1231.	AT5G04410	NAC078	NAC	SbJAZ12	1051	1062	+	4.63E-08	0.00175	CGGCACGCCACC
1232.	AT5G04410	NAC078	NAC	SbJAZ18	1545	1556	+	4.63E-08	0.00175	GCGCACGCCACG
1233.	AT5G04410	NAC078	NAC	SbJAZ11	502	513	+	7.34E-07	0.0156	CCACACGCCAAG
1234.	AT5G04410	NAC078	NAC	SbJAZ9	2147	2158	+	8.26E-07	0.0156	GCGCAAGCCACG
1235.	AT5G39610	NAC2	NAC	SbJAZ11	1210	1224	+	9.47E-07	0.0738	CGTGACGGGCACGTA
1236.	AT5G39820	Trihelix	NAC	SbJAZ9	1989	2006	-	9.11E-07	0.0568	CGCCGGCACCGCACCGCA
1237.	AT5G53950	NAC2	NAC	SbJAZ9	1244	1262	+	4.39E-07	0.0342	CGTGATTTACAAGTAAACT
1238.	AT5G62380	NAC2	NAC	SbJAZ3	1686	1706	+	7.82E-07	0.0609	TTGCTTTTGAGCCAAGCCACG
1239.	AT1G20640	Nin-like	Nin-like	SbJAZ5	2063	2077	+	1.08E-10	7.57E-06	GCAGCAGCAGCAGCC
1240.	AT1G20640	Nin-like	Nin-like	SbJAZ5	2060	2074	+	3.43E-10	1.20E-05	GCAGCAGCAGCAGCA
1241.	AT1G20640	Nin-like	Nin-like	SbJAZ5	2066	2080	+	2.14E-08	4.98E-04	GCAGCAGCAGCCAGC
1242.	AT1G20640	Nin-like	Nin-like	SbJAZ3	2077	2091	+	5.00E-08	0.000753	CAAGCAGCAGCAGCA
1243.	AT1G20640	Nin-like	Nin-like	SbJAZ5	2057	2071	+	5.38E-08	0.000753	AGAGCAGCAGCAGCA
1244.	AT1G20640	Nin-like	Nin-like	SbJAZ14	2151	2165	+	6.75E-08	0.000787	ACCGCAGCAGCTGCG
1245.	AT1G20640	Nin-like	Nin-like	SbJAZ3	2080	2094	+	3.02E-07	0.00301	GCAGCAGCAGCAAAA
1246.	AT1G20640	Nin-like	Nin-like	SbJAZ13	2229	2243	-	6.41E-07	0.00561	CTAGCAGCAGGGACG
1247.	AT1G53160	SBP	SBP	SbJAZ2	2050	2059	-	6.91E-08	0.00495	GCGTACGGCC
1248.	AT1G53160	SBP	SBP	SbJAZ13	1011	1020	-	4.13E-07	0.0148	CGGTACGGCC
1249.	AT5G18830	SBP	SBP	SbJAZ2	2050	2059	-	4.13E-07	0.0246	GCGTACGGCC
1250.	AT1G30210	TCP24	TCP	SbJAZ11	1241	1252	-	4.22E-08	2.70E-03	GAGGGGACCACC
1251.	AT1G30210	TCP24	TCP	SbJAZ18	1720	1731	-	6.97E-07	0.0223	GTGGGGACCGCC
1252.	AT1G35560	TCP23	TCP	SbJAZ18	1912	1921	+	1.04E-07	0.00626	GGGGCCCACC
1253.	AT1G35560	TCP23	TCP	SbJAZ14	1783	1792	+	5.53E-07	0.0111	GGGCCCCACC
1254.	AT1G35560	TCP23	TCP	SbJAZ14	1920	1929	+	5.53E-07	0.0111	GGGCCCCACC
1255.	AT1G35560	TCP23	TCP	SbJAZ14	1917	1926	-	7.76E-07	0.0114	GGGGCCCGCC
1256.	AT1G35560	TCP23	TCP	SbJAZ7	788	797	-	9.48E-07	0.0114	GGGACCCACA
1257.	AT1G53230	TCP3	TCP	SbJAZ11	1240	1252	-	5.31E-08	0.00347	GAGGGGACCACCG
1258.	AT1G53230	TCP3	TCP	SbJAZ18	1910	1922	+	5.89E-07	0.0191	GCGGGGCCCACCG
1259.	AT1G53230	TCP3	TCP	SbJAZ18	1719	1731	-	8.77E-07	0.0191	GTGGGGACCGCCC
1260.	AT1G67260	TCP1	TCP	SbJAZ18	1909	1938	+	8.19E-10	3.68E-05	GGCGGGGCCCACCGCCCACCCGACGCCCCC
1261.	AT1G67260	TCP1	TCP	SbJAZ14	1780	1809	+	1.31E-09	3.68E-05	CGCGGGCCCCACCTGTCCCGTCTGGAAGCG
1262.	AT1G67260	TCP1	TCP	SbJAZ14	1763	1792	-	3.08E-09	5.78E-05	GGTGGGGCCCGCGAGCCCATGTGCGCGCTC
1263.	AT1G67260	TCP1	TCP	SbJAZ18	1892	1921	-	1.02E-08	1.43E-04	GGTGGGCCCCGCCACGGCGACCGGCTACCG
1264.	AT1G67260	TCP1	TCP	SbJAZ18	1910	1939	+	1.99E-08	2.24E-04	GCGGGGCCCACCGCCCACCCGACGCCCCCC
1265.	AT1G67260	TCP1	TCP	SbJAZ18	1616	1645	+	2.70E-08	2.54E-04	CGTGGGTCCCACTTTTCATCCTCGTGAGTC

1266.	AT1G67260	TCP1	TCP	SbJAZ14	1917	1946	+	1.17E-07	0.000943	GGCGGGCCCCACCCCACGCCCATTAAATTG
1267.	AT1G67260	TCP1	TCP	SbJAZ12	1251	1280	+	1.54E-07	0.00108	CGTGGGACCCGTTCCCCGCGCAGAGAATCC
1268.	AT1G67260	TCP1	TCP	SbJAZ6	1941	1970	+	2.15E-07	0.00134	GCCGGGGCCACCGCGCCCCGACGCGGCCTC
1269.	AT1G67260	TCP1	TCP	SbJAZ12	1531	1560	-	3.41E-07	0.00192	GGCGGGGCCCGCGCCACGTTTACCGGTTCC
1270.	AT1G67260	TCP1	TCP	SbJAZ12	1548	1577	+	4.33E-07	0.00222	CGCGGGCCCCGCCGAGAGCGACGCCGCCGA
1271.	AT1G67260	TCP1	TCP	SbJAZ14	1900	1929	-	6.84E-07	0.00314	GGTGGGGCCCGCCATGAATGCTTCGAGAGA
1272.	AT1G67260	TCP1	TCP	SbJAZ6	730	759	-	7.28E-07	0.00314	TGTGGGTCCTGCCTATCATCAGCTGGGGTC
1273.	AT1G67260	TCP1	TCP	SbJAZ18	1599	1628	-	8.02E-07	0.00314	AGTGGGACCCACGAGGGGGACTGCGTGATT
1274.	AT1G67260	TCP1	TCP	SbJAZ18	603	632	+	8.51E-07	0.00314	TGTGGGCCCTAACATGCACTGTGAGGGAGC
1275.	AT1G67260	TCP1	TCP	SbJAZ10	29	58	+	9.09E-07	0.00314	TGTGGGCCCTCGCGTCGTGTCAAGGGCCAC
1276.	AT1G67260	TCP1	TCP	SbJAZ11	1829	1858	-	9.47E-07	0.00314	GGTGGGCGCCGACTAGTGCGCTGCGGCGGG
1277.	AT1G69690	TCP15	TCP	SbJAZ14	1916	1928	+	1.85E-08	8.19E-04	TGGCGGGCCCCAC
1278.	AT1G69690	TCP15	TCP	SbJAZ18	1910	1922	-	2.54E-08	8.19E-04	CGGTGGGCCCCGC
1279.	AT1G69690	TCP15	TCP	SbJAZ18	1615	1627	+	7.37E-08	0.00133	TCGTGGGTCCCAC
1280.	AT1G69690	TCP15	TCP	SbJAZ18	1617	1629	-	8.23E-08	0.00133	AAGTGGGACCCAC
1281.	AT1G69690	TCP15	TCP	SbJAZ14	1779	1791	+	1.25E-07	0.00161	TCGCGGGCCCCAC
1282.	AT1G69690	TCP15	TCP	SbJAZ14	1781	1793	-	2.94E-07	0.00239	AGGTGGGGCCCGC
1283.	AT1G69690	TCP15	TCP	SbJAZ14	1918	1930	-	2.94E-07	0.00239	GGGTGGGGCCCGC
1284.	AT1G69690	TCP15	TCP	SbJAZ18	1908	1920	+	2.96E-07	0.00239	TGGCGGGGCCCAC
1285.	AT1G69690	TCP15	TCP	SbJAZ12	1547	1559	+	8.63E-07	0.0062	GCGCGGGCCCCGC
1286.	AT1G72010	TCP22	TCP	SbJAZ18	1910	1922	-	2.31E-08	1.13E-03	CGGTGGGCCCCGC
1287.	AT1G72010	TCP22	TCP	SbJAZ18	1617	1629	-	3.54E-08	1.13E-03	AAGTGGGACCCAC
1288.	AT1G72010	TCP22	TCP	SbJAZ18	1615	1627	+	8.93E-08	0.0018	TCGTGGGTCCCAC
1289.	AT1G72010	TCP22	TCP	SbJAZ14	1781	1793	-	1.30E-07	0.0018	AGGTGGGGCCCGC
1290.	AT1G72010	TCP22	TCP	SbJAZ14	1918	1930	-	1.41E-07	0.0018	GGGTGGGGCCCGC
1291.	AT1G72010	TCP22	TCP	SbJAZ14	1916	1928	+	2.60E-07	0.00276	TGGCGGGCCCCAC
1292.	AT1G72010	TCP22	TCP	SbJAZ14	1779	1791	+	3.55E-07	0.00323	TCGCGGGCCCCAC
1293.	AT1G72010	TCP22	TCP	SbJAZ12	1250	1262	+	5.90E-07	0.00437	ACGTGGGACCCGT
1294.	AT1G72010	TCP22	TCP	SbJAZ12	1547	1559	+	6.69E-07	0.00437	GCGCGGGCCCCGC
1295.	AT1G72010	TCP22	TCP	SbJAZ7	787	799	+	6.86E-07	0.00437	CTGTGGGTCCCGT
1296.	AT2G45680	TCP9	TCP	SbJAZ18	1909	1929	-	1.05E-08	4.61E-04	GGGTGGGCGGTGGGCCCCGCC
1297.	AT2G45680	TCP9	TCP	SbJAZ18	1608	1628	+	1.63E-08	4.61E-04	GTCCCCCTCGTGGGTCCCACT
1298.	AT2G45680	TCP9	TCP	SbJAZ14	1909	1929	+	2.46E-08	4.61E-04	GCATTCATGGCGGGCCCCACC
1299.	AT2G45680	TCP9	TCP	SbJAZ14	1772	1792	+	2.84E-08	4.61E-04	CATGGGCTCGCGGGCCCCACC
1300.	AT2G45680	TCP9	TCP	SbJAZ12	1540	1560	+	5.45E-08	0.000707	AAACGTGGCGCGGGCCCCGCC
1301.	AT2G45680	TCP9	TCP	SbJAZ14	1917	1937	-	9.63E-08	0.00104	GGGCGTGGGGTGGGGCCCGCC
1302.	AT2G45680	TCP9	TCP	SbJAZ18	1616	1636	-	1.55E-07	0.00143	GGATGAAAAGTGGGACCCACG
1303.	AT2G45680	TCP9	TCP	SbJAZ18	1547	1567	+	2.19E-07	0.00178	GCACGCCACGCGGGCTCCACC
1304.	AT2G45680	TCP9	TCP	SbJAZ6	747	767	-	2.64E-07	0.0019	CCAAATTATGTGGGTCCTGCC
1305.	AT2G45680	TCP9	TCP	SbJAZ14	1780	1800	-	4.54E-07	0.00275	ACGGGACAGGTGGGGCCCGCG
1306.	AT2G45680	TCP9	TCP	SbJAZ18	1901	1921	+	4.67E-07	0.00275	GTCGCCGTGGCGGGGCCCACC
1307.	AT2G45680	TCP9	TCP	SbJAZ13	1685	1705	-	5.63E-07	0.00304	CCCGGCGCCGCGGTCCCCGCC
1308.	AT2G45680	TCP9	TCP	SbJAZ7	780	800	+	6.75E-07	0.00322	TTCGTAGCTGTGGGTCCCGTT
1309.	AT2G45680	TCP9	TCP	SbJAZ12	1251	1271	-	7.19E-07	0.00322	GCGCGGGGAACGGGTCCCACG
1310.	AT2G45680	TCP9	TCP	SbJAZ18	1793	1813	-	7.44E-07	0.00322	CGATGACACGCGGGCTCCGCC
1311.	AT2G45680	TCP9	TCP	SbJAZ12	1548	1568	-	8.20E-07	0.00332	TCGCTCTCGGCGGGGCCCGCG
1312.	AT2G45680	TCP9	TCP	SbJAZ4	1772	1792	-	9.14E-07	0.00349	CGACTGGCTGTGGACCCCGTG
1313.	AT3G02150	TCP13	TCP	SbJAZ18	1910	1922	+	1.74E-07	0.00437	GCGGGGCCCACCG
1314.	AT3G02150	TCP13	TCP	SbJAZ14	1916	1928	-	2.53E-07	0.00437	GTGGGGCCCGCCA
1315.	AT3G02150	TCP13	TCP	SbJAZ18	1719	1731	-	2.71E-07	0.00437	GTGGGGACCGCCC
1316.	AT3G02150	TCP13	TCP	SbJAZ11	1240	1252	-	2.82E-07	0.00437	GAGGGGACCACCG
1317.	AT3G02150	TCP13	TCP	SbJAZ14	1779	1791	-	3.34E-07	0.00437	GTGGGGCCCGCGA
1318.	AT3G02150	TCP13	TCP	SbJAZ13	1686	1698	+	5.16E-07	0.00564	GCGGGGACCGCGG
1319.	AT3G02150	TCP13	TCP	SbJAZ4	837	849	+	8.87E-07	0.00831	GTTGGGTCCACAA
1320.	AT3G27010	TCP20	TCP	SbJAZ14	1781	1801	-	5.85E-10	3.82E-05	GACGGGACAGGTGGGGCCCGC
1321.	AT3G27010	TCP20	TCP	SbJAZ18	1617	1637	-	6.62E-09	2.03E-04	AGGATGAAAAGTGGGACCCAC
1322.	AT3G27010	TCP20	TCP	SbJAZ14	1918	1938	-	9.34E-09	2.03E-04	TGGGCGTGGGGTGGGGCCCGC
1323.	AT3G27010	TCP20	TCP	SbJAZ18	1900	1920	+	2.04E-08	3.33E-04	GGTCGCCGTGGCGGGGCCCAC
1324.	AT3G27010	TCP20	TCP	SbJAZ18	1607	1627	+	1.42E-07	0.00164	AGTCCCCCTCGTGGGTCCCAC
1325.	AT3G27010	TCP20	TCP	SbJAZ12	1242	1262	+	1.50E-07	0.00164	CAGGAAGCACGTGGGACCCGT
1326.	AT3G27010	TCP20	TCP	SbJAZ18	1910	1930	-	2.89E-07	0.0027	CGGGTGGGCGGTGGGCCCCGC
1327.	AT3G27010	TCP20	TCP	SbJAZ14	1771	1791	+	4.82E-07	0.0037	ACATGGGCTCGCGGGCCCCAC
1328.	AT3G27010	TCP20	TCP	SbJAZ12	1549	1569	-	5.10E-07	0.0037	GTCGCTCTCGGCGGGGCCCGC
1329.	AT3G27010	TCP20	TCP	SbJAZ13	1676	1696	+	9.74E-07	0.00636	CGCCGGGGAGGCGGGGACCGC
1330.	AT3G45150	TCP16	TCP	SbJAZ4	837	847	+	1.03E-07	0.00344	GTTGGGTCCAC
1331.	AT3G45150	TCP16	TCP	SbJAZ18	1910	1920	+	1.09E-07	0.00344	GCGGGGCCCAC
1332.	AT3G45150	TCP16	TCP	SbJAZ4	1773	1783	+	9.04E-07	0.0191	ACGGGGTCCAC
1333.	AT3G47620	TCP14	TCP	SbJAZ14	1781	1800	-	6.02E-09	3.93E-04	ACGGGACAGGTGGGGCCCGC
1334.	AT3G47620	TCP14	TCP	SbJAZ14	1918	1937	-	2.88E-08	9.40E-04	GGGCGTGGGGTGGGGCCCGC
1335.	AT3G47620	TCP14	TCP	SbJAZ18	1617	1636	-	4.32E-08	9.40E-04	GGATGAAAAGTGGGACCCAC
1336.	AT3G47620	TCP14	TCP	SbJAZ12	1243	1262	+	1.72E-07	0.00281	AGGAAGCACGTGGGACCCGT
1337.	AT3G47620	TCP14	TCP	SbJAZ18	1910	1929	-	5.17E-07	0.00675	GGGTGGGCGGTGGGCCCCGC
1338.	AT3G47620	TCP14	TCP	SbJAZ18	1556	1575	-	8.10E-07	0.00882	GGTGGGGCGGTGGAGCCCGC
1339.	AT5G08070	TCP17	TCP	SbJAZ18	1721	1731	+	1.38E-07	0.00467	GCGGTCCCCAC
1340.	AT5G08070	TCP17	TCP	SbJAZ11	1242	1252	+	2.06E-07	0.00467	GTGGTCCCCTC

1341.	AT5G08070	TCP17	TCP	SbJAZ18	1910	1920	-	2.64E-07	0.00467	GTGGGCCCCGC
1342.	AT5G08070	TCP17	TCP	SbJAZ14	1781	1791	+	3.50E-07	0.00467	GCGGGCCCCAC
1343.	AT5G08070	TCP17	TCP	SbJAZ14	1918	1928	+	3.50E-07	0.00467	GCGGGCCCCAC
1344.	AT5G08070	TCP17	TCP	SbJAZ13	1686	1696	-	7.17E-07	0.00798	GCGGTCCCCGC
1345.	AT5G08330	TCP11	TCP	SbJAZ18	1908	1921	-	1.32E-08	8.94E-04	GGTGGGCCCCGCCA
1346.	AT5G08330	TCP11	TCP	SbJAZ18	1615	1628	-	4.47E-08	0.00152	AGTGGGACCCACGA
1347.	AT5G08330	TCP11	TCP	SbJAZ18	1616	1629	+	8.54E-08	0.00193	CGTGGGTCCCACTT
1348.	AT5G08330	TCP11	TCP	SbJAZ14	1917	1930	+	1.53E-07	0.00234	GGCGGGCCCCACCC
1349.	AT5G08330	TCP11	TCP	SbJAZ14	1780	1793	+	1.72E-07	0.00234	CGCGGGCCCCACCT
1350.	AT5G08330	TCP11	TCP	SbJAZ14	1916	1929	-	2.92E-07	0.0033	GGTGGGGCCCGCCA
1351.	AT5G08330	TCP11	TCP	SbJAZ14	1779	1792	-	3.82E-07	0.0037	GGTGGGGCCCGCGA
1352.	AT5G08330	TCP11	TCP	SbJAZ12	1548	1561	+	5.67E-07	0.00481	CGCGGGCCCCGCCG
1353.	AT5G08330	TCP11	TCP	SbJAZ6	746	759	-	7.52E-07	0.00567	TGTGGGTCCTGCCT
1354.	AT5G23280	TCP7	TCP	SbJAZ18	1617	1627	-	3.45E-08	1.28E-03	GTGGGACCCAC
1355.	AT5G23280	TCP7	TCP	SbJAZ18	1617	1627	+	7.49E-08	0.00128	GTGGGTCCCAC
1356.	AT5G23280	TCP7	TCP	SbJAZ14	1781	1791	-	7.49E-08	0.00128	GTGGGGCCCGC
1357.	AT5G23280	TCP7	TCP	SbJAZ14	1918	1928	-	7.49E-08	0.00128	GTGGGGCCCGC
1358.	AT5G23280	TCP7	TCP	SbJAZ18	1910	1920	-	1.27E-07	0.00174	GTGGGCCCCGC
1359.	AT5G23280	TCP7	TCP	SbJAZ12	1252	1262	+	4.31E-07	0.00428	GTGGGACCCGT
1360.	AT5G23280	TCP7	TCP	SbJAZ18	1910	1920	+	4.37E-07	0.00428	GCGGGGCCCAC
1361.	AT5G23280	TCP7	TCP	SbJAZ14	1781	1791	+	7.46E-07	0.00543	GCGGGCCCCAC
1362.	AT5G23280	TCP7	TCP	SbJAZ14	1918	1928	+	7.46E-07	0.00543	GCGGGCCCCAC
1363.	AT5G23280	TCP7	TCP	SbJAZ7	789	799	+	7.92E-07	0.00543	GTGGGTCCCGT
1364.	AT5G51910	TCP19	TCP	SbJAZ18	1911	1920	+	1.04E-07	0.00466	CGGGGCCCAC
1365.	AT5G51910	TCP19	TCP	SbJAZ14	1782	1791	+	2.43E-07	0.00466	CGGGCCCCAC
1366.	AT5G51910	TCP19	TCP	SbJAZ14	1919	1928	+	2.43E-07	0.00466	CGGGCCCCAC
1367.	AT5G51910	TCP19	TCP	SbJAZ14	1781	1790	-	3.82E-07	0.00466	TGGGGCCCGC
1368.	AT5G51910	TCP19	TCP	SbJAZ14	1918	1927	-	3.82E-07	0.00466	TGGGGCCCGC
1369.	AT5G51910	TCP19	TCP	SbJAZ12	1549	1558	-	4.68E-07	0.00477	CGGGGCCCGC
1370.	AT5G51910	TCP19	TCP	SbJAZ18	1617	1626	-	6.92E-07	0.00588	TGGGACCCAC
1371.	AT5G51910	TCP19	TCP	SbJAZ7	789	798	-	8.31E-07	0.00588	CGGGACCCAC
1372.	AT5G51910	TCP19	TCP	SbJAZ18	1910	1919	-	8.65E-07	0.00588	TGGGCCCCGC
1373.	AT1G76870	Trihelix	Trihelix	SbJAZ3	990	1003	-	5.21E-08	0.00406	AAACAAAACCGGCA
1374.	AT3G14180	Trihelix	Trihelix	SbJAZ11	1071	1081	+	6.01E-07	0.0407	CCGCCGGAAAA
1375.	AT5G01380	Trihelix	Trihelix	SbJAZ15	1348	1364	-	8.59E-07	0.0669	AGCACGTGTAGAATAAG
1376.	AT5G47660	Trihelix	Trihelix	SbJAZ17	1829	1843	+	4.70E-07	0.0366	TTCCGGTTTTACCGC
1377.	AT5G39760	ZF-HD	ZF-HD	SbJAZ13	767	788	-	7.23E-07	0.0562	AATAATAATTAATTACATATTT

Table S7. Aphid count data for four sorghum lines after co-cultivation with SCA

Genotype/Days	1 dpi	3 dpi	6 dpi	9 dpi
BTx623	25.5±2.23 a	41.6±4.23 a	192.7±21.03 a	622.2±51.33 a
RIL 609	22.7±1.39 a	37.5±2.67 ab	206.1±12.90 a	590.8±36.18 a
RIL 521	17.0±1.02 b	28.4±2.16 b	83.9±5.27 b	165.4±4.24 b
Tx2783	13.4±0.72 b	26.4±2.79 b	64.8±4.66 b	133.9±9.70 b
MSD	5.54	11.65	48.86	121.27
F test	***	**	***	***

Significant differences between the four lines were determined by the ANOVA and Tukey test. Mean values in a column having the different letters indicate significant differences at 0.05 level (*), 0.01 level (**), 0.001 level (***), MSD= Minimum significant difference.
